# Azobenzene-Based Photoswitchable Substrates for Advanced Mechanistic Studies of Model Haloalkane Dehalogenase Enzyme Family

**DOI:** 10.1021/acscatal.4c03503

**Published:** 2024-07-22

**Authors:** Michaela Slanska, Lenka Stackova, Sergio M. Marques, Peter Stacko, Marek Martínek, Luboš Jílek, Martin Toul, Jiri Damborsky, David Bednar, Petr Klán, Zbynek Prokop

**Affiliations:** † Loschmidt Laboratories, Department of Experimental Biology and RECETOX, Faculty of Science, Masaryk University, Brno 625 00, Czech Republic; ‡ RECETOX, Faculty of Science, Masaryk University, Brno 625 00, Czech Republic; § Department of Chemistry, Faculty of Science, Masaryk University, Brno 625 00, Czech Republic; ∥ International Clinical Research Centre, St. Ann’s Hospital, Brno 625 00, Czech Republic

**Keywords:** photoswitch, azobezene, enzyme, transient kinetics, mechanism, haloalkane dehalogenase, time-resolved spectroscopy

## Abstract

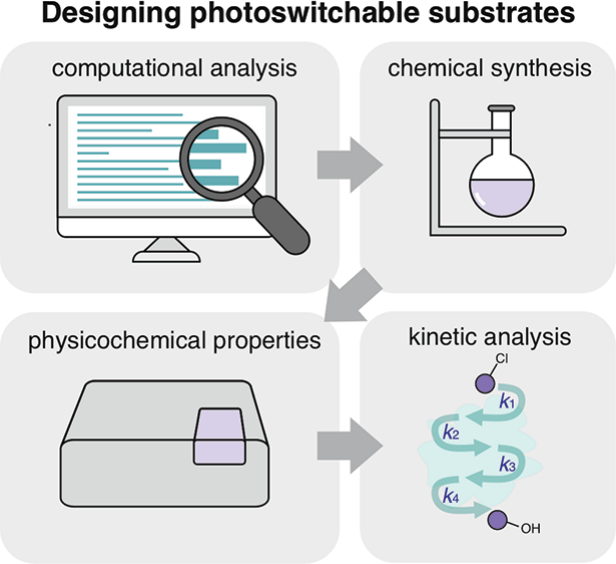

The engineering of efficient enzymes for large-scale production of industrially relevant compounds is a challenging task. Utilizing rational protein design, which relies on a comprehensive understanding of mechanistic information, holds significant promise for achieving success in this endeavor. Pre-steady-state kinetic measurements, obtained either through fast-mixing techniques or photoswitchable substrates, provide crucial mechanistic insights. The latter approach not only furnishes mechanistic clarity but also affords real-time structural elucidation of reaction intermediates via time-resolved femtosecond crystallography. Unfortunately, only a limited number of such valuable mechanistic probes are available. To address this gap, we applied a multidisciplinary approach, including computational analysis, chemical synthesis, physicochemical property screening, and enzyme kinetics to identify promising candidates for photoswitchable probes. We demonstrate the approach by designing an azobenzene-based photoswitchable substrate tailored for haloalkane dehalogenases, a prototypic class of enzymes pivotal in developing computational tools for rational protein design. The probe was subjected to steady-state and pre-steady-state kinetic analysis, which revealed new insights about the catalytic behavior of the model biocatalysts. We employed laser-triggered *Z*-to-*E* azobenzene photoswitching to generate the productive isomer *in situ*, opening avenues for advanced mechanistic studies using time-resolved femtosecond crystallography. Our results not only pave the way for the mechanistic understanding of this model enzyme family, incorporating both kinetic and structural dimensions, but also propose a systematic approach to the rational design of photoswitchable enzymatic substrates.

## 1 Introduction

Owing to the increase in environmental awareness, biocatalysis is gaining considerable attention as a tool in green and sustainable chemistry, enabling environmentally friendly and economically attractive production of pharmaceuticals and other chemicals. Stoichiometric inorganic catalysts present a significant cause of waste and, as such, are being replaced by biodegradable, nontoxic enzymes, which can catalyze reactions under mild conditions with high specificity and even enantioselectivity.^1,2^ Modification of natural enzymes is usually required to improve their properties for sustainable production. Despite its significant development over the past decades, protein engineering still presents the most time-consuming step in implementing enzymes in pharmaceutical production.^3^ Unlike directed evolution, the rational design of enzymes does not require robust screening platforms and is, therefore, a promising strategy that could speed up this process. To effectively use this approach, a detailed understanding of the structure–function relationships and the rate-limiting steps in the kinetic mechanism of the enzyme of interest is needed.

Unfortunately, reaction mechanisms cannot be analyzed in great detail using steady-state kinetics. Instead, pre-steady-state kinetics, which follow events in a much smaller time course (e.g., milliseconds) before the “steady-state” is reached, can inform us about individual reaction steps, structural changes, or the rate-limiting step.^4,5^ The most common approaches for analyzing fast kinetics are rapid mixing methods.^6^ They enable an immediate combination of small sample volumes, allowing kinetic measurements in the millisecond time scale. These methods require complicated equipment to trigger the reaction. The mixing phase can damage fragile samples because of the sudden change of environment and pressure and the high shear forces involved during the mixing phase. Moreover, faster events may be lost in the intrinsic dead time of these methods.^7^ An interesting alternative is photoactivation, a technique enabling the reaction of interest to be triggered by light without adding additional reagents or applying rapid mixing techniques, opening possibilities of combination with crystallographic analyses. Additionally, triggering the reaction with a short laser pulse can reduce the dead time of the measurement. The method is based on the application of photoswitchable probes, such as azobenzene-based molecules. Such probes can be reversibly photoswitched between the active and inactive states, as has already been demonstrated for various purposes.^8−13^ The elemental photoisomerization step of azobenzenes typically occurs on the picosecond time scale, which makes it a powerful tool for studying fast, otherwise difficult-to-detect processes.^7,14−17^ The photoisomerization efficiency depends on the molar absorption coefficient and quantum yield ratio of both isomers as well as the light source intensity. If the method is efficient enough, a laser pulse can generate a high concentration of one isomer, enabling the subsequent, much slower processes to be analyzed in great detail.

**Figure 1:**
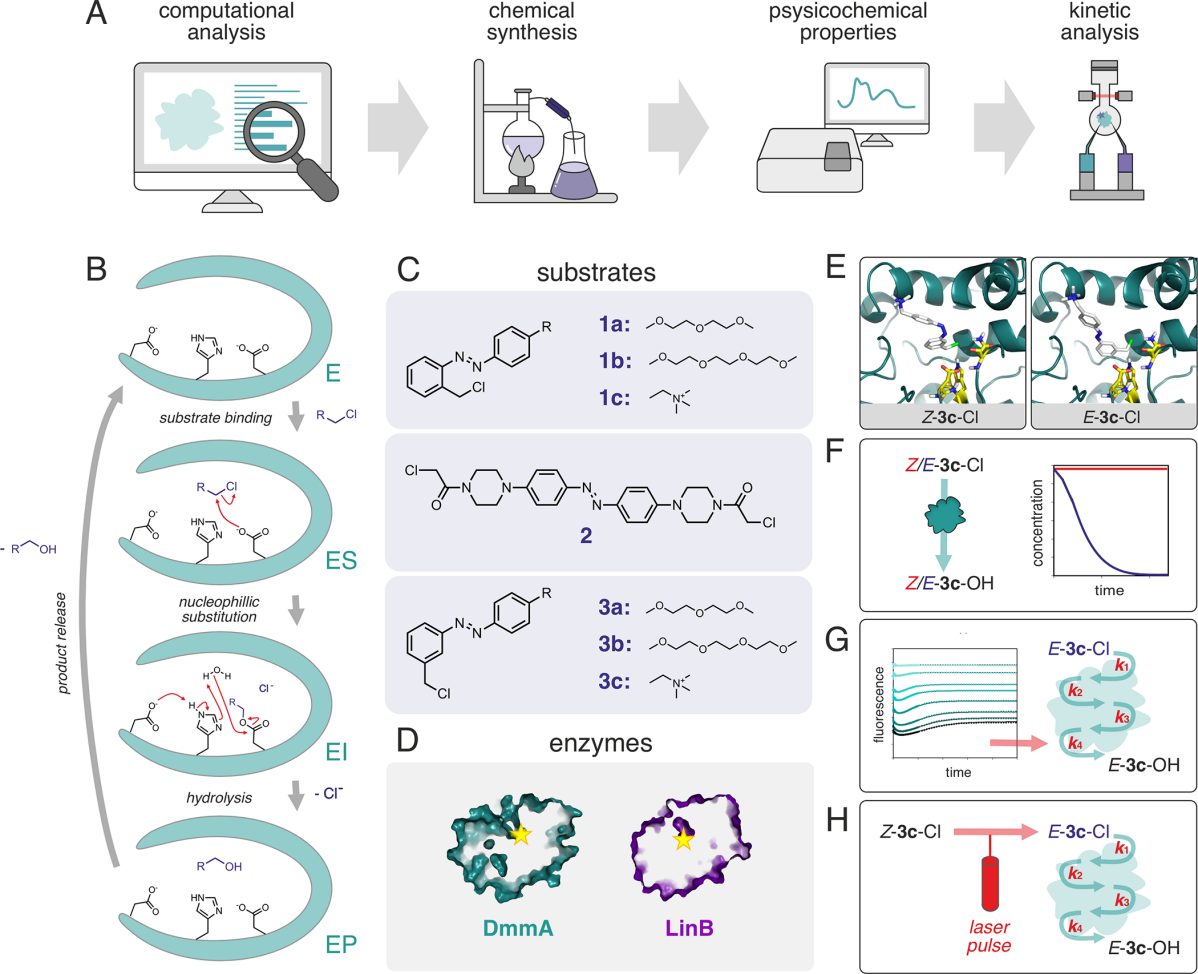
General workflow for the development of photoswitchable probes demonstrated on the model HLDs enzymes. (A) A multidisciplinary methodological approach for designing photoswitchable probes of enzymatic catalysis. (B) A four-step catalytic mechanism of haloalkane dehalogenases.^23,24^ The haloalkane substrate binds to the active site (ES). An aspartate residue facilitates nucleophilic substitution (S_N_2), leading to the formation of a covalent alkyl-enzyme intermediate (EI), while the halide ion is stabilized by residues in the halide-binding site (not illustrated). The intermediate is hydrolyzed by a water molecule activated by a catalytic histidine (EP). The alcohol product is released from the active site. (C) A set of chlorinated azobenzene compounds were designed and synthesized as substrates (the *E* isomers are shown). (D) HLDs with varying active site accessibility from the surface to the buried active site (the proteins are displayed as a cross-section of their surface, with the active sites indicated by the yellow stars). (E) Molecular docking assessed the difference in the reactive binding modes of *Z* and *E*-isomers (shown for compound **3c**, represented by the white sticks, and DmmA, represented by the teal cartoon; the catalytic triad residues are represented by the yellow sticks). (F) Steady-state kinetic analysis revealed substrates with only one convertible isoform. (G) The reporting power of the selected probe was demonstrated by a pre-steady-state kinetic analysis of individual steps in the reaction mechanism of HLDs. (H) The final probe was used in a laser-triggered pre-steady-state kinetics experiment.

In this study, we report the development of an azobenzene-based photoswitchable substrate for haloalkane dehalogenases (E.C. 3.8.1.5, HLDs), a hallmark family of enzymes from the α/β-hydrolase superfamily (Figure 1). These biocatalysts, catalyzing the conversion of halogenated hydrocarbons into alcohol products, have found numerous applications in decontamination, bioremediation of environmental pollutants, biosensing, production of pharmaceuticals, or cell and protein imaging.^18^ They have been studied extensively over the past few decades and have consequently become model biocatalysts in protein engineering, e.g., for studying enzyme dynamics^19^ or the role of access pathways.^6,20^ Importantly, the amount of available information about HLDs served as a basis for developing several *in silico* methods used for the rational design of proteins.^21,22^ A photoswitchable azobenzene probe could help to improve further our understanding of the catalytic mechanism of HLDs (Figure 1B) and its limitations. Furthermore, such a probe could be used in time-resolved serial femtosecond crystallography experiments to link the mechanistic and kinetic information to structural observations. This would provide new insights into the structure–function relationships of proteins, potentially improving the reliability of current prediction tools used in rational design.

By employing a rational multidisciplinary approach that includes computational analysis, chemical synthesis, physicochemical property screening, and enzyme kinetics, we identified promising candidates for photoswitchable probes. We designed a set of chlorinated azobenzene-based compounds (Figure 1C, Figure S1) and tested them as substrates for diverse HLDs (Figure 1D). These enzymes have buried active sites with different accessibility, as revealed by the various geometries of their access tunnels (Figure S2). Initially, a docking study was performed to predict the convertibility of both isomers of each probe. Successfully synthesized substrates were then subjected to a solubility and kinetic screening. The potential of the best-performing reporter molecule was demonstrated in a mechanistic study using a rapid-mixing experimental setup. For the first time for this enzyme family, the azobenzene probe was used in a laser-triggered pre-steady-state kinetic experiment.

## 2 Experimental Section

### Synthesis

The azobenzene ligands *E*-**3a**,**b** were synthesized as described in the Supporting Information from (*E*)-4-((3-(hydroxymethyl)phenyl)diazenyl)phenol, prepared by diazotization of 3-aminobenzyl alcohol, followed by the installation of oligoethoxy solubilizing chains and the final nucleophilic substitution of the hydroxy group by chlorine (Figures S3-S12). Azobenzene *E*-**3c** was obtained through diazotization of 4-[(dimethylamino)methyl]aniline with methyl 3-nitrosobenzoate and subsequent quaternization of the dimethylamino group and the nucleophilic substitution of the hydroxy group (Figures S13-S20). *Z*-**3c** was prepared by irradiation of an *E*-**3c** solution (*c* = 4 × 10^–4^ M) in a glycine buffer (100 mM, pH = 8.6) using a Xe lamp (450 W) and a monochromator (320 nm) (Figure S21).

### Molecular Docking

Three-dimensional structures of the *E* and *Z*-isomers of the azobenzene ligands were constructed and minimized with Avogadro.^25^ Three-dimensional structures of the receptors were obtained from the RCSB Protein Data Bank:^26^ DbjA (PDB ID: 3A2M), DmmA (3U1T), DhaA (4E46), and LinB (1MJ5). MGLTools^27^ was used to prepare the files, and the molecular docking was performed by AutoDock Vina.^28^ The active site was defined as a box of 20 × 20 × 20 Å centered at the catalytic aspartate (D108 in LinB and D144 in DmmA). As reported before,^29^ the docking results were analyzed using PyMOL 1.7.4.^30^ The reactive configurations for the S_N_2 reaction were identified based on the distances and angles between the nucleophile (D144) and substrate, according to Hur et al.,^31^ and H-bonding between the reactive chlorine atom and the halide-stabilizing residues (W145 and N78) (residue numeration of DmmA). The access tunnels in those enzymes were calculated from the carboxylic oxygens of the catalytic aspartate using CAVER 3.03.^32^

### Protein Expression and Purification

Haloalkane dehalogenases DmmA and LinB were expressed in *E. coli* BL21(DE3) cells as described previously.^33^ Metal-affinity chromatography was used for purification (Figure S22). Histidine-tagged enzymes were released from their interaction with a Ni-NTA column by increasing the imidazole concentration to 300 mM. Pure enzyme was dialyzed overnight against 100 mM glycine buffer (pH 8.6). The purity of the isolated protein was checked by SDS-PAGE.

### Steady-State Kinetics

Steady-state kinetics were measured using isothermal titration calorimeter VP-ITC (Microcal, USA) at 37 °C in 100 mM glycine buffer (pH 8.6) in triplicates. In the case of probes *E*-**3a** and *E*-**3b**, 10% DMSO was added to the buffer to promote their solubility. A complete conversion experimental setup was used where the enzyme was titrated to the cell with saturated substrate solution (∼1 mM) in three injections (0.26 μM DmmA/injection, 0.1 μM LinB/injection) in 10 min intervals. Instead of the classic single injection of the enzyme into the substrate solution, we performed three consecutive injections of the enzyme to evaluate potential inaccuracies in the titration. In ITC experiments, the first injection is often inaccurate due to the lengthy initial system’s equilibration (e.g., diffusion from the reactant from the top of the needle during the incubation time). Dividing the enzyme injection into three successive additions is an elegant technical solution to obtain accurate data on the actual concentration of the enzyme and to refine the resulting estimate of *k*_cat_. During the numerical data analysis, we addressed the inaccuracy of the first enzyme titration by using information about the exact enzyme concentration from the two subsequent injections, which are not affected by the diffusion problem. Control experiments without the enzyme or without the substrate *E*-**3c** were included to account for contributions to the heat unrelated to the catalyzed conversion of the substrate (Figure S23).

**Figure 2:**
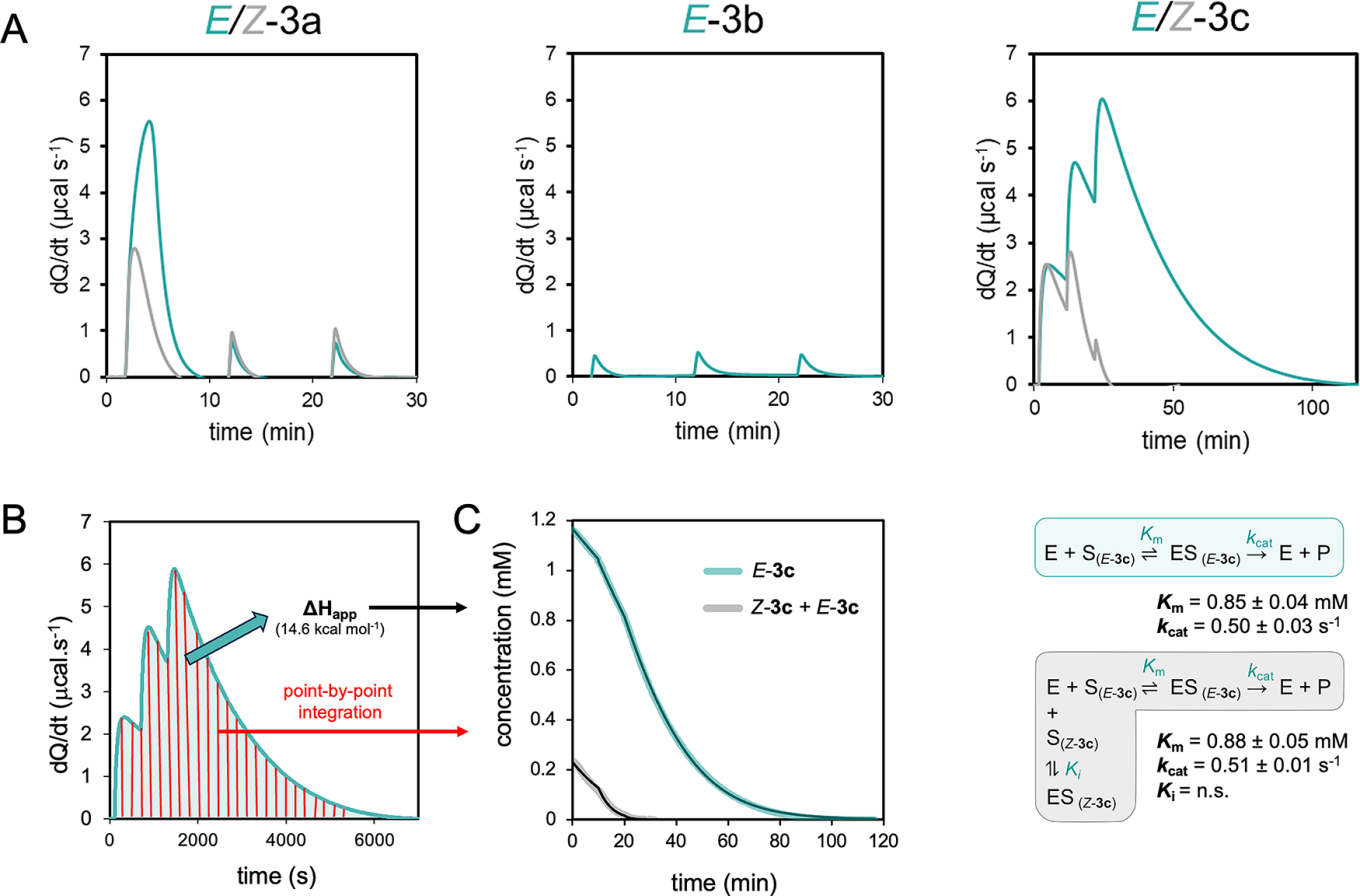
Steady-state kinetics of DmmA with selected substrates. (A) Conversion of substrates **3a** (123 μM), **3b** (182 μM), and **3c** (1170 μM) by DmmA (0.40, 0.40, and 0.26 μM per injection, respectively) monitored by ITC. The experiments were carried out at 37 °C and pH 8.6. (B) Conversion of raw ITC data to progress curves visualized for substrate *E*-**3c**. The raw data were integrated (teal) to determine the apparent molar enthalpy (Δ*H*_app_) for the reaction. The remaining substrate concentration at any given time was calculated from the integral of heat evolved (red), yielding a continuous kinetic curve. (C) The resulting progress curves, visualized for probes *E*-**3c** and *Z*-**3c** (raw data in teal and gray, respectively, fitted curves in black), were analyzed in KinTek Explorer^35^ using steady-state kinetic models to derive the steady-state kinetic constants. Δ*H*_app_ – apparent molar enthalpy, *K*_m_ – Michaelis constant, *k*_cat_ – turnover number, *K*_i_ – competitive inhibition constant, n.s. – not significant.

The raw data were integrated (Figure 2B) to give experimentally determined apparent enthalpy Δ*H*_app_ for the reaction using the equation:^34^
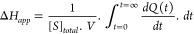
where [*S*]_total_ is the molar concentration of converted substrate, *V* is the volume of solution in the reaction cell, and *Q* is the enzyme-generated thermal power. At any given time, the reaction rate was determined from the generated thermal power using the equation:
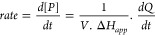
where [*P*] is the molar concentration of the product generated. The remaining substrate concentration [*S*]_t_ was calculated from the integral of heat evolved using the equation:
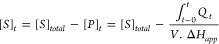


The rate plotted as a function of the substrate concentration generated a continuous kinetic curve (Figure 2C). The kinetic data were fitted using the KinTek Explorer as described below in the section ‘Data analysis and statistics’.

### Stopped-Flow Pre-Steady-State Kinetics

The pre-steady-state experiments were carried out in 100 mM glycine buffer (pH 8.6) at 37 °C. A stopped-flow SFM-300 instrument coupled with a MOS-500 spectrometer system (BioLogic, France) was used to follow the conversion of *E*-**3c** solutions of varying concentrations by 66 μM DmmA and 38 μM LinB. The reactions were initiated by rapidly mixing the substrate with the enzyme in a 1:1 ratio to reach a total volume of 150 μL with a flow rate of 16 mL s^–1^. The mixture in the reaction cuvette was continuously illuminated by an Xe lamp (280 nm selected by a monochromator, 5 nm bandwidth), and the absorbance was measured together with tryptophan fluorescence (320 nm cutoff emission filter). Each kinetic trace was calculated as the result of six individual mixing runs.

In the inhibition experiments, the enzyme (either DmmA or LinB) was premixed with either buffer (“no premix”), *Z*-**3c**, or *E*-**3c**. The incubated mixture was further rapidly mixed inside the stopped-flow instrument with additional *E*-**3c** solution, and the reaction was followed by the change of native tryptophan fluorescence as described above. The resulting concentrations in the observation cell were 41 μM for enzyme, 110 μM for premixed *Z*-**3c**/*E*-**3c**, and 220 μM for reacting *E*-**3c**. Each kinetic trace was calculated as a result of six individual mixing runs.

### Laser-Triggered Photoisomerization

The photoisomerization experiment was performed at room temperature in glycine buffer (100 mM, pH = 8.6). 75 μL of DmmA was mixed with 75 μL of *Z*-**3c** inside a Stopped-Flow instrument SFM 3000 combined with a MOS-500 spectrometer (BioLogic, France) and incubated in the observation cuvette. The mixture was subsequently repeatedly illuminated by a laser pulse (400–700 nm; duration 30 ms, Figures S24 and S25), and the eventual reaction was monitored by the change of native tryptophan fluorescence (280 nm excitation, 340 ± 13 nm band-pass filter emission, Figure 5). The resulting concentrations in the observation cell were 8 μM for DmmA and 220 μM for **3c**.

### Data Analysis and Statistics

The steady-state kinetic data, pre-steady-state kinetic data, and data obtained upon photoisomerization were fit globally with the KinTek Explorer program (KinTek, USA). This dynamic kinetic simulation program allows multiple data sets to be fit simultaneously to a single model.^35^ During the data fitting, the KinTek program applied numerical integration of rate equations from the input models, searching a set of parameters using the Bulirsch–Stoer algorithm with an adaptive step size that produces a minimum χ^2^ value calculated by using nonlinear regression based on the Levenberg–Marquardt method. Residuals were normalized by sigma value for each data point.

The reaction time course was computed numerically based on a unique set of rate constants and output factors that define the relationship between the concentrations of reactants and/or products and the observable signal. The fluorescence and absorbance data were analyzed in their raw forms by defining output observables as a sum of contributions from all relevant components of the reaction mixture. The signal was defined by eqs 1-4, where E is the enzyme, S is the substrate, ES is the enzyme–substrate complex, EI is the covalently bound intermediate, EP and EP_2_ are the enzyme–product complexes in their open and closed forms, respectively, and P is the reaction product. Factors *f* and *t* scale the fluorescence intensity and transmittance to concentration, respectively, and factors *a*, *b*, *c*, and *d* define the relative change in signal in forming ES, EI, EP, and EP_2_ complexes, respectively (Table S2). To account for slight variations in the data, enzyme or substrate concentrations were allowed to vary to make the best fits possible.

Output observables of DmmA reaction fluorescence (f, t, a, b, c, d–scaling factors).
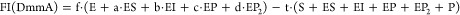
1

Output observables of DmmA reaction absorbance (f, t, a, b, c, d–scaling factors).
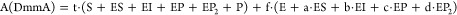
2

Output observables of LinB reaction fluorescence (f, t, a, b, c–scaling factors).
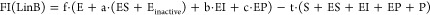
3

Output observables of LinB reaction absorbance (f, t, a, b, c–scaling factors).
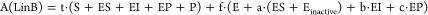
4

The standard error (s.e.) was calculated from the covariance matrix during nonlinear regression. In addition to s.e. values, a more rigorous analysis of the variation of the kinetic parameters was accomplished by confidence contour analysis with FitSpace Explorer.^36^ In this analysis, each derived parameter was held fixed at various values while the other constants were allowed to float to achieve the minimal value of χ^2^. If χ^2^ increases as the fixed parameter diverges from the optimal value obtained from the fit, the constant is well-defined. By setting a threshold of acceptable χ^2^ values (minχ^2^/χ^2^ = 0.98), the lower and upper limits of each parameter were determined.

## 3 Results and Discussion

### Design of Photoswitchable Probes

The general design of the photoswitchable probes (Figure 1C, Figure S1) was based on several requirements. The azobenzene motif was selected due to its large structural change upon photoisomerization. The chloromethyl group served as an aliphatic substrate for HLDs, and it does not cyclize intramolecularly with the azobenzene core. The installation of PEG or tetraalkylammonium pendants was proposed to increase the water solubility of the probes. The suggested structures were subjected to molecular docking calculations as substrates. During the molecular docking analysis, we aimed to identify a scenario where one isomer exhibits significant binding affinity in the reactive orientation while the opposite isomer interacts less effectively and has a low probability of adopting the reactive orientation. The binding of the unreactive isomer to the enzyme is undesirable for two reasons: (i) it may inhibit the conversion of the reactive isomer after photoswitching, and (ii) when bound, its ability to undergo photoisomerization may be reduced compared to its efficiency in free solution.

### Molecular Docking to Structurally Diverse HLDs

A docking study was performed to predict the binding efficiency and reactivity of selected HLDs (DbjA, DhaA, DmmA, and LinB) with the *E* and *Z*-isomers of the designed azobenzene derivatives (Table S1, Figure S26 and S27). Some compounds could bind in a productive mode within the active site of the studied HLDs, with the reactive carbon atom near the carboxylic oxygen atoms of the nucleophilic aspartate and the chlorine atom near the halide stabilizing residues. The rest of the molecule extended along the active site and the access tunnels connecting it to the surface. In some cases, only some or none of the geometric reactivity criteria were met, thus hinting at a possible low reactivity.

In the case of ligands **1a**-**c** (bearing an *ortho*-substitution of the reactive chloromethyl group in the azobenzene scaffold), the *Z*-isomers showed a higher propensity for reactive binding than the *E-*isomers (ΔΔ*G*_bind_^*E-Z*^ < 0). These compounds showed favorable affinities with DmmA and DbjA, especially the *Z*-isomers (Δ*G*_bind_^*Z*^**=** −7.2 – −5.2 kcal mol^–1^). The general trends in the reactive binding affinity of *Z*-isomers compared to the respective *E*-isomers ranked the substrates in the order **1c** ≈ **1a** > **1b**, making ligand **1c** the best candidate in this set for subsequent studies that favor the reaction of *Z-*isomers relative to *E*-isomers.

Compound **2** showed favorable interaction exclusively with the *Z*-isomer toward DmmA (Δ*G*_bind_^*Z*, DmmA^ = −5.6 kcal mol^–1^, Δ*G*_bind_^*E*, DmmA^ = 32.5 kcal mol^–1^). No reactive poses were observed with the other HLDs. This is probably due to the large size of substrate **2**, which is incompatible with the narrower or curved geometry of the access tunnels in the remaining HLDs (Figure S2). Therefore, substrate **2** was discarded from further testing.

Regarding compounds **3a**-**c** (with the reactive chloromethyl group at the *meta*-position of the azobenzene group), they could favorably bind within DmmA and DbjA (Δ*G*_bind_ = −8.1 – −6.0 kcal mol^–1^), but less efficiently with LinB (Δ*G*_bind_ > 0) and DhaA (Δ*G*_bind_ = −4.8 – + 1.1 kcal mol^–1^). In most cases, *E*-isomers of compounds **3a**-**c** showed high propensities for the reactive binding mode than *Z*-isomers. This was either because no reactive configuration was detected by docking (e.g., with LinB), it was not ideal for the reactivity (i.e., one or more geometric requirements were missing, like for DmmA and DbjA), or the binding energy was more favorable for *E*-isomers than for *Z*-isomers (e.g., ΔΔ*G*_bind_^*E*-**3a**-*Z*-**3a**, DbjA^ = 1.8). The predicted binding affinities of compounds *E*-**3a**-**c** with DmmA ranked as **3a** > **3b** ≈ **3c**, but for LinB, it was 3a ≈ 3c ≫ 3b, and with DbjA **3c** was ranked the best. Both E*-***3a** and *E-***3c** could be similarly suitable as substrates.

Overall, docking analysis indicated compounds **1** and **3** as equally good substrates for HLDs. Considering the possible side reactivity of compound **1**, such as photochemical/thermal intramolecular cyclization to indazoles^37^ related to the *ortho*-substituents, we preferred compounds **3a**-**c** for synthesis and further characterization.

### Synthesis and Characterization of Substrates **3a**-**c**

The selected substrates **3a**-**c** were synthesized and characterized. Compounds **3** in PBS display strong absorption bands at ∼320 nm (Figures S28-S33). Their solubilities were determined using UV/vis spectroscopy. Substrates *E*-**3a** and *E*-**3b** could only be dissolved in glycine buffer with 10% (v/v) of DMSO at much lower concentrations (120 and 180 μM, respectively). *E*-**3c** was the only compound that did not require the use of a cosolvent to be dissolved in aqueous media to a maximum concentration of ∼1.1 mM. Moreover, the solution composition in the photostationary state during the photoisomerization of *E*-**3c** to *Z*-**3c** at 320 nm is 25:75 (*E*-**3c**/*Z*-**3c**, n/n), which greatly favors the *Z*-isomer (Figures S34 and S35). The *E*-**3c**/*Z*-**3c** concentration ratio does not change over tens of hours (Figure S32), which justifies the experimental method and the selected compounds we used.

### Steady-State Kinetics of *E*-**3a** and *E*-**3c** Conversion by HLDs

To investigate the potential of the synthesized compounds **3a**-**c** as substrates for HLDs, their convertibility by DmmA was analyzed by isothermal titration calorimetry (ITC). Figure 2A shows the heat flow as a result of three injections of the enzyme into substrate solutions of a maximum achievable concentration. This complete conversion experimental setup enables the identification of all possible scenarios: (i) no interaction, (ii) specific binding, and (iii) catalytic conversion. Injecting the enzyme into the substrate was used instead of more conventional injections of the substrate into an enzyme to maximize the substrate concentration in the reaction mixture and approach enzyme saturation at the beginning of the experiment, which was crucial for the correct determination of the steady-state kinetic constants, *k*_cat_ and *K*_m_.

*E*-**3b** produced no sign of reaction or binding, with only solvation heat produced after each injection. The other two substrates could undergo the reaction, but *E*-**3a** was converted completely after the first injection of DmmA due to its low concentration, which was limited by solubility.

In the case of substrate **3c**, raw ITC data were converted to progress curves to calculate steady-state kinetic constants (Figure 2B). Integration of the area under the baseline-corrected raw data curves and the known concentrations of converted substrate (quantified by UV/vis spectrometry) provided information on the apparent molar enthalpy of substrate binding and conversion (Δ*H*_app_). Follow-up by point-by-point integration allowed the transformation of calorimetric data into full progress curves, which were fit to the steady-state kinetic models (Figure 2C) using numerical integration of rate equations. This approach increased the accuracy of parameter estimates and reduced the errors of conventional initial velocity analysis.^38^ Since the substrates were synthesized as *E*-isomers, the kinetic analysis comprehensively characterized the *E*-isomer conversion (teal curve in Figure 2C). Upon photoswitching to *Z*-isomers, residual *E*-isomers persisted in the solution. Kinetic examination of the substrates after photoconversion to *Z*-isomers (gray curve in Figure 2C) verified that the heat released and the resulting kinetic parameters were consistent with the residual amount of *E*-isomers in the solution after photoswitching. Furthermore, the kinetic analysis indicated that *Z*-isomers neither contributed to the reaction signal nor induced any inhibitory effects. The competitive inhibitory effect of the *Z-*isomer (*K*_i_) was statistically insignificant, even though it had a significant concentration excess over the reactive *E*-isomer. These findings are consistent with the *in silico* analyses indicating the unfavorable conformation of *Z*-isomers for interacting with the enzyme. These results thus confirm the potential of the **3c** probe for photoisomerization-triggered experiments.

Similarly, the kinetic analysis of the *E/Z*-**3a** reaction with DmmA revealed that the *Z*-isomer of substrate **3a** binds to the enzyme’s active site and functions as a competitive inhibitor for the *E*-**3a** reaction. Furthermore, the reaction involving the pure *E*-**3a** isomer exhibited substrate inhibition (**Figure S36**). Low solubility and undesirable inhibitory effects preclude this substrate from being an appropriate candidate for photoactivation experiments. Product inhibition, assessed as part of the steady-state kinetic analysis, was insignificant for both substrates **3a** and **3c**.

### Detailed Kinetic Analysis of Substrate **3c**

**Figure 3:**
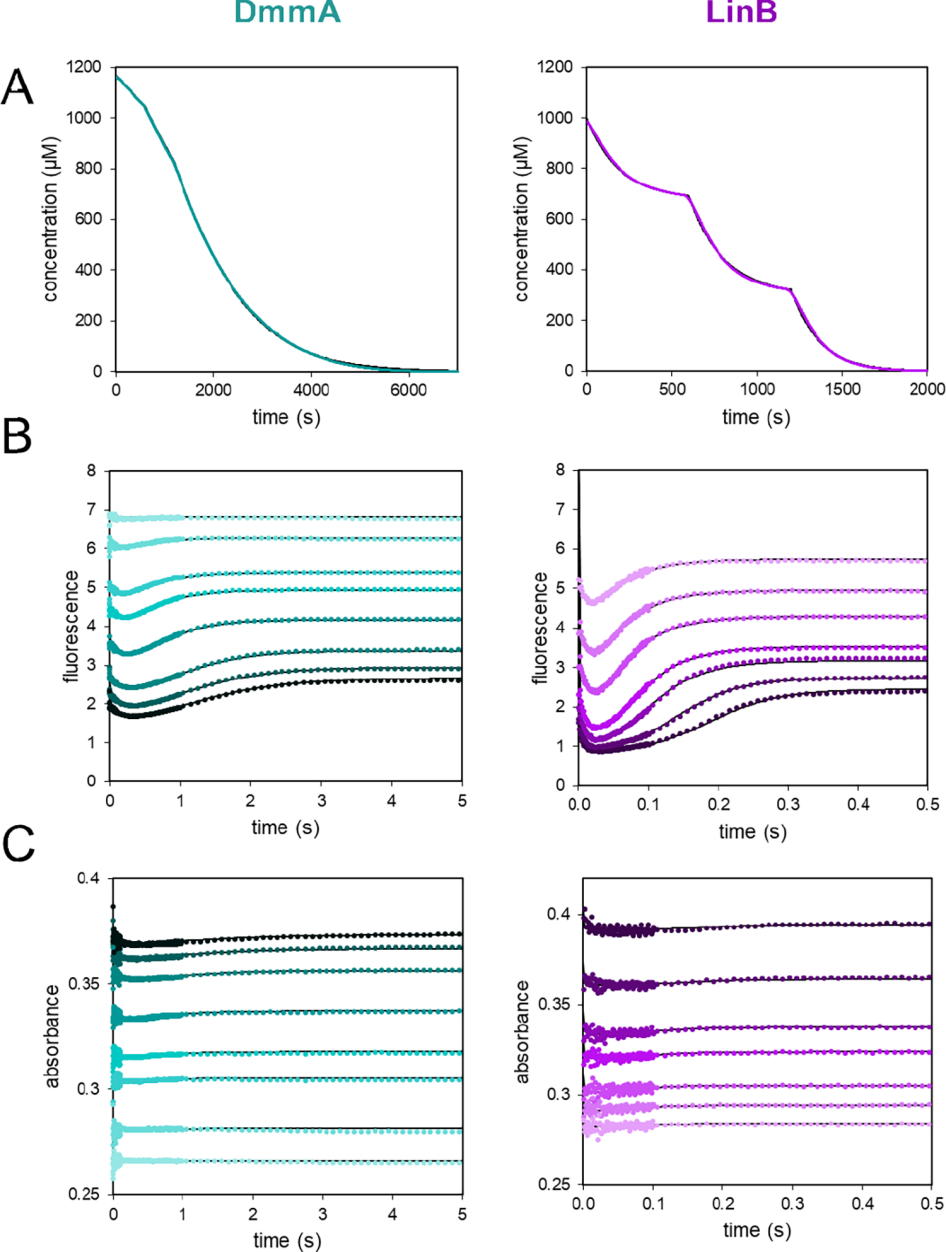
Global numerical analysis of the kinetic mechanism of DmmA and LinB with *E*-**3c**. (A) Steady-state kinetics of DmmA (0.26 μM per injection) and LinB (0.1 μM per injection) with 1.17 mM and 1 mM *E*-**3c**, respectively, measured by ITC. (B) Pre-steady-state kinetics of 66 μM DmmA and 38 μM LinB with 9–59 μM and 54–110 μM *E*-**3c**, respectively, measured by tryptophan fluorescence quenching (excitation 280 nm, emission 320 nm cutoff). (C) Presteady-state kinetics of 66 μM DmmA and 38 μM LinB with 9–59 μM and 54–110 μM *E*-**3c**, respectively, measured by the absorbance of the substrate and the enzyme (280 nm). The experiment was carried out at 37 °C and pH 8.6.

**Figure 4:**
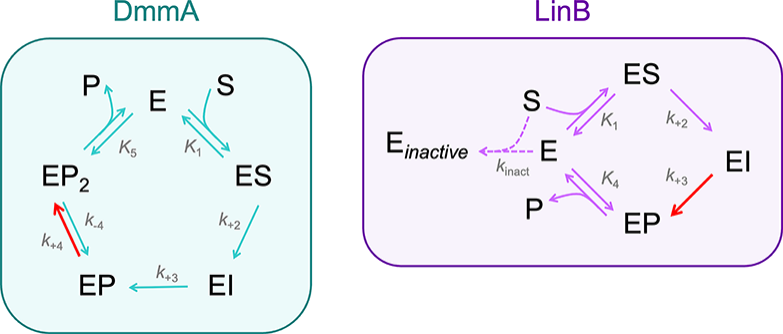
Mechanisms of *E*-**3c** conversion by DmmA and LinB. The rate-limiting steps are highlighted in red. E – free enzyme; ES – enzyme-substrate complex; EI – enzyme-intermediate complex; EP – enzyme–product complex; EP_2_ – modified enzyme–product complex; E_*inactive*_ – inactivated enzyme; S – substrate; P – product.

Due to its high solubility in aqueous solutions and its convertibility by DmmA, azobenzene **3c** was selected as the most promising substrate for further analysis. Its *E*-isomer was used to analyze the kinetic mechanism of enzymes DmmA and LinB to determine its potential in kinetic experiments (Figures 3 and 4). These HLDs were chosen because of their distinct differences in structure, kinetics, and dynamic behavior, yet they have both previously shown conversion of bulky substrates.^39^

First, the reactions were analyzed by ITC, following three injections of the enzyme into the substrate solutions. (Figure 3A). A typical progress curve leading to total conversion was observed in the case of DmmA, with each injection of enzyme increasing the initial rate. In the case of LinB, the reaction was inactivated during the conversion with a clear reignition after repeated injection of the fresh enzyme. This inactivation cannot be explained by any reversible inhibitory binding of the reaction product or substrate. A model of slow irreversible inhibition of the enzyme by the substrate was clearly indicated, providing the best fit during the global kinetic data analysis. Pre-steady-state kinetics were followed by measuring changes in the fluorescence (Figure 3B) and absorbance (Figure 3C) signals of the reaction mixture, following the rapid mixing of the enzyme with the probe. The fluorescence signal stems from a halide-stabilizing tryptophan residue located in the active site of HLDs (Figure S37), whose fluorescence responds to changes in its local environment during each step of the catalytic cycle (eqs 1 and 3). Natural tryptophan fluorescence thus provided a wealth of information on individual catalytic steps without a need for labeling. Quenching of the fluorescence intensity at the beginning of the reaction is associated with the binding of the substrate and the first reaction step (the cleavage of the halogen-carbon bond and the formation of an intermediate). The next step is the hydrolysis of the intermediate and the release of the product associated with the regeneration of the fluorescence intensity of the enzyme. All of the presented data sets were fit globally to analyze the kinetic mechanism of *E*-**3c** conversion.

In the case of the DmmA reaction, the basic four-step reaction model proposed for HLDs (Figure 1B) failed to yield satisfactory statistical fits, particularly in the product release phase. Therefore, we explored an extended two-step mechanism for the product release, previously described for HLDs^20,40,41^ (Figure 4). The enzyme–product complex (EP) underwent a slow isomerization step (EP_2_), potentially involving a conformational change, before enabling a two-step product release mechanism. This extended model demonstrated an excellent statistical agreement with the experimental data. Subsequent confidence contour analysis corroborated the efficacy of this extended catalytic cycle in accurately representing the experimental observations (Figure S38).

In the case of LinB, the *E*-**3c** conversion followed the standard four-step mechanism, with an interesting deviation: a slow irreversible inhibition by the substrate indicated in the steady-state data (Figure 4). The detailed kinetics of both reactions of a new substrate with two different enzymes thus provide interesting mechanistic observations and show an elegant opportunity to readily obtain information on the basic catalytic steps and observe the effects of enzyme dynamics or probe enzyme inhibition.

Table 1 summarizes the kinetic and equilibrium constants describing the kinetic mechanism of *E*-**3c** ligand conversion by the selected HLDs. The rate-limiting step in the catalytic cycle of DmmA is the conformational change of the enzyme–product complex (EP) into the form capable of product release (EP_2_) (*k*_+4_ = 1.42 ± 0.08 s^–1^). In the case of LinB, the main bottleneck in the mechanism is the hydrolytic step, leading to the conversion of the enzyme-intermediate complex (EI) to the enzyme–product complex (EP) (*k*_+3_ = 19.3 ± 0.1 s^–1^). Based on these values, catalysis by LinB is more efficient than that of DmmA by a factor of approximately 13.5. However, unlike DmmA, LinB is irreversibly inactivated via a slow off-pathway reaction with the substrate (*k*_inact_ = 0.00423 ± 0.00024 s^–1^). The FitSpace confidence contour analysis^36^ of the correctness of the obtained kinetic constants is visualized in Figures S38 and S39.

**Table 1:**
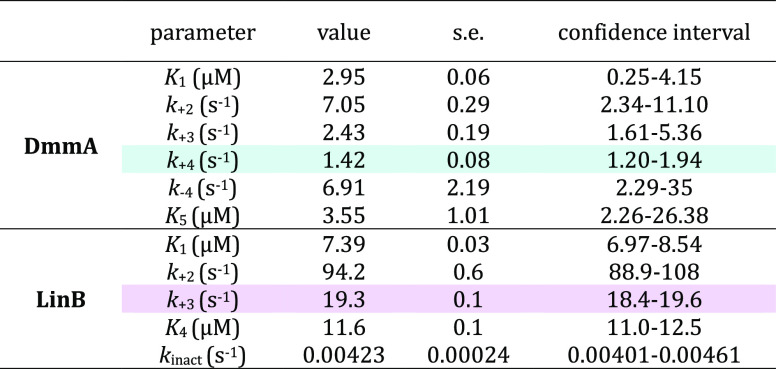
Kinetic Constants of *E*-**3c** Conversion by DmmA and LinB at 37 °C, pH 8.6^a^

a The rate-limiting steps are highlighted in teal and purple. Kinetic and equilibrium constants refer to the mechanism shown in Figure 4: *K*_1_ – equilibrium dissociation constant for enzyme-substrate complex; *k*_+2_ – nucleophilic substitution; *k*_+3_ – hydrolysis; *k*_+4_/*k*_–4_ – isomerization of enzyme-product complex; *K*_4_/*K*_5_ – equilibrium dissociation constant for enzyme-product complex; *k*_inact_ – the rate of enzyme inactivation. The standard error (s.e.) was calculated from the covariance matrix. In addition to s.e. values, a more rigorous analysis of the variation of the kinetic parameters was accomplished by confidence contour analysis

The data shown here clearly indicate that probe *E*-**3c** can be used to quantify individual steps in the reaction mechanism of HLDs precisely. Such analyses can contribute to a better understanding of the principles of enzyme function and play an important role in refining *in silico* methods used for the rational design of enzymes. This is primarily facilitated by the ability to integrate traditional pre-steady-state measurements with structural insights derived from time-resolved femtosecond crystallography.

### Analysis of Slow Irreversible Inhibition by *Z*-**3c**

To confirm the substrate-mediated enzyme inhibition indicated by global kinetic analysis and to determine which isomer of **3c** is responsible for this effect, the enzymes were preincubated with either *E*-**3c** or *Z*-**3c.** This was followed by adding the active substrate form, *E*-**3c**, after which tryptophan fluorescence was measured to follow the reaction (Figure S40). Preincubation with *Z*-**3c** had a negative effect on the kinetics and most substantially deviated from the fresh, non-preincubated enzyme trace. A minor kinetics impairment was also detected after the preincubation with *E-***3c**, but the observed deviation likely originates from a small fraction of *Z*-**3c** present in the sample. Importantly, the inhibition effect of *Z*-**3c** was significantly more prominent in the case of LinB compared to only a minor deviation observed for DmmA. Such an outcome is consistent with the kinetic mechanism shown here, including significant slow irreversible inhibition of LinB. For this purpose, DmmA was selected as a better candidate for the follow-up photoisomerization experiment.

### Effect of Irradiation on Enzyme Stability

To verify that the enzymes survive photoisomerization conditions, we performed steady-state experiments with DmmA and LinB solutions irradiated with a white laser (400–700 nm, Figure S24). After irradiation, the enzymes were titrated to *E*-**3c**, and their activities were monitored using ITC (Figure S41). The enzymes exhibited only a minimal activity loss, making them suitable for photoisomerization experiments.

### Laser-Triggered Photoswitching

**Figure 5:**
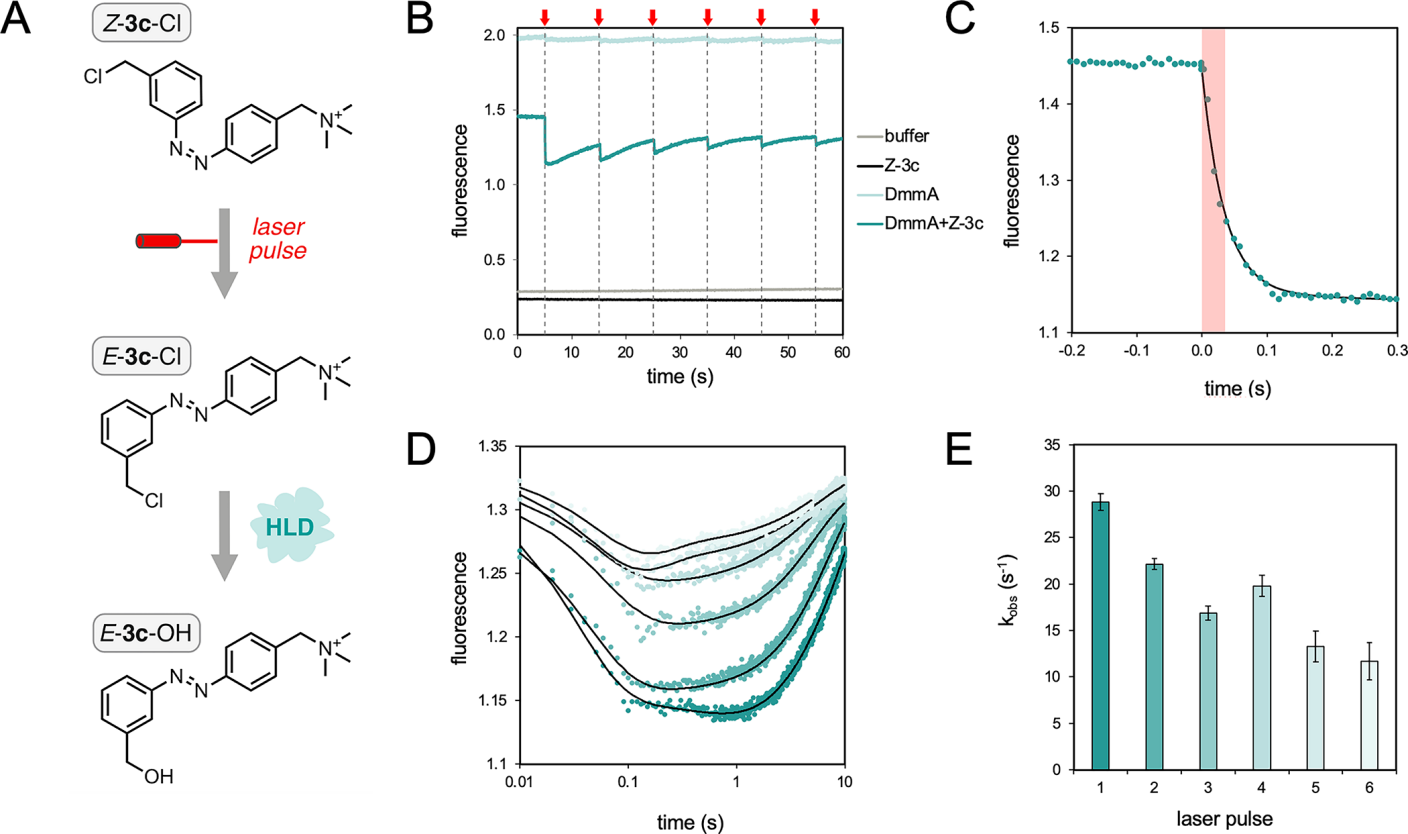
Laser-triggered *Z*-to-*E* photoisomerization and the subsequent conversion of *Z*-**3c** in the presence of DmmA. (A) DmmA (8 μM) was incubated with 220 μM substrate *Z*-**3c**-Cl and repeatedly illuminated by a laser pulse (400–700 nm, Figure S24), leading to isomerization to *E*-**3c**-Cl and subsequent enzymatic conversion to *E*-**3c**–OH. (B) Precise conversion fluorescence curves (teal) can be observed upon each laser pulse (indicated by red arrows and dashed lines), directly capturing the successful *Z*-to-*E* isomer switch in the reaction mixture and immediate substrate conversion by the enzyme. Negative controls (buffer only, *Z*-**3c**-Cl only, DmmA only) show no significant changes in the fluorescence signal, validating the selectivity of the flash-induced substrate conversion and applicability of the developed photoswitchable substrate **3c**. (C) The initial phase of the reaction was monitored through fluorescence intensity (excitation at 275 nm, emission exceeding 320 nm) following the first laser pulse (red zone). (D) The overall course of the reaction after each laser pulse fits a triple exponential function (black line). The time axis is represented on a logarithmic scale. (E) The observed rate of the first reaction phase (initial fluorescence decay) was assessed after individual pulses. The experiment was performed at room temperature in 100 mM glycine buffer pH 8.6.

We examined whether *Z*-to-*E* photoisomerization initiated by a short laser pulse leads to the formation of *E*-**3c**, which then undergoes the follow-up *in situ* conversion in the presence of DmmA (Figure 5). Indeed, a typical tryptophan fluorescence conversion curve could be repeatedly recorded for the mixture of DmmA and *Z*-**3c** immediately following a laser pulse (Figure 5B). The overall course of the reaction after the laser pulses fit a triple exponential function (Figure 5C,D), offering comprehensive insights primarily into the early stages of the reaction. Specifically, the initial segment of the data (within the first 0.3 s) is delineated with precision, eliminating potential artifacts from physically disruptive rapid mixing, thereby affording a robust estimation of the observed rates (Figure 5E). Integration of the laser-induced data into the previously established global model (illustrated in Figure 4) facilitated the derivation of the rates of enzyme–substrate complex association (1.67 ± 0.06 s^–1^ μM^–1^) and dissociation (3.18 ± 1.2 s^–1^). These parameters were previously poorly defined in rapid mixing data sets (Table 1). Furthermore, a confidence contour analysis of the variance corroborated that the additional kinetic parameters of the model are constrained by the expanded experimental data set (Figure S42). This observation confirmed the overall rational concept and full functionality of the developed photoswitchable substrate for HLDs. Thus, the herein-developed azobenzene probe is the first reported photoswitchable substrate for this model enzyme family. The laser-induced isomerization opens new possibilities for recording transient states of enzyme reactions, obtaining mechanistic data, and, above all, the possibility to combine conventional kinetic analysis with time-resolved femtosecond crystallography experiments, offering new insights into enzyme structure–function relationships.

## 4 Conclusions

In this work, we employed a rational multidisciplinary approach to identify a photoswitchable substrate for selected model HLD biocatalysts. The general methodology, encompassing computational analysis, chemical synthesis, physicochemical property screening, and kinetic analysis, can be applied to a wide range of enzyme families. The photoswitchable azobenzene-based substrate **3c** reported here provides a powerful tool for a detailed kinetic analysis of HLDs. Although this model enzyme family has been studied extensively over the past few decades,^40,42−45^ pre-steady-state kinetics measured using this probe further contributed to our understanding of its catalytic cycle. The kinetic mechanism is similar to previous kinetic studies;^24,40,46^ however, substrate **3c** revealed several interesting findings. For example, it provided an observation of the enzyme dynamics necessary for the completion of the catalytic cycle and made it possible to observe a slow, irreversible inhibition, which had not yet been observed for this enzyme family. Laser-induced kinetic analysis allowed more accurate derivation of substrate association and dissociation rates previously poorly defined in rapid mixing data sets. Interestingly, substrate **3c** exhibits an order of magnitude stronger binding than common small HLD substrates, similar to previously reported fluorogenic substrates.^39^ Although the biological role of HLDs remains unknown, these observations suggest that, despite the enzymes’ broad substrate specificity for small halogenated molecules, more intricate bulk substrates demonstrate superior structural compatibility. The activity of these larger substrates with the LinB variant is particularly intriguing, considering the enzyme’s crystal structure, which reveals a very narrow tunnel. Consequently, high catalytic efficiencies rely on the enzyme’s abnormal conformational flexibility. Therefore, the substrate provides an opportunity to investigate the less-explored effects of conformational dynamics on the enzyme’s catalytic efficiency more thoroughly. As shown by other photoswitchable substrates,^47−49^ compound **3c** could further be used in time-resolved serial femtosecond crystallography, offering insights into the relationships between enzyme structure and function. Such information can contribute to the advanced understanding of structure–function relationships of this model enzyme family and provide essential data for developing new mechanism-based concepts in protein engineering and the related development of modern *in silico* methods for rational protein design.^21,22,50,51^

## Supporting Information Available

Supplementary methods: synthesis, molecular docking, and photostationary-state calculations; supplementary tables; supplementary figures; and references (PDF)
