# Supplementary material for: Azobenzene-Based Photoswitchable Substrates for Advanced Mechanistic Studies of Model Haloalkane Dehalogenase Enzyme Family
Source: ACS Catal. 2024 Jul 22;14(15):11635–45. doi: 10.1021/acscatal.4c03503 (PMC11301625; doi:10.1021/acscatal.4c03503)
Supplement: Supplementary file 1 — cs4c03503_si_001.pdf [file cs4c03503_si_001.pdf]

SUPPORTING INFORMATION

Azobenzene-based Photoswitchable Substrates for Advanced Mechanistic Studies of Model Haloalkane Dehalogenase Enzyme Family

Michaela Slanska,[1] Lenka Stackova,[2,3] Sergio M. Marques,[1,4] Peter Stacko,[2,3] Marek Martínek,[2,3] Luboš Jílek,[2,3] Martin Toul,[1,4] Jiri Damborsky,[1,4] David Bednar,[1,4,*] Petr Klán,[2,3,*] Zbynek Prokop[1,4,*]

[1] Loschmidt Laboratories, Department of Experimental Biology and RECETOX, Faculty of Science, Masaryk University, Brno, Czech Republic

[2] RECETOX, Faculty of Science, Masaryk University, Brno, Czech Republic

[3] Department of Chemistry, Faculty of Science, Masaryk University, Brno, Czech Republic

[4] International Clinical Research Centre, St. Ann's Hospital, Brno, Czech Republic

*To whom the correspondence should be addressed: Zbynek Prokop (zbynek@chemi.muni.cz), Petr Klán (klan@sci.muni.cz), and David Bednar(222755@mail.muni.cz)

Content

1. Supplementary Methods 2
   1.1. Synthesis 2
   1.2. Molecular docking 6
   1.3. Photostationary-state calculations 7
2. Supplementary Tables 8
3. Supplementary Figures 11
4. References 38

1. Supplementary Methods

1.1. Synthesis

Reagents and solvents of the highest purity available were used as purchased, or they were purified/dried when necessary. Phosphate buffer saline (PBS) was prepared by diluting commercially available PBS tablets in a volumetric flask with demineralized water (pH = 7.4, I = 0.1 mol dm^{-3}). All synthetic steps were performed under an ambient atmosphere unless stated otherwise.

NMR spectra were recorded on 300 or 500 MHz spectrometers in d_6-DMSO, $CDCl_3$ or 100 mM d_5-glycine in D_2O buffer, pH = 8.6 (adjusted with a diluted 40% solution of NaOD in D_2O). The signals in ^1H and ^{13}C NMR spectra were referenced[1] to the residual peak of a (major) solvent. d_6-DMSO and $CDCl_3$ were kept over a high-temperature-dried 3 Å molecular sieve (8−12 mesh).

UV/VIS spectra were obtained with 1.0 cm quartz cells. Fluorescence was measured on an automated luminescence spectrometer in 1.0 cm quartz fluorescence cuvettes at 23 ± 2 °C using 90-degree mode; the sample concentration was set to keep the absorbance below 0.1 at λ_{max}; emission spectra were normalized and corrected using standard correction files. Molar absorption coefficient of **3c** was determined from the absorption spectra obtained from the measurement of three independently prepared samples, each divided into seven samples and diluted to keep the absorbance in the range of 0.1-1.5, and the absorbances were fit using linear functions.

Exact masses were obtained using a time-of-flight mass spectrometer with electrospray ionization or atmospheric-pressure chemical ionization in a positive mode. Melting points were obtained using an automated melting point apparatus.

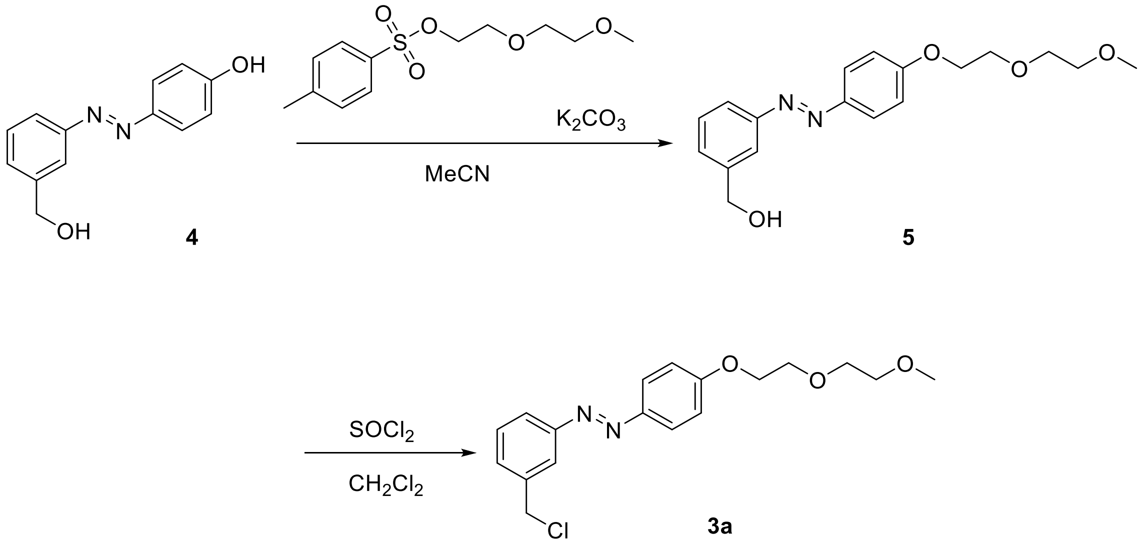

(*E*)-4-((3-(Hydroxymethyl)phenyl)diazenyl)phenol (4).

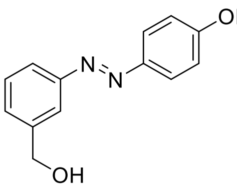

3-Aminobenzyl alcohol (1.00 g, 8.12 mmol) was dissolved in a mixture of water and acetone (1:1, 30 mL). Aq. HCl (36%, 2.3 mL, 73 mmol) was added in one portion, and the mixture was cooled down to 0 °C. A solution of sodium nitrite (0.6 g, 8.9 mmol) in water (15 mL) was added dropwise, and the mixture was stirred at 0 °C for 30 min. The formed diazonium salt was then added to a solution of Na_2CO_3 (1.5 g, 13.8 mmol), NaOH (0.36 g, 8.9 mmol), and phenol (0.84 g, 8.9 mmol) in water (20 mL) at 0 °C. The reaction mixture was stirred at 0 °C for 1 h. Afterward, the mixture was extracted with dichloromethane (2 × 50 mL). The solvent was evaporated at reduced pressure, and the crude product was purified by column chromatography (silica gel, hexane/EtOAc, 1:1) to give the pure product as an orange solid (1.5 g, 81%). Mp. 117.7–118.5 °C. ^{1}H NMR (300 MHz, d_6-DMSO): δ (ppm) 10.26 (s, 1H), 7.83-7.77 (m, 3H), 7.69 (d, 2H, J = 7.7 Hz), 7.54–7.41 (m, 2H), 6.95 (d, 2H, J = 8.9 Hz,), 5.31 (s, 1H), 4,61 (s, 2H). ^{13}C NMR (75 MHz, d_6-DMSO): δ (ppm) 160.9, 152.1, 145.2, 143.9, 128.9, 128.3, 124.7, 121.1, 119.2, 115.9, 62.5. HRMS (APCI+): calcd for $C_{13}H_{13}N_2O_2$ (M+H$^+$) 229.0972, found 229.0968.

(*E*)-(3-((4-(2-(2-Methoxyethoxy)ethoxy)phenyl)diazenyl)phenyl)methanol (5).

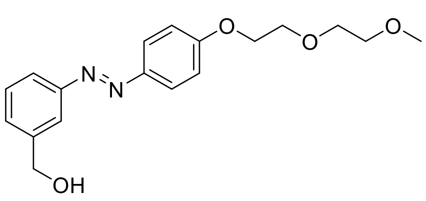

Azobenzene **2** (0.50 g, 2.2 mmol) was dissolved in acetonitrile (30 mL), 2-(2-methoxyethoxy)ethyl-4-methylbenzenesulfonate (0.72 g, 2.6 mmol), and K_2CO_3 (0.6 g, 4.4 mmol) were added, and the mixture was refluxed for 12 h. It was then cooled to room temperature, water (40 mL) was added, and the mixture was extracted with CH_2Cl_2 (2 × 40 mL), dried with $MgSO_4$, and the solvents were evaporated at reduced pressure. The crude product was purified by column chromatography (silica gel, hexane/EtOAc, 1:1, then switched to 1:2) to give the pure product as an orange oil (0.6 g, 83%). ^{1}H NMR (500 MHz, d_6-DMSO): δ (ppm) 7.89 (d, 2H, J = 9.0 Hz), 7.81 (s, 1H), 7.73 (d, 1H, J = 7.8 Hz), 7.55–7.50 (m, 1H), 7.48–7.44 (m, 1H), 7.15 (d, 2H, J = 9.1 Hz), 5.33 (t, 1H, J = 5.8 Hz), 4.61 (d, 2H, J = 5.5 Hz), 4.25–4.18 (m, 2H), 3.82–3.75 (m, 2H), 3.65–3.58 (m, 2H), 3.50–3.45 (m, 2H), 3.26 (s, 3H). ^{13}C NMR (125 MHz, d_6-DMSO): δ (ppm) 161.2, 152.0, 146.2, 144.0, 129.0, 128.6, 124.4, 121.3, 119.3, 115.1, 71.2, 69.7, 68.7, 67.6, 62.5, 58.0. HRMS (APCI+): calcd for $C_{18}H_{23}N_2O_4$ (M+H$^+$) 331.1652, found 331.1647.

(*E*)-(1-(3-(Chloromethyl)phenyl)-2-(4-(2-(2-methoxyethoxy)ethoxy)phenyl)diazene (3a).

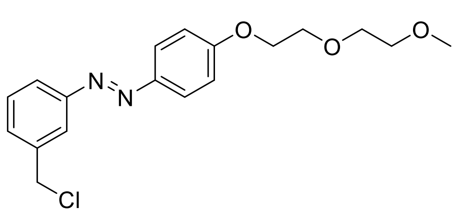

$SOCl_2$ (127 µL, 1.75 mmol) was added dropwise to a solution of **3** (385 mg, 1.17 mmol) in CH_2Cl_2 (10 mL), and the mixture was stirred at room temperature for 12 h. The reaction was quenched by the addition of sat. aq. $NaHCO_3$ (15 mL), and the mixture was extracted with CH_2Cl_2 (2 × 10 mL). The solvent was evaporated at reduced pressure, and the pure product was obtained without additional purification as an orange oil (380 mg, 94%). ^{1}H NMR (300 MHz, d_6-DMSO): δ (ppm) 7.93–7.87 (m, 3H), 7.85–7.79 (m, 1H), 7.61–7.57 (m, 2H), 7.15 (d, 2H, J = 9.0 Hz), 4.89 (s, 2H), 4.24–4.18 (m, 2H), 3.81–3.75 (m, 2H), 3.63–3.58 (m, 2H), 3.49–3.45 (m, 2H), 3.25 (s, 3H). ^{13}C NMR (75 MHz, d_6-DMSO): δ (ppm) 161.4, 152.1, 146.1, 139.0, 130.9, 129.7, 124.6, 122.7, 121.8, 115.1, 71.2, 69.7, 68.7, 67.6, 58.0, 45.6. HRMS (APCI+): calcd for $C_{18}H_{22}ClN_2O_3$ (M+H$^+$) 349.1313, found 349.1307.

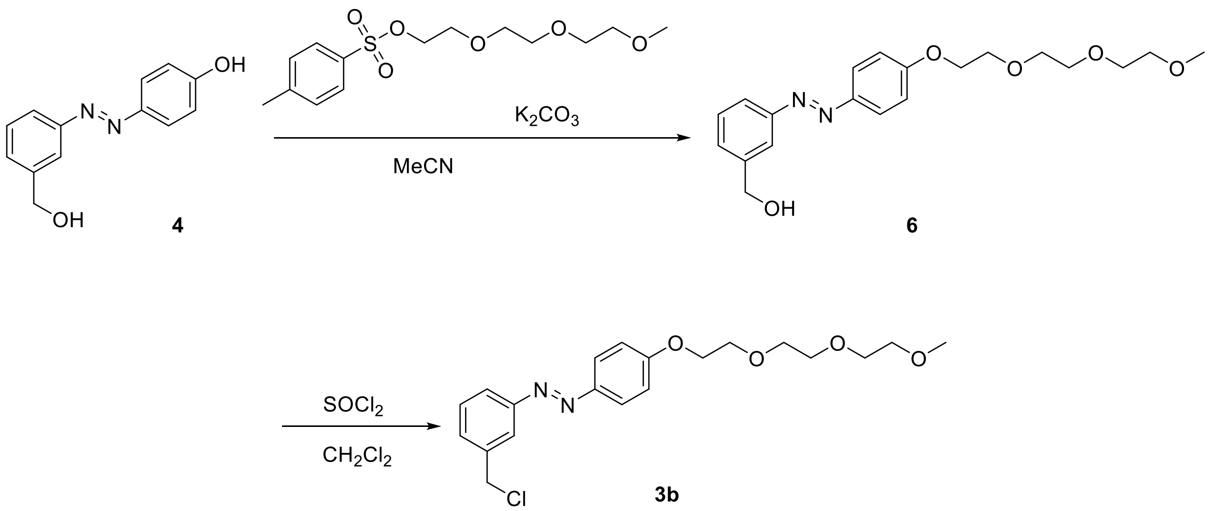

(*E*)-(3-((4-(2-(2-(2-Methoxyethoxy)ethoxy)ethoxy)phenyl)diazenyl)phenyl)methanol (6).

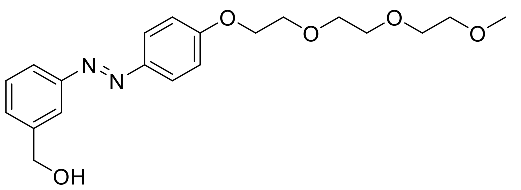

Azobenzene **2** (0.50 g, 2.2 mmol) was dissolved in acetonitrile (30 mL), 2-(2-(2-methoxyethoxy)ethoxy)ethyl-4-methylbenzenesulfonate (0.84 g, 2.6 mmol) and K_2CO_3 (0.60 g, 4.4 mmol) were added, and the mixture was refluxed for 12 h. The mixture was cooled to room temperature, water (40 mL) was added, and the mixture was extracted with CH_2Cl_2 (2 × 40 mL), dried with $MgSO_4$, and the solvents were evaporated at reduced pressure. The crude product was purified by column chromatography (silica gel, hexane/EtOAc, 1:1, then switched to 1:2) to give the pure product as an orange oil (0.70 g, 85%). ^1H NMR (500 MHz, d_6-DMSO): δ (ppm) 7.89 (d, 2H, J = 9.1 Hz), 7.82 (s, 1H), 7.73 (d, 1H, J = 7.8 Hz), 7.55 -7.50 (m, 1H), 7.49–7.44 (m, 1H), 7.14 (d, 2H, J = 9.0 Hz), 5.33 (t, 1H, J = 5.6 Hz), 4.62 (d, 2H, J = 5.6 Hz), 4.24–4.19 (m, 2H), 3.82–3.76 (m, 2H), 3.64–3.58 (m, 2H), 3.58–3.50 (m, 4H), 3.46–3.40 (m, 2H), 3.24 (s, 3H). ^{13}C NMR (125 MHz, d_6-DMSO): δ (ppm) 161.2, 152.0, 146.2, 144.0, 129.0, 128.6, 124.4, 121.3, 119.3, 115.1, 71.2, 69.9, 69.7, 69.6, 68.8, 67.6, 62.5, 58.0. HRMS (APCI+): calcd for $C_{20}H_{27}N_2O_5$ (M+H$^+$) 375.1914, found 375.1914.

(*E*)-(1-(3-(Chloromethyl)phenyl)-2-(4-(2-(2-(2-methoxyethoxy)ethoxy)ethoxy)phenyl)diazene (3b).

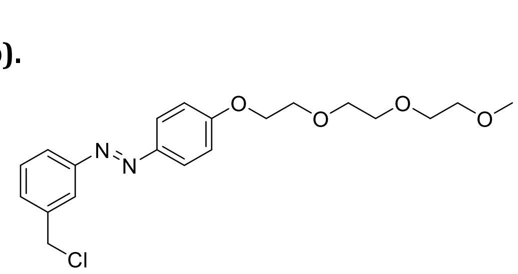

$SOCl_2$ (0.15 mL, 2.1 mmol) was added dropwise to a solution of **5** (510 mg, 1.17 mmol) in CH_2Cl_2 (10 mL), and the mixture was stirred at room temperature for 12 h. The reaction was quenched by the addition of sat. aq. $NaHCO_3$ (15 mL), and the mixture was extracted with CH_2Cl_2 (2 × 20 mL). The solvent was evaporated at reduced pressure, and the pure product was obtained without additional purification as an orange oil (540 mg, 94%). ^1H NMR (300 MHz, d_6-DMSO): δ (ppm) 7.93–7.87 (m, 3H), 7.85–7.78 (m, 1H), 7.61–7.56 (m, 2H), 7.15 (d, 2H, J = 9.1 Hz), 4.89 (s, 2H), 4.24–4.18 (m, 2H), 3.81–3.75 (m, 2H), 3.63–3.58 (m, 2H), 3.57–3.49 (m, 2H), 3.45–3.40 (m, 2H), 3.23 (s, 3H). ^{13}C NMR (75 MHz, d_6-DMSO): δ (ppm) 161.4, 152.1, 146.1, 139.0, 130.9, 129.7, 124.6, 122.6, 121.8, 115.1, 71.2, 69.9, 69.7, 69.6, 68.8, 67.6, 58.0, 45.6. HRMS (APCI+): calcd for $C_{20}H_{26}ClN_2O_4$ (M+H$^+$) 393.1576, found 393.1574.

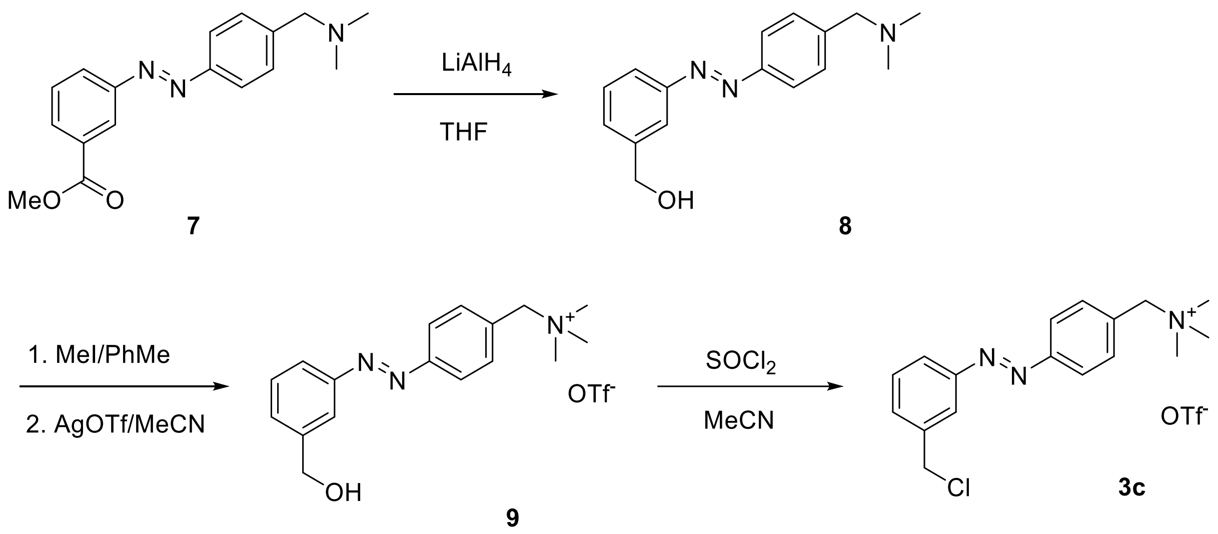

Methyl (*E*)-3-((4-((dimethylamino)methyl)phenyl)diazenyl)benzoate (7).

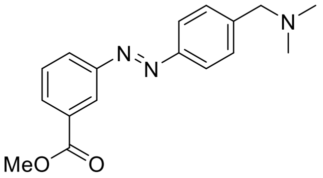

A mixture of 4-[(dimethylamino)methyl]aniline[2] (1.30 g, 8.6 mmol) and methyl 3-nitrosobenzoate[3] (1.19 g, 7.2 mmol) in acetic acid (20 mL) was stirred overnight at room temperature. The solvent was evaporated at reduced pressure, water (30 mL) was added to the residue, and the mixture was neutralized with aq. K_2CO_3 (1M, to pH = 7). The mixture was extracted with CH_2Cl_2 (3 × 40 mL). The combined organic layers were washed with brine (50 mL), dried with $MgSO_4$, and the solvents were evaporated at reduced pressure. The residue was purified by column chromatography (silica gel, CH_2Cl_2/MeOH, 20:1) to give the product as a red solid (1.21 g, 57%). Mp 54.8–55.4 °C. ^{1}H NMR (500 MHz, $CDCl_3$): δ (ppm) 8.58 (dd, 1H, J_1 = 1.8 Hz, J_2 = 1.8 Hz), 8.17 (ddd, 1H, J_1 = 7.9 Hz, J_2 = 1.9 Hz, J_3 = 1.9 Hz), 8.12 (ddd, 1H, J_1 = 7.9 Hz, J_2 = 1.9 Hz, J_3 = 1.9 Hz), 7.93 (d, 2H, J = 8.4 Hz), 7.61 (dd, 1H, J_1 = 7.8 Hz, J_2 = 7.8 Hz), 7.50 (d, 2H, J = 8.5 Hz), 4.00 (s, 3H), 3.53 (s, 2H), 2.30 (s, 6H). ^{13}C NMR (125 MHz, $CDCl_3$): δ (ppm) 166.8, 152.9, 151.9, 143.1, 131.8, 131.6, 129.9, 129.4, 127.1, 124.2, 123.2, 64.3, 52.5, 45.7. HRMS (APCI+): calcd for $C_{17}H_{20}N_3O_2^+$ (M+H$^+$) 298.1556, found 298.1548.

(*E*)-(3-((4-((Dimethylamino)methyl)phenyl)diazenyl)phenyl)methanol (8).

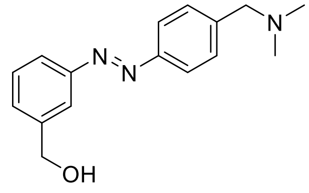

A solution of **7** (1.00 g, 3.4 mmol) in dry THF (30 mL) was treated with solid $LiAlH_4$ (255 mg, 6.7 mmol) in portions at 0 °C. The resulting mixture was stirred at room temperature for 1 h and quenched with sat. aq. NH_4Cl (40 mL). The mixture was extracted with CH_2Cl_2 (3 × 40 mL). The combined organic layers were washed with brine (50 mL), dried with $MgSO_4$, and the solvents were evaporated at reduced pressure. The residue was purified by column chromatography (silica gel, CH_2Cl_2/MeOH, 20:1) to give the product **8** as a red oil (842 mg, 93%). ^{1}H NMR (500 MHz, $CDCl_3$): δ (ppm) 7.93–7.89 (m, 4H), 7.54–7.47 (m, 4H), 4.83 (s, 2H), 3.52 (s, 2H), 2.30 (s, 6H). ^{13}C NMR (125 MHz, $CDCl_3$): δ (ppm) 153.1, 152.1, 142.5, 142.3, 123.0, 129.5, 129.4, 123.0, 122.7, 120.8, 65.1, 64.2, 45.6. HRMS (APCI+): calcd for $C_{16}H_{20}N_3O^+$ (M + H$^+$) 270.1601, found 270.1599.

(*E*)-1-(4-((3-(Hydroxymethyl)phenyl)diazenyl)phenyl)-*N*,*N*,*N*-trimethylmethanaminium trifluoromethanesulfonate (9).

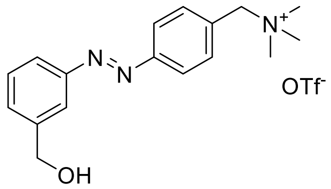

A solution of **8** (650 mg, 2.4 mmol) in toluene (30 mL) was treated with MeI (514 mg, 225 µL, 3.6 mmol) at room temperature. The reaction mixture was stirred at room temperature overnight, filtered through a glass filter, and the solid was washed with Et_2O (2 × 20 mL). The solid (821 mg, 2.0 mmol) was redissolved in MeCN (30 mL), and a solution of AgOTf (513 mg, 2.0 mmol) in MeCN (3 mL) was added dropwise at room temperature. After stirring for 1 h, the formed AgI was filtered off and washed with Et_2O (2 × 10 mL). The combined liquids were evaporated at reduced pressure to give the product **9** as a red oil (864 mg, 83%). ^{1}H NMR (500 MHz, d_6-DMSO): δ (ppm) 8.01 (d, 2H, J = 8.2 Hz), 7.88 (s, 1H), 7.82–7.75 (m, 3H), 7.61–7.53 (m, 2H), 5.37 (t, 1H, J = 5.5 Hz), 4.64–4.62 (m, 4H), 3.08 (s, 9H). ^{13}C NMR (125 MHz, d_6-DMSO): δ (ppm) 152.7, 151.9, 144.3, 134.0, 131.2, 129.9, 129.2, 122.7, 121.8, 119.7, 67.3, 62.3, 51.9. HRMS (APCI+): calcd for $C_{17}H_{22}N_3O^+$ (M – OTf^-) 284.1757 found 284.1755.

(*E*)-1-(4-((3-(Chloromethyl)phenyl)diazenyl)phenyl)-*N*,*N*,*N*-trimethylmethanaminium trifluoromethanesulfonate (3c).

$SOCl_2$ (494 mg, 301 µL, 4.2 mmol) was added dropwise to a solution of **9** (602 mg, 1.4 mmol) in dry acetonitrile (30 mL) at room temperature. The reaction mixture was stirred at room temperature overnight. The volatiles were evaporated at reduced pressure. Toluene (30 mL) was added to the residue, and the volatiles were again evaporated at reduced pressure. The residue was purified by column chromatography (neutral aluminum oxide, CH_2Cl_2/MeOH, 20:1) to give the product **10** as a thick orange oil (540 mg, 86%). ^{1}H NMR (500 MHz, d_6-DMSO): δ (ppm) 8.03–7.98 (m, 3H), 7.90 (m, 1H), 7.79 (d, 2H, J = 8.4 Hz), 7.70–7.62 (m, 2H), 5.37 (t, 1H, J = 5.5 Hz), 4.92 (s, 2H), 4.66 (s, 2H), 3.09 (s, 9H). ^{13}C NMR (125 MHz, $CDCl_3$): δ (ppm) 152.6, 152.0, 139.3, 134.1, 132.2, 131.5, 123.0, 123.1, 122.8, 122.3, 67.2, 51.9, 45.4. HRMS (ESI+): calcd for $C_{17}H_{21}N_3Cl^+$ (M – OTf^-) 302.1419 found 302.1416. UV/VIS (glycine buffer 100 mM, pH = 8.6, $c = 4.2 \times 10^{-3}$ M): λ_{max} (ε) = 320 nm (25000 L mol^{-1} cm^{-1}).

1.2. Molecular docking

Three-dimensional structures of the *E* and *Z*-isomers of the azobenzene ligands were constructed in Avogadro[4] and minimized using the UFF force field[5] and the steepest descent algorithm. Three-dimensional structures of the receptors were obtained from the RCSB Protein Data Bank[6]: DbjA (PDB ID: 3A2M), DmmA (3U1T), DhaA (4E46) and LinB (1MJ5). Only the first chain of each structure was kept, and all the structures were aligned with PyMOL 1.7.4[7]. The water molecules, ions, and co-crystallization molecules were removed. The double side chains of several residues were also removed, leaving only the conformations that were more frequently observed among all the structures. If the hydrogen atoms were missing, they were added with the *reduce* program of AmberTools 14[8] using a dynamic optimization of their positions.

The input files of the ligands and receptors in the pdb format were converted to the AutoDock Vina compatible format PDBQT using MGLTools[9]. The active site of the haloalkane dehalogenases was selected as the region of interest for the molecular docking performed by AutoDock Vina[10]. This region was represented by a box of 20 × 20 × 20 Å centered in the coordinates of the OD1

atom of the catalytic aspartate (D108 in LinB and D144 in DmmA). The exhaustiveness parameter was increased to 50 to improve the conformational search. Only the 12 best binding modes were saved.

The docking results were analysed using PyMOL 1.7.4. The identification of the reactive configurations for the S_N2 reaction was based on the distances and angles between the nucleophile and the substrate atoms, according to Hur *et al.*[11]: the distance between one of the nucleophile's carboxylic oxygen atoms (D144-OD atoms) and the halogen-bound carbon atom has to be ≤ 3.41 Å, and the angle formed by this oxygen, carbon and the halide atoms must be ≥ 157°. As reported before[12], we also required at least a weak H-bonding between the reactive chlorine atom and the halide-stabilizing residues, defined by the distance between the chlorine atom of the ligands and the indole polar hydrogen of W145, or the side chain HD hydrogen of N78 to be ≤ 3.0 Å (residue numeration of DmmA).

Access tunnel in HLDs

The access tunnels were calculated on the structures of HLDs (DbjA, DhaA, DmmA, and LinB), prepared, protonated, and aligned as described above, using the CAVER 3.03 plugin for PyMOL[13]. The carboxylic oxygen atoms of the catalytic aspartate were used to define the starting point for the tunnel calculation. The probe radius was set to 0.9 Å, the shell depth to 4 Å, the shell radius to 10 Å (larger than the default, in order to visualize the tunnels protruding far out into the solvent), and the clustering threshold to 3.5 Å.

1.3. Photostationary-state calculations

Quantum yields $\Phi_{E\text{-}Z}^{320}$ of the conversion of *E*-3c to *Z*-3c

The quantum yield $\Phi_{E\text{-}Z}^{320}$ of 0.13 was determined using an optical bench consisting of a high-pressure 450 W Xe arc lamp and a monochromator set to 320 nm for solutions (2.5 mL, $A_{320} \approx 1.8$) of *E*-**3c** in glycine buffer (100 mM, pH = 8.6). The light source was equilibrated for at least 45 min prior to the measurement, and the light stability was controlled during irradiation. The samples were irradiated in a 1.0 cm quartz cuvette. Ferrioxalate solutions were used as an actinometer.[14] The exact conversions were calculated by a linear fit of the absorbances at 320 nm, obtained from the absorption spectra recorded by UV/VIS spectrometer simultaneously during the irradiation between the initial (95% of *Z*-**3c**) and photostationary (25% of *Z*-**3c**) states, based on NMR analyses (**Figures S34** and **S35**). The reaction conversions were always kept below 15% to avoid the interference of photoproducts. All measurements were repeated at least three times.

$\varepsilon_{Z\text{-}\mathbf{3c}}^{320}$ calculation

Based on the equation for a photostationary state at 320 nm:

$$A_{\mathrm{PSS}}^{320} = \varepsilon_{E\text{-}\mathbf{3c}}^{320} \times c_{E\text{-}\mathbf{3c}} + \varepsilon_{Z\text{-}\mathbf{3c}}^{320} \times c_{Z\text{-}\mathbf{3c}}$$

and an approximation:

$c_0 = A_0^{320} / \varepsilon_{E\text{-}\mathbf{3c}}^{320}$

we write

$A_{\mathrm{PSS}}^{320} = \varepsilon_{E\text{-}\mathbf{3c}}^{320} \times c_0 \times w_{E\text{-}\mathbf{3c}}^{320} + \varepsilon_{Z\text{-}\mathbf{3c}}^{320} \times c_0 \times w_{Z\text{-}\mathbf{3c}}^{320}$

to show

$\varepsilon_{Z\text{-}\mathbf{3c}}^{320} = (A_{\mathrm{PSS}}^{320} - A_0^{320} \times w_{E\text{-}\mathbf{3c}}^{320}) / (A_0^{320} / \varepsilon_{E\text{-}\mathbf{3c}}^{320} \times w_{Z\text{-}\mathbf{3c}}^{320})$,

where the molar ratios $w_{E\text{-}\mathbf{3c}}^{320}$ and $w_{Z\text{-}\mathbf{3c}}^{320}$ are derived from [*E*-**3c**]/[*Z*-**3c**] = 0.25/0.75 estimated from an NMR analysis (Figure S36). We finally obtain $\varepsilon_{Z\text{-}\mathbf{3c}}^{320}$ = 1912 L mol^{-1} cm^{-1}.

$\Phi_{Z\text{-}E}^{320}$ of *Z*-3c to *E*-3c

From the equation given for photostationary state:

$[E\text{-}\mathbf{3c}]/[Z\text{-}\mathbf{3c}] = (\varepsilon_{Z\text{-}\mathbf{3c}}^{320} \times \Phi_{Z\text{-}E}^{320}) / (\varepsilon_{E\text{-}\mathbf{3c}}^{320} \times \Phi_{\mathrm{E\text{-}Z}}^{320})$

we calculated $\Phi_{Z\text{-}E}^{320}$ of 0.70.

2. Supplementary Tables

Table S1. Results of the docking calculations. For each ligand/conformation, the affinity of the best binding conformation ($\Delta G_{\mathrm{bind}}^{\mathrm{best}}$) is reported. In case it could bind in a reactive conformation (REAC), ranked position (#REAC) and the respective affinity ($\Delta G_{\mathrm{bind}}^{\mathrm{REAC}}$) are provided as well. The affinity difference between the reactive binding modes of *E* and *Z*-isomers ($\Delta\Delta G_{\mathrm{bind}}^{Z\text{-}E} = \Delta G_{\mathrm{bind}}^{Z} - \Delta G_{\mathrm{bind}}^{E}$) is presented. Negative ΔG_{bind} values are highlighted in green, and positive values in red.

**Enzyme**	**Ligand**	**$\Delta G_{\mathrm{bind}}^{\mathrm{best}}$**	**REAC***	**$\Delta G_{\mathrm{bind}}^{\mathrm{REAC}}$**	**#REAC**	**$\Delta\Delta G_{\mathrm{bind}}^{Z\text{-}E}$**
**DmmA**	*E*-1a	-6.2	yes--	-5.3	8	
	Z-1a	-6.9	yes	-6.9	1	< -1.6#
	E-1b	-6.3	yes	-5.2	8	
	Z-1b	-6.6	yes	-6.6	1	-1.4
	E-1c	-6.8	yes--	-6.1	2	
	Z-1c	-7.2	yes	-7.2	1	< -1.1#
	E-2	32.5	no	-		
	Z-2	-5.6	yes	-5.6	1	<< -5.6#
	E-3a	-6.5	yes	-6.5	1	
	Z-3a	-6.9	yes	-6.6	2	-0.1
	E-3b	-6.0	yes	-5.9	2	
	Z-3b	-6.6	yes-	-5.6	7	< 0.3#
	E-3c	-6.3	yes	-5.8	5	
	Z-3c	-7.0	yes-	-6.2	5	< -0.4#
	E-1a	2.6	yes	4	4	
	Z-1a	1.5	yes	1.5	1	-2.5

	E-1b	3.8	yes--	4.9	5	
	Z-1b	1.9	yes	1.9	1	< -3.0
	E-1c	2.1	yes--	4.1	5	
	Z-1c	1.6	yes	1.6	1	< -2.5#
**LinB**	*E*-2	46.1	no	-		
	Z-2	25.0	no	-		-
	E-3a	3.2	yes	3.2	1	
	Z-3a	-1.5	no	-		>> -3.2#
	E-3b	3.7	yes-	6.3	8	
	Z-3b	1.6	no	-		>> -6.3#
	E-3c	3.9	yes-	3.9	1	
	Z-3c	1.2	no	-		>> -3.9#
	E-1a	-6.0	no	-		
	Z-1a	-6.3	yes	-5.2	6	<< -5.2#
	E-1b	-5.7	yes	-3.4	4	
	Z-1b	-5.4	yes	-5.3	2	-1.9
	E-1c	-7.4	no	-		
	Z-1c	-6.3	yes	-6.3	1	<< -6.3#
**DbjA**	*E*-2	37.3	no	-		
	Z-2	4.9	no	-		-
	E-3a	-7.4	yes	-7.4	1	
	Z-3a	-6.8	yes-	-5.6	10	1.8#
	E-3b	-6.4	yes	-6.4	1	
	Z-3b	-6.3	yes-	-6.3	1	0.1#
	E-3c	-8.1	yes	-8.1	1	
	Z-3c	-6.7	yes-	-6.7	1	1.4#
	E-1a	0.2	yes	0.7	2	
	Z-1a	-4.0	yes	-2.6	2	-3.3
	E-1b	0.2	yes--	1.0	2	
	Z-1b	-2.5	no	-		>> -1.0#
	E-1c	-0.8	yes	-0.8	1	
	Z-1c	-4.6	yes	-3.8	3	-3.0
**DhaA**	*E*-2	41.2	no	-		
	Z-2	12.4	no	-		-
	E-3a	-0.1	no	-		
	Z-3a	-4.4	yes-	-1.7	5	<<-1.7#
	E-3b	-0.2	no	-		
	Z-3b	-4.8	no	-		-
	E-3c	1.1	yes-	2.5	5	
	Z-3c	-4.2	yes-	-3.2	2	-5.7#

*"yes-" in the reactive configuration means that it did not meet all criteria (e.g., reasonable O-C distances but unfavorable O-C-Cl angles). # $\Delta\Delta G_{bind}$ values with high uncertainty due to the poor quality of one of the reactive conformations. The relative values of the docking scores are color-coded as darker green for more favorable (ΔG_{bind} <0) and darker red for more unfavorable (ΔG_{bind} > 0).

Table S2. The scaling factors obtained by global fit of DmmA and LinB kinetic data. The standard error (s.e.) was calculated from the covariance matrix. Confidence intervals (lower and upper limits) of the parameters were obtained by confidence contour analysis for χ^2 threshold of 0.98.

	scaling factor	value	s.e.	confidence interval	
				lower limit	upper limit
DmmA	f	0.181	0.001	0.180	0.181
	t	0.072	0.001	0.072	0.074
	a	0.955	0.002	0.955	0.975
	b	0.894	0.003	0.793	0.919
	c	0.896	0.004	0.877	0.961
	d	1.9	0.2	1.39	3.18
LinB	f	0.214	0.001	0.213	0.214
	t	0.00024	0.00008	0.00001	0.00061
	a	0.100	0.001	0.083	0.111
	b	0.083	0.001	0.079	0.093
	c	0.202	0.002	0.192	0.216

3. Supplementary Figures

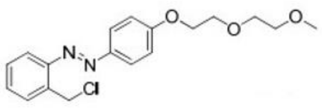

E-**1a**

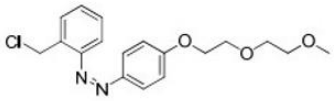

Z-**1a**

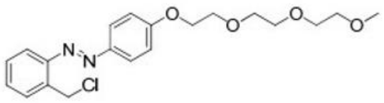

E-**1b**

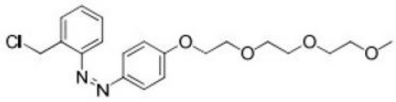

Z-**1b**

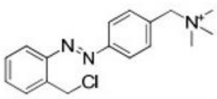

***E*-1c**

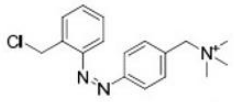

***Z*-1c**

E-**2**

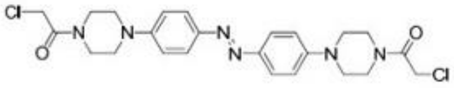

Z-**2**

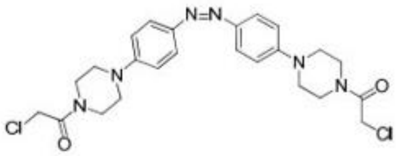

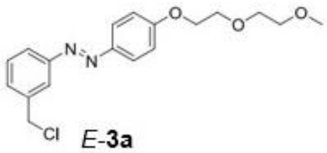

E-**3a**

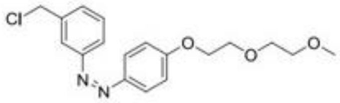

Z-**3a**

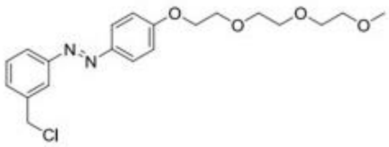

E-**3b**

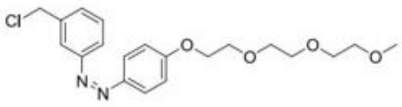

Z-**3b**

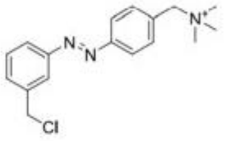

E-**3c**

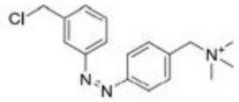

Z-**3c**

Figure S1: Designed photoswitchable compounds in *E* and *Z* conformations.

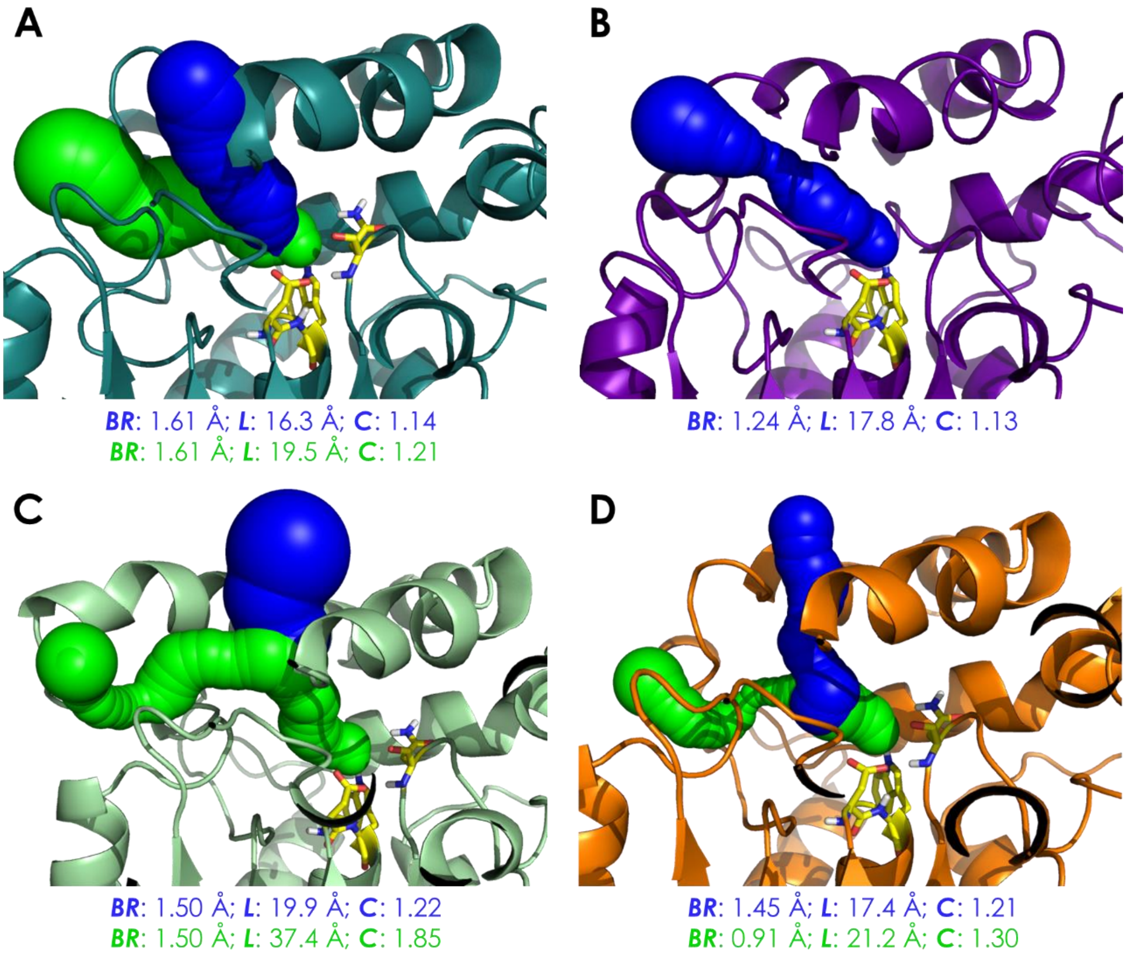

Figure S2: The main access tunnels in the different studied HLDs. A) DmmA (teal cartoon), B) LinB (purple), C) DbjA (light green), and D) DhaA (orange). The tunnels were calculated using CAVER 3[13] and are represented as spheres (the first-ranked as blue, and second-ranked as green); the respective geometric property are listed below each image, in the same colors as the tunnels: the bottleneck radius (*BR*), length (*L*), and curvature (*C*; curvature = tunnel length/distance in space between start and end points). The catalytic triads are represented as yellow sticks; the HLDs are all represented from the same viewpoint after being aligned.

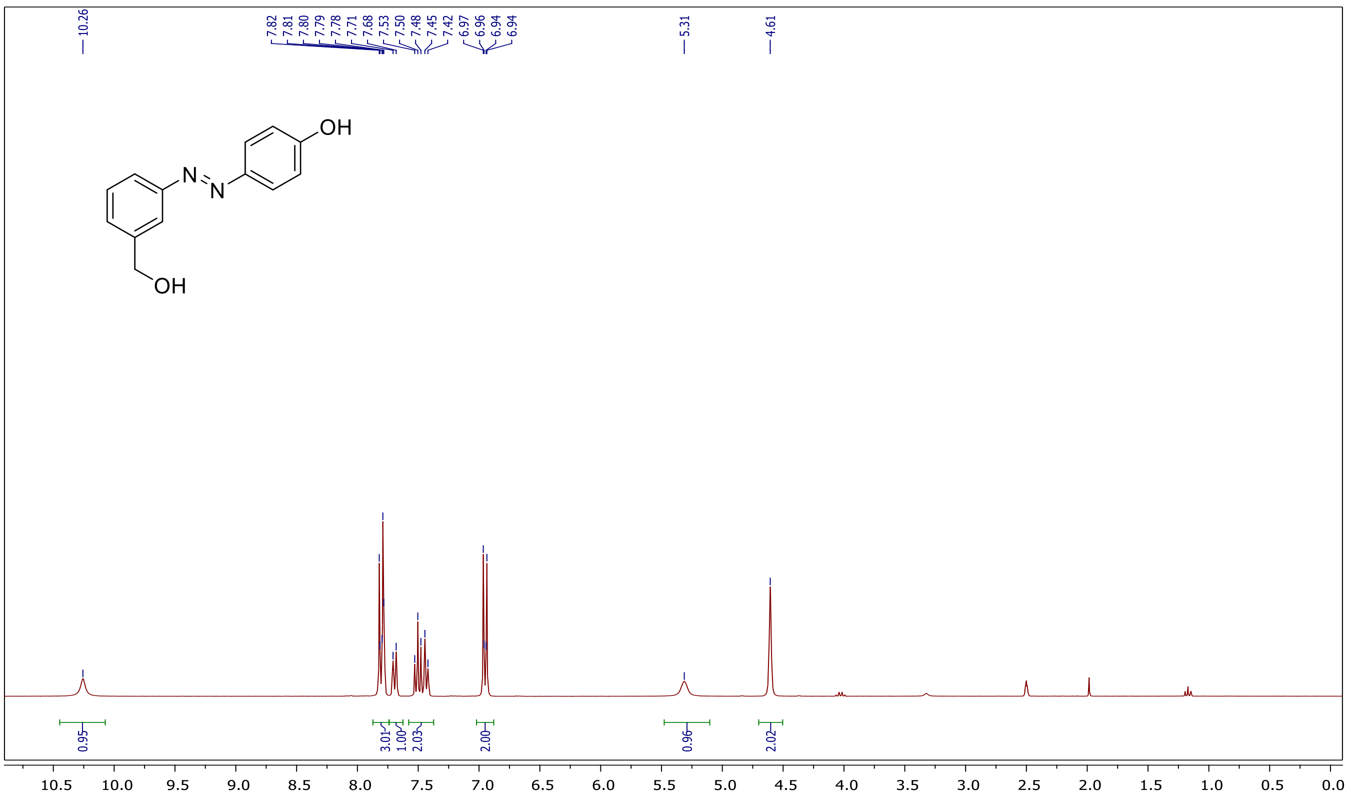

Figure S3: ^{1}H NMR (300 MHz, d_6-DMSO) spectrum of **4**.

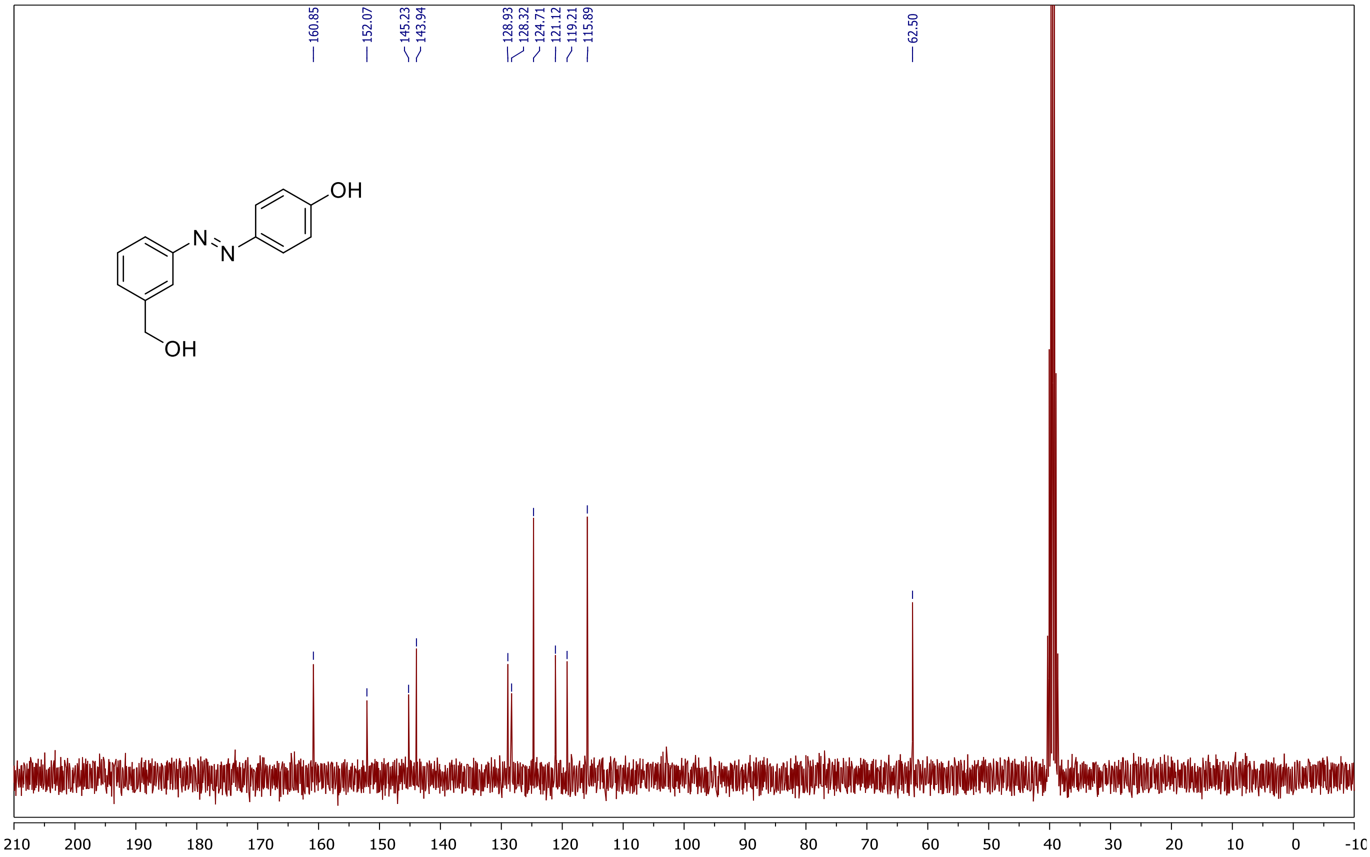

Figure S4: ^{13}C NMR (75 MHz, d_6-DMSO) spectrum of **4**.

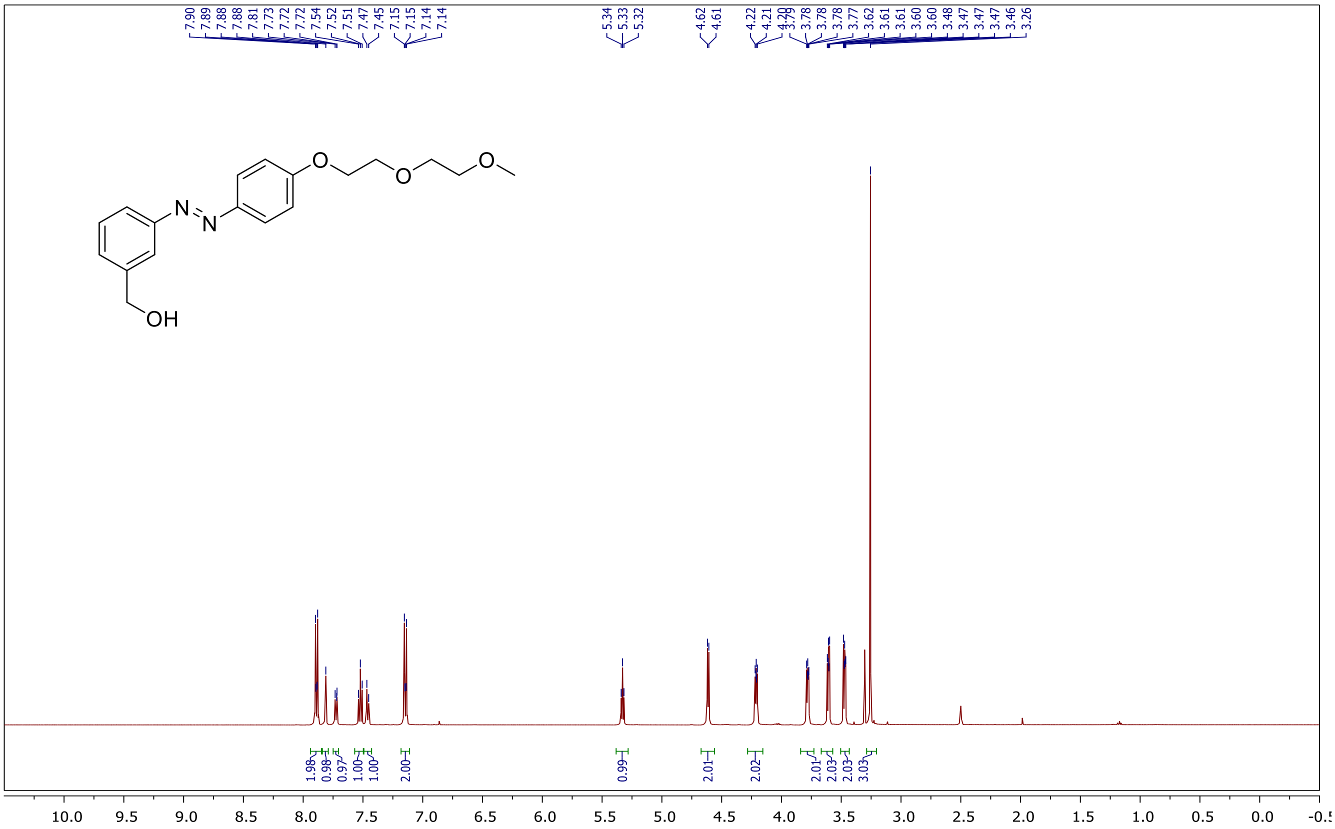

Figure S5: ^{1}H NMR (500 MHz, d_6-DMSO) spectrum of **5**.

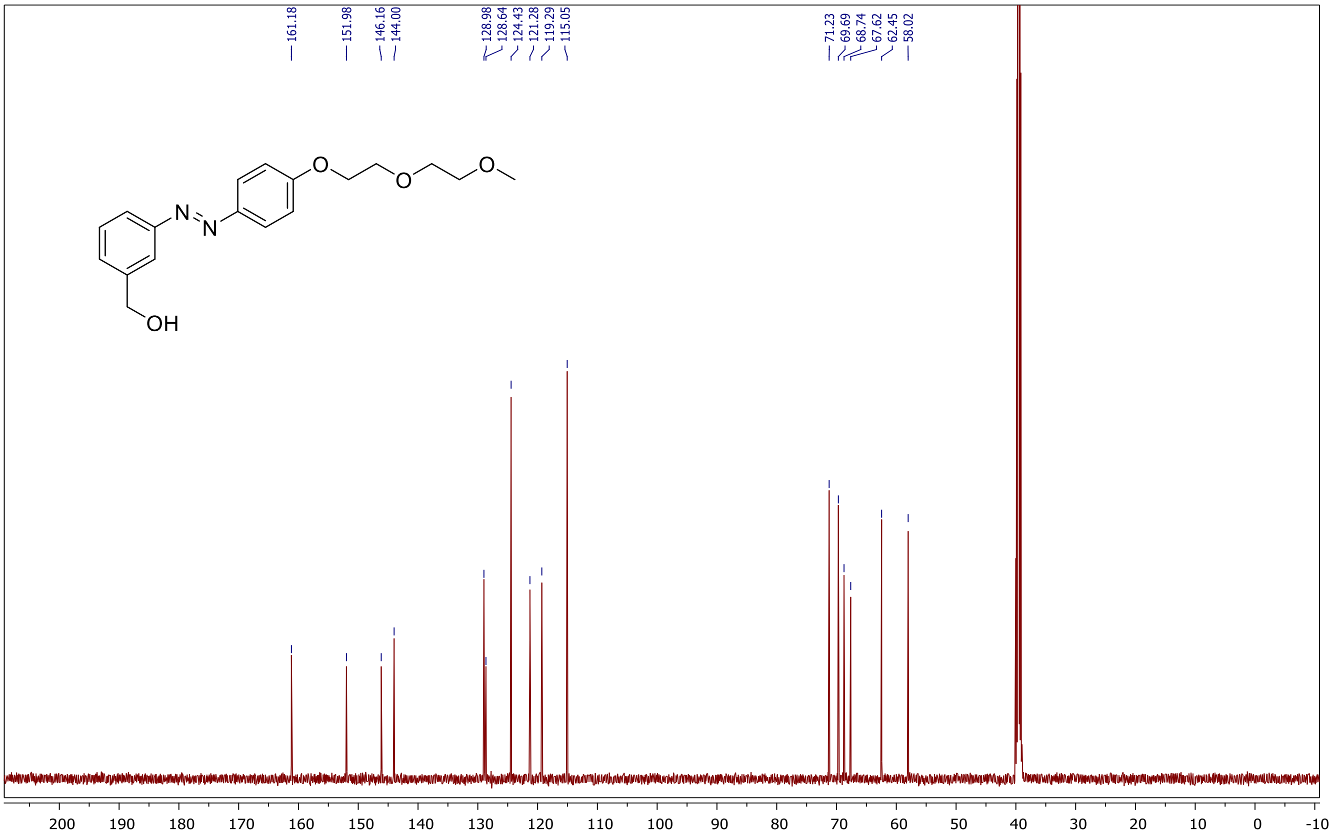

Figure S6: ^{13}C NMR (125 MHz, d_6-DMSO) spectrum of **5**.

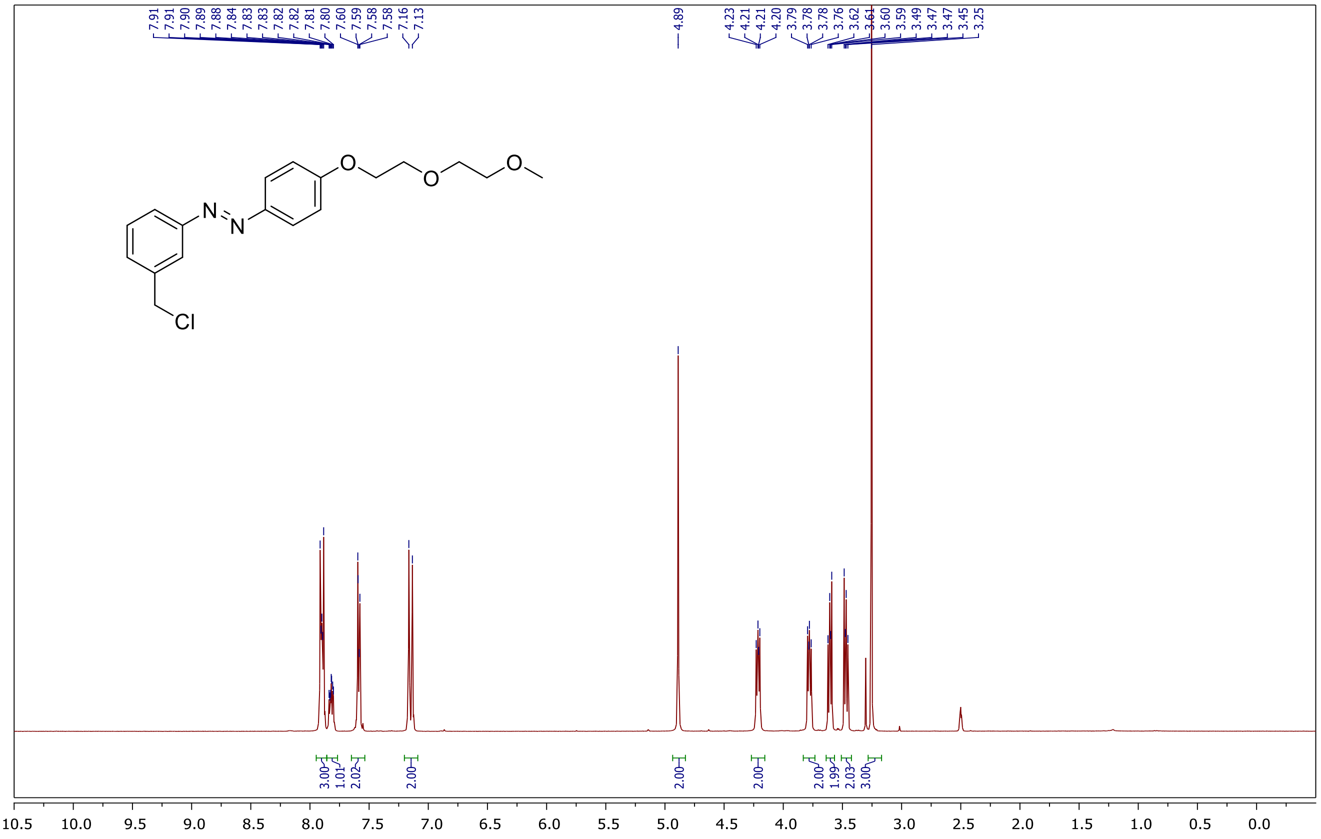

Figure S7: ^{1}H NMR (300 MHz, d_6-DMSO) spectrum of **3a**.

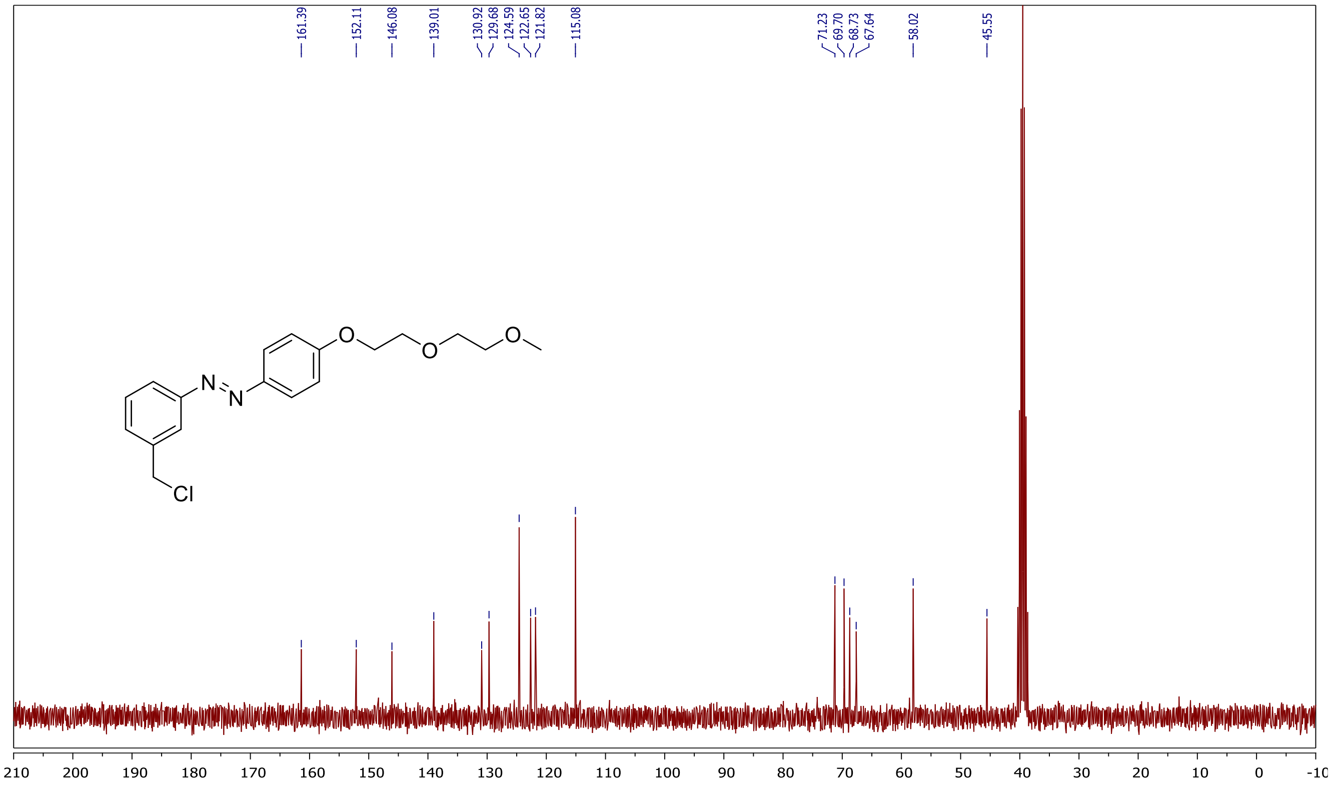

Figure S8: ^{13}C NMR (75 MHz, d_6-DMSO) spectrum of **3a**.

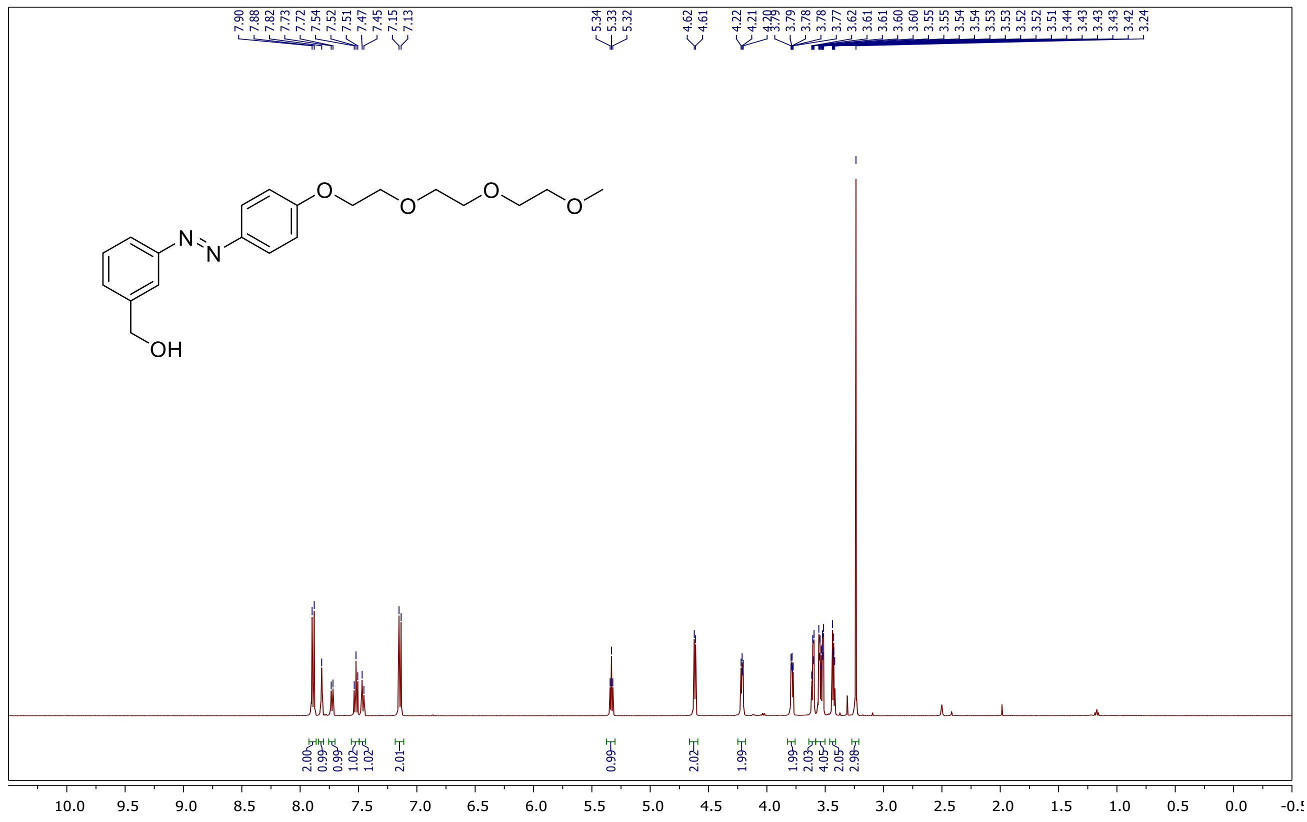

Figure S9: ^{1}H NMR (500 MHz, d_6-DMSO) spectrum of **6**.

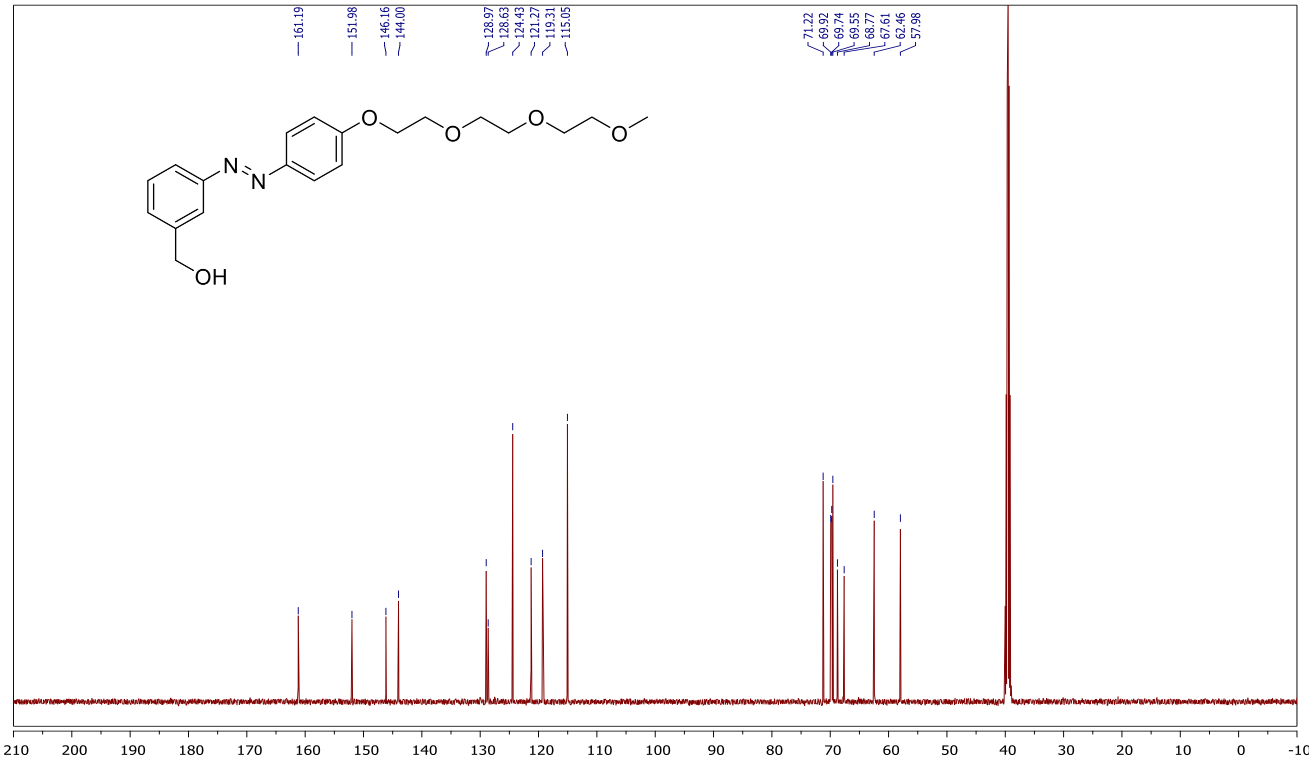

Figure S10: ^{13}C NMR (125 MHz, d_6-DMSO) spectrum of **6**.

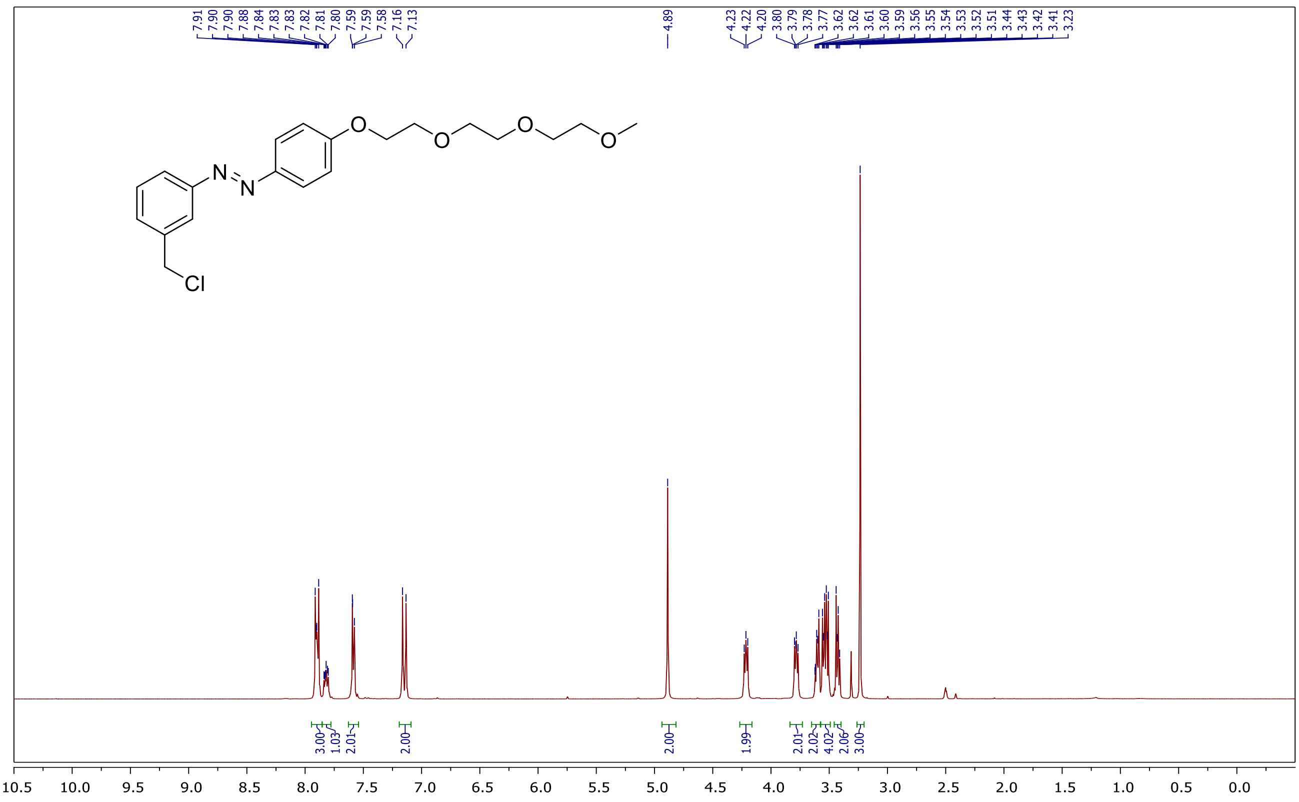

Figure S11: ^{1}H NMR (300 MHz, d_6-DMSO) spectrum of **3b**.

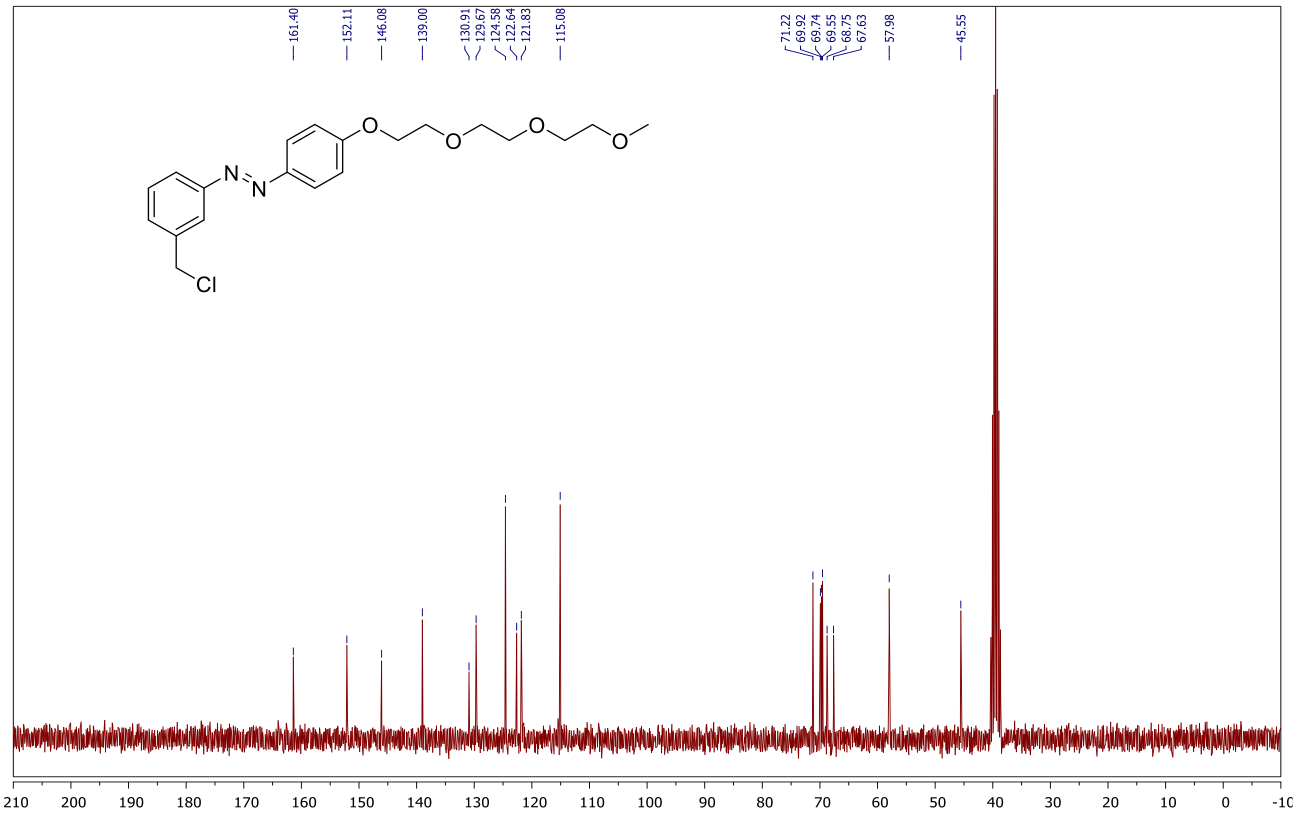

Figure S12: ^{13}C NMR (75 MHz, d_6-DMSO) spectrum of **3b**.

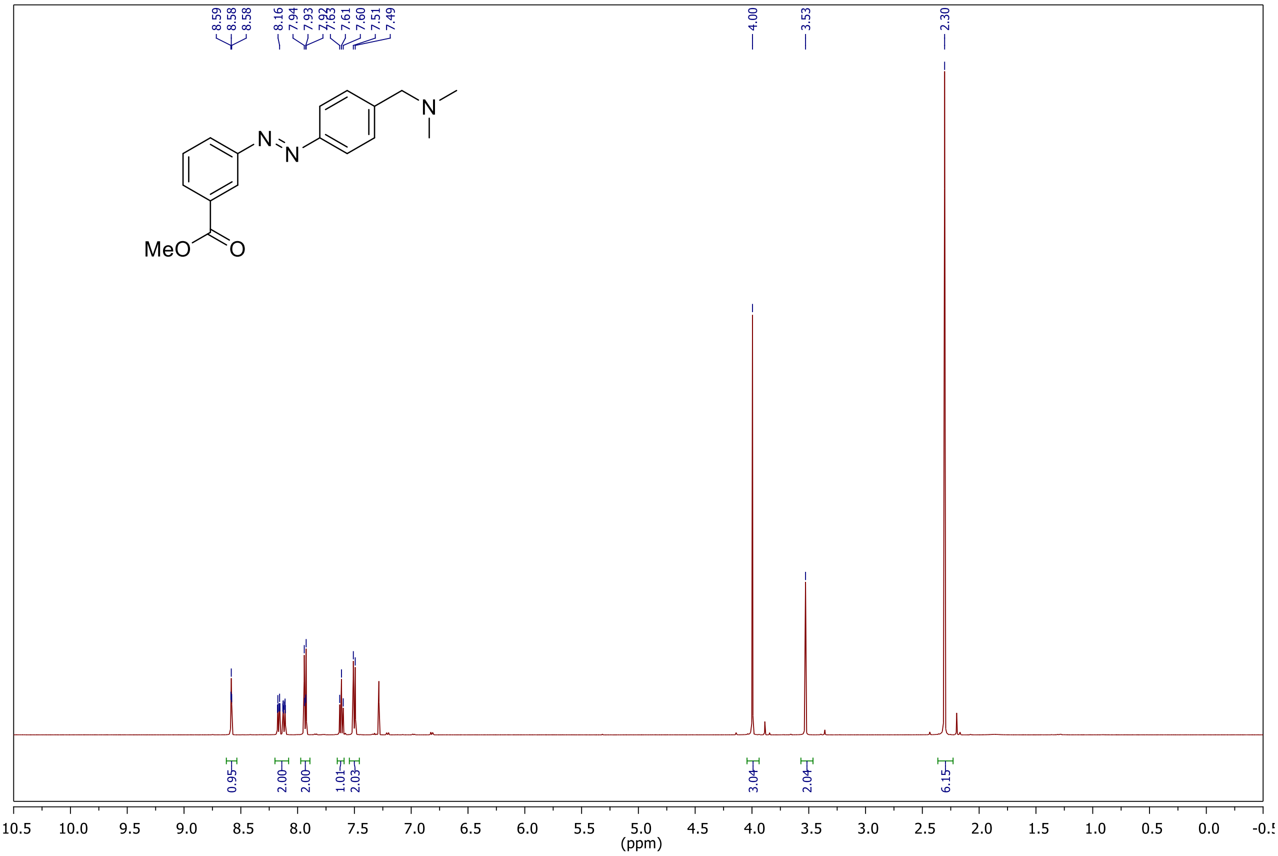

Figure S13: ^{1}H NMR (500 MHz, $CDCl_3$) spectrum of **7**.

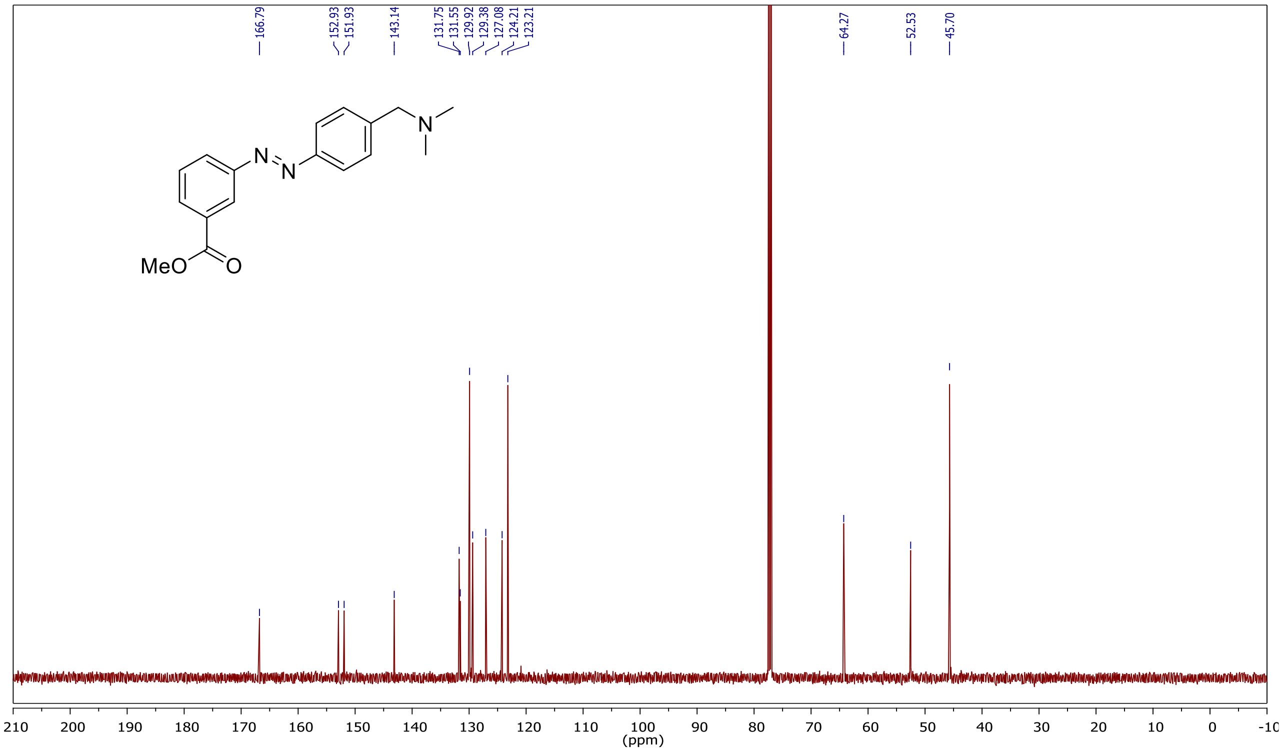

Figure S14: ^{13}C NMR (125 MHz, $CDCl_3$) spectrum of **7**.

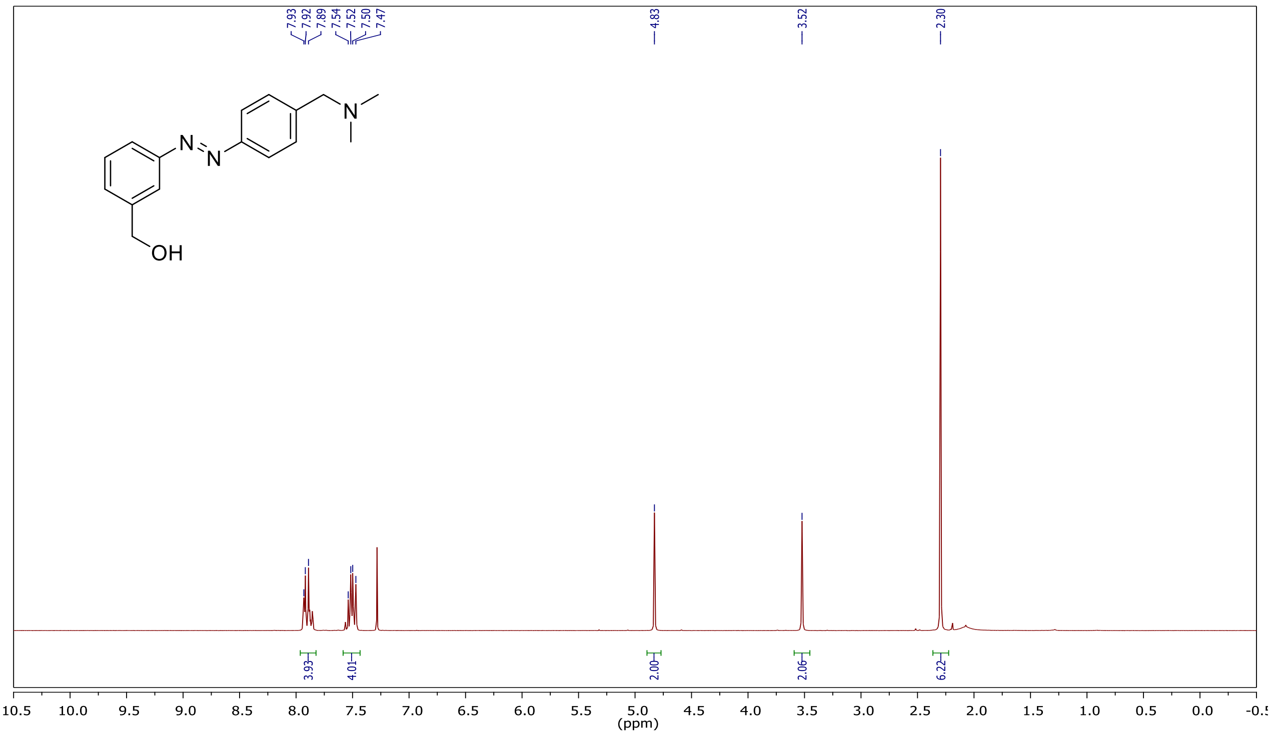

Figure S15: ^{1}H NMR (500 MHz, $CDCl_3$) spectrum of **8**.

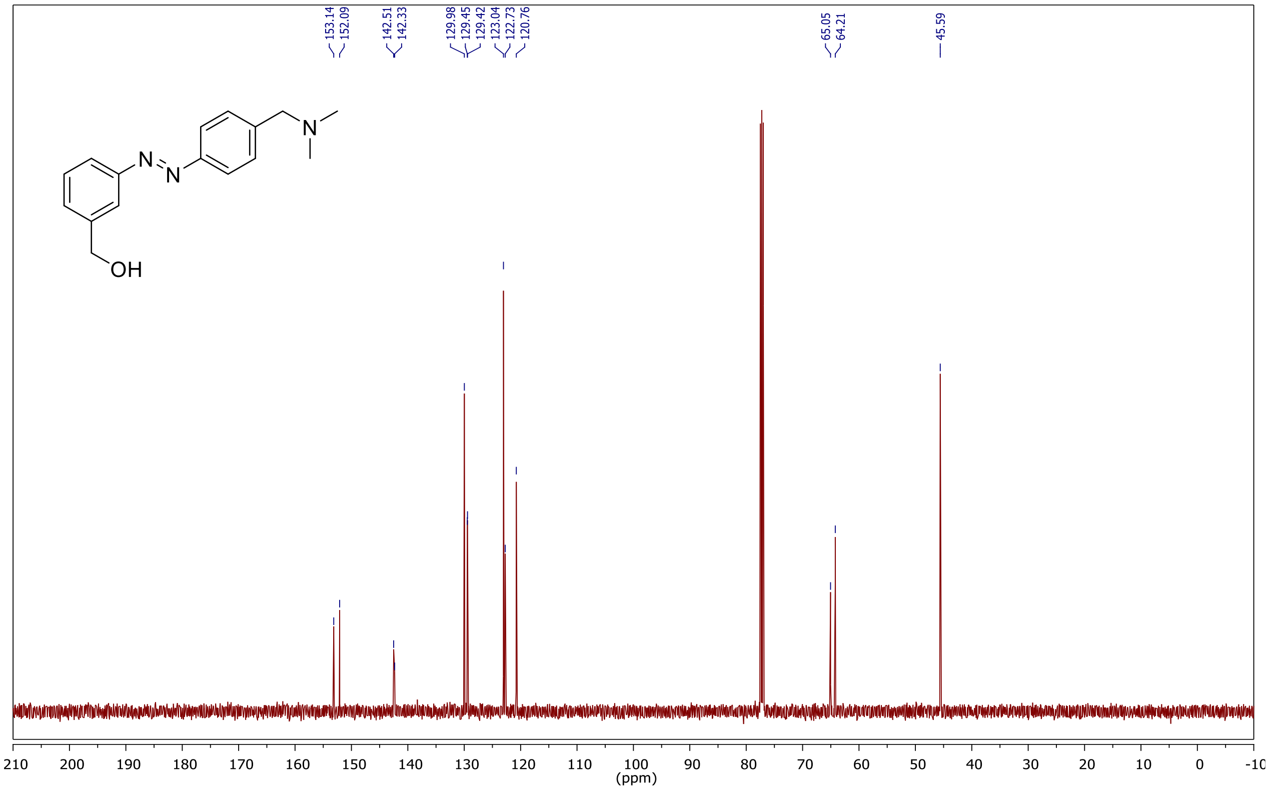

Figure S16: ^{13}C NMR (125 MHz, $CDCl_3$) spectrum of **8**.

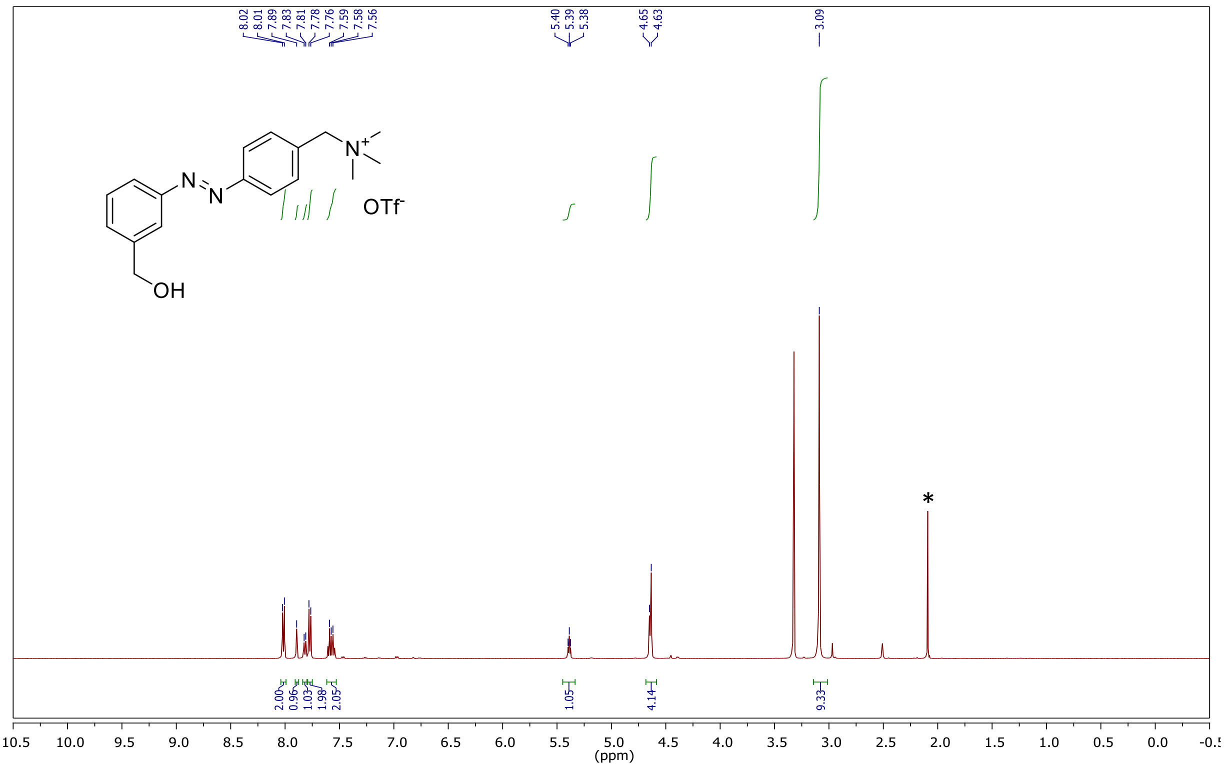

Figure S17: ^{1}H NMR (500 MHz, d_6–DMSO) spectrum of **9**, asterisk denotes residual acetone.

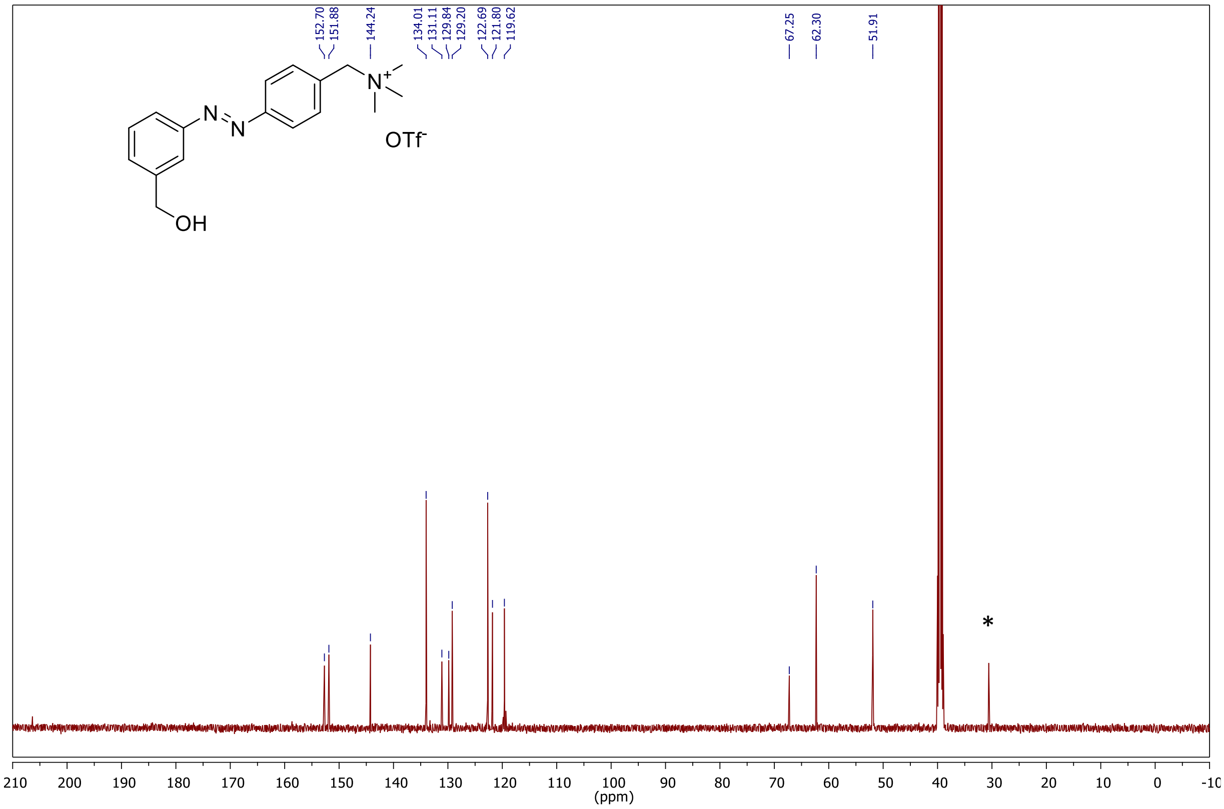

Figure S18: ^{13}C NMR (125 MHz, d_6–DMSO) spectrum of **9**, asterisk denotes residual acetone.

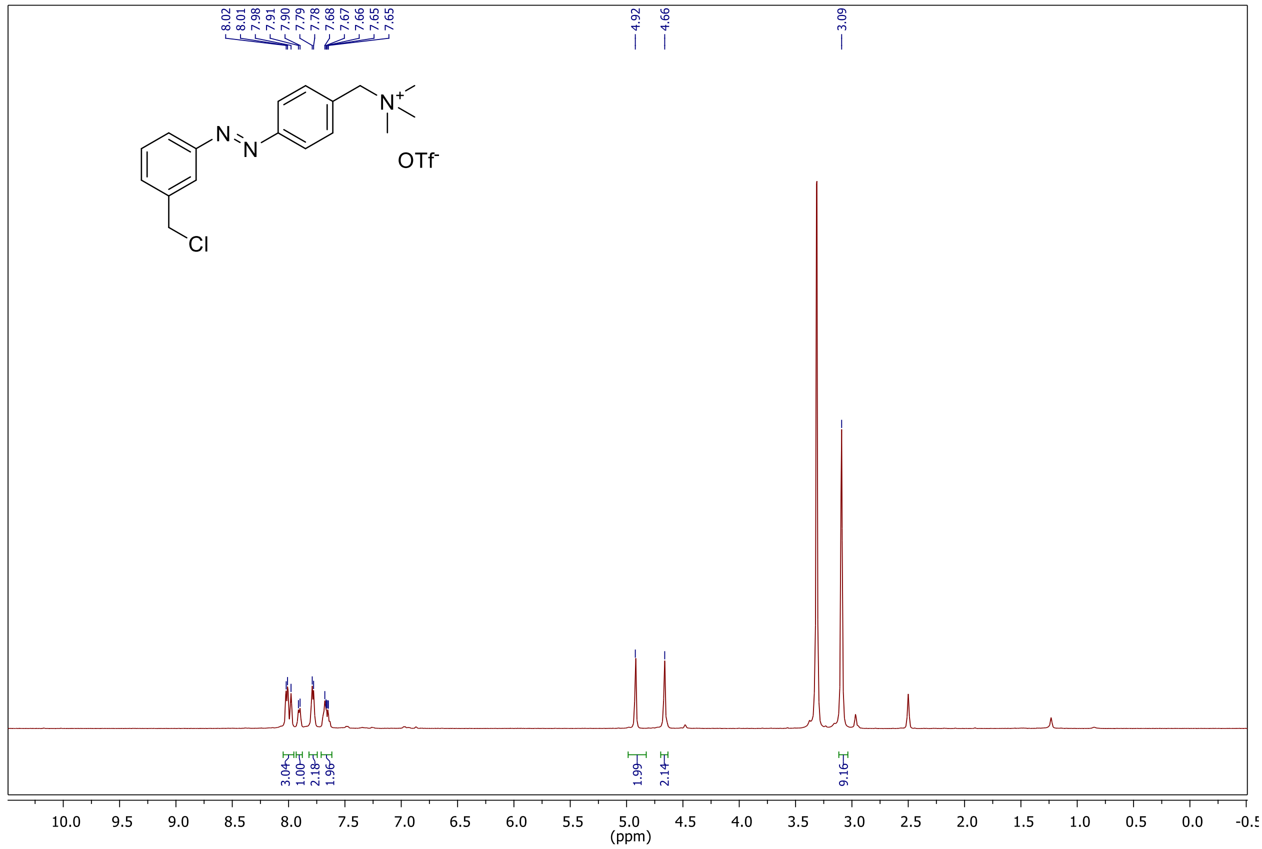

Figure S19: ^{1}H NMR (500 MHz, d_6-DMSO) spectrum of **3c**.

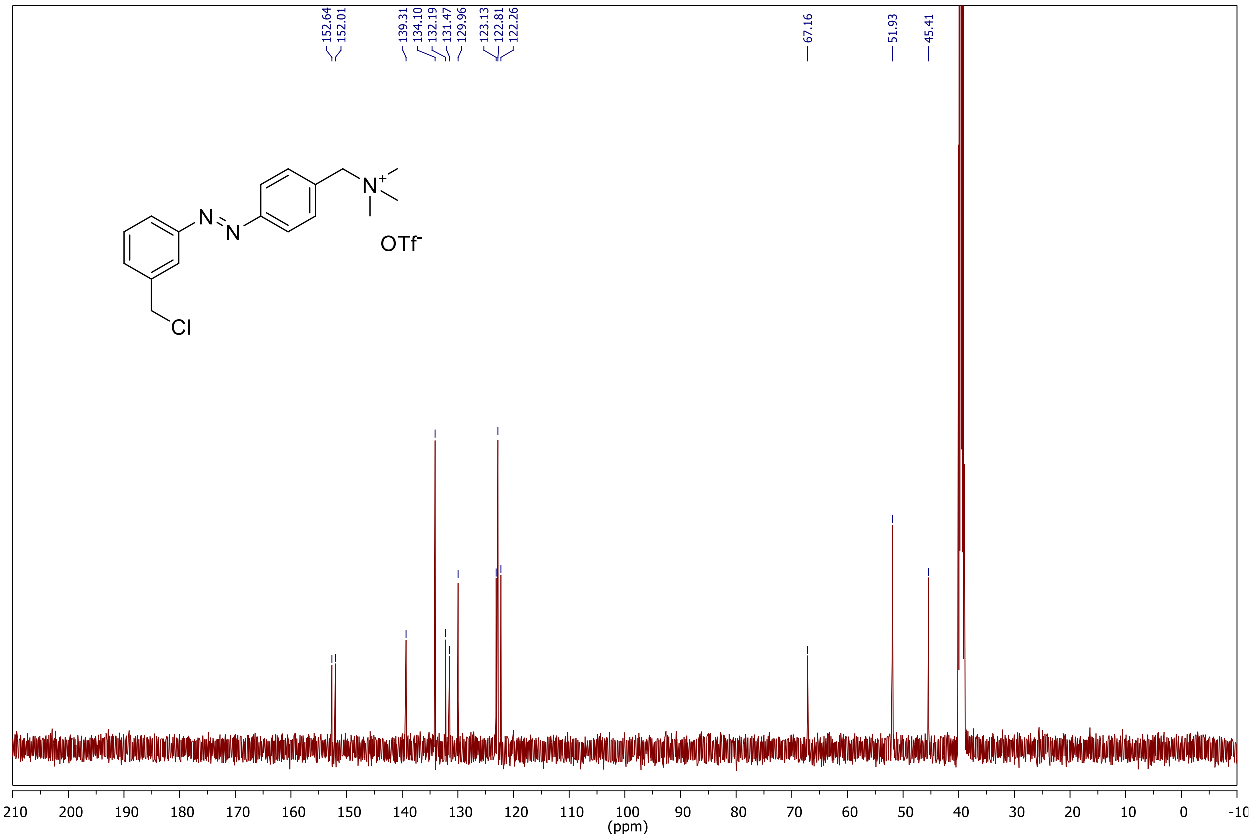

Figure S20: ^{13}C NMR (125 MHz, d_6-DMSO) spectrum of **3c**.

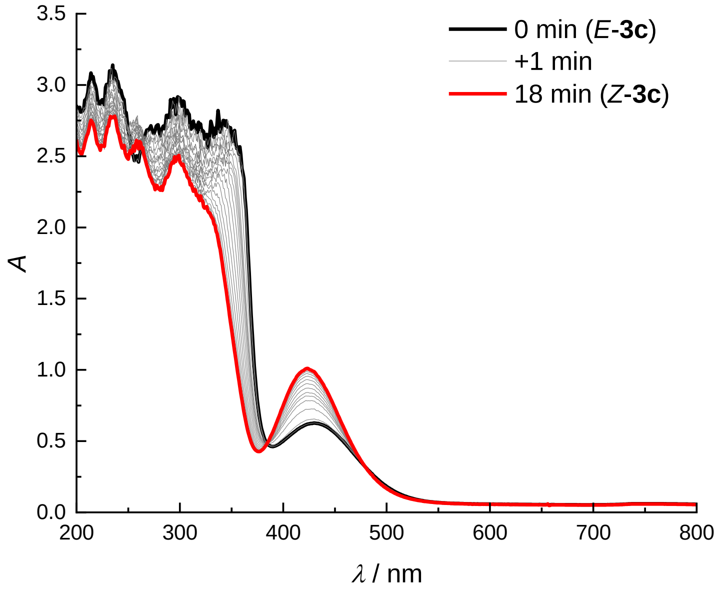

Figure S21: Absorption spectra recorded during the preparation of *Z*-**3c** from *E*-**3c** (c(*E*-**3c**) ≈ 4 × 10^{-4} M) reaching the photostationary phase at 320 nm in glycine buffer (100 mM, pH = 8.6) using a Xe lamp (450 W) equipped with a monochromator (320 nm).

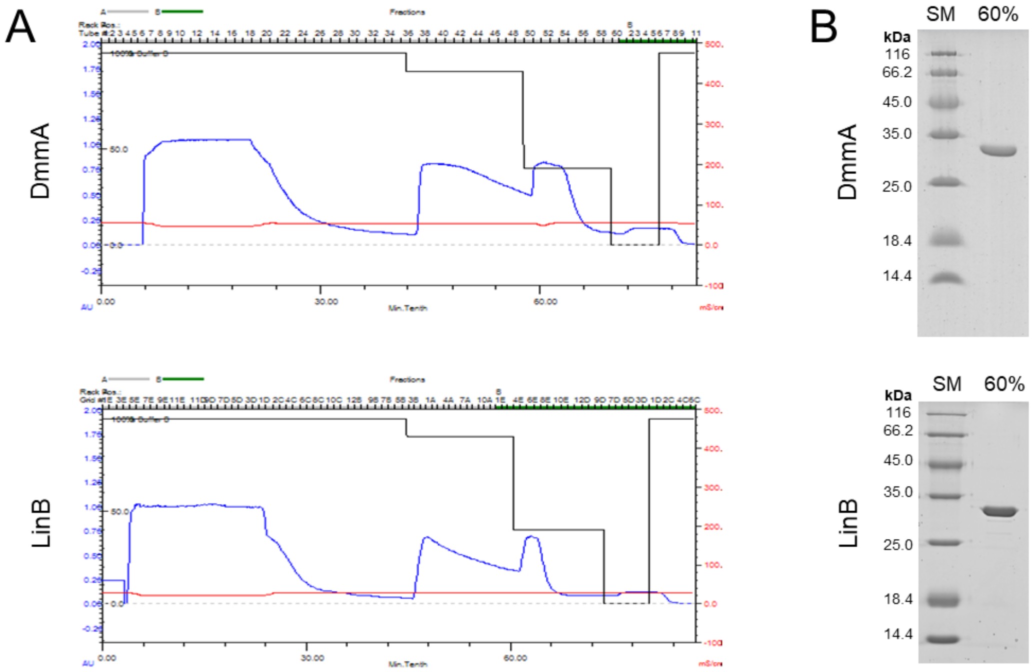

Figure S22: (A) Chromatograms from purification by metal-affinity chromatography of DmmA and LinB. Black – buffer A gradient (10 mM imidazole). Blue – UV (280 nm) absorbance signal. Red – conductivity. The target protein was eluted by 10% and 60% gradient of purification buffer B (50 mM and 300 mM imidazole, respectively). (B) SDS-PAGE of purified enzymes. Lane SM: molecular weight marker; lane 60%: protein eluted by 60% gradient of buffer B.

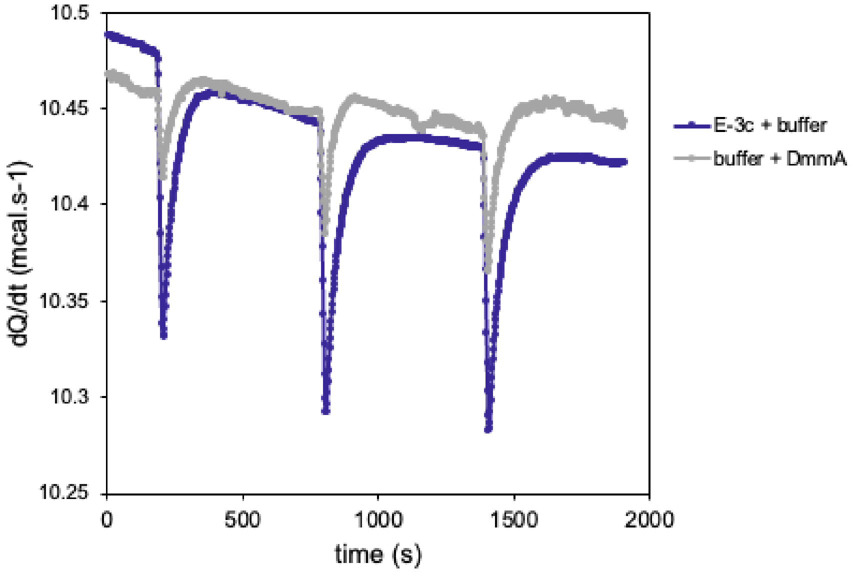

Figure S23: Heat flow measured on ITC as a result of buffer titration to *E*-**3c** solution (blue) and DmmA titration to a buffer solution (grey). Both experiments lead to the release of heat related to mixing only.

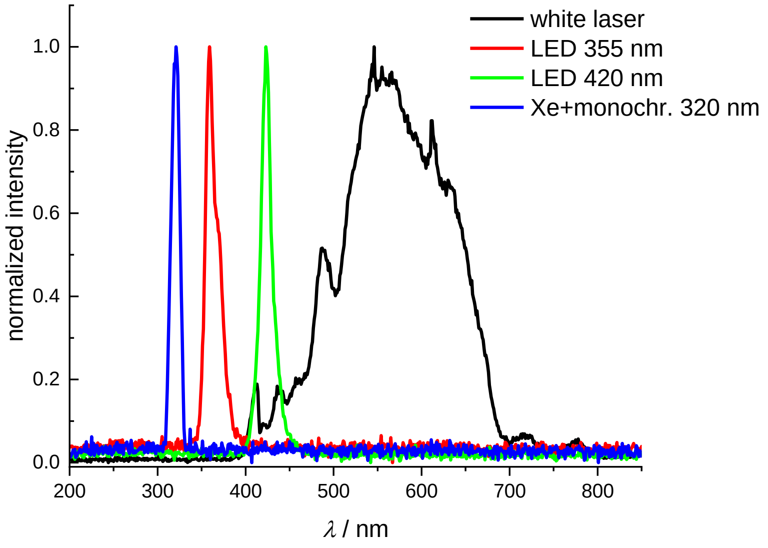

Figure S24: Emission spectrum of light sources used for photoisomerization experiments: white laser (λ_{em} > 400 nm) equipped with a hot mirror (black line), LED 355 nm (red line), LED 420 nm (green line), Xe lamp equipped with a monochromator (320 nm, $\Delta\lambda_{1/2}$ = 12 nm, blue line).

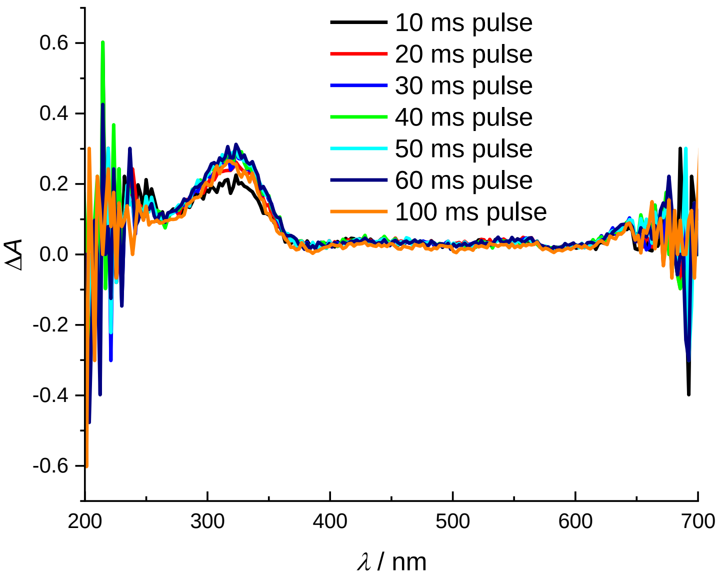

Figure S25: Differential absorption spectra recorded upon irradiation of a solution of *Z*-**3c** (prepared from *E*-**3c**; *c*(*E*-**3c**) ≈ 4 × 10^{-4} M) in glycine buffer (100 mM, pH = 8.6) using white laser (400-700 nm) pulses of various durations aimed toward a stopped-flow cuvette (optical path *l* = 0.8 mm).

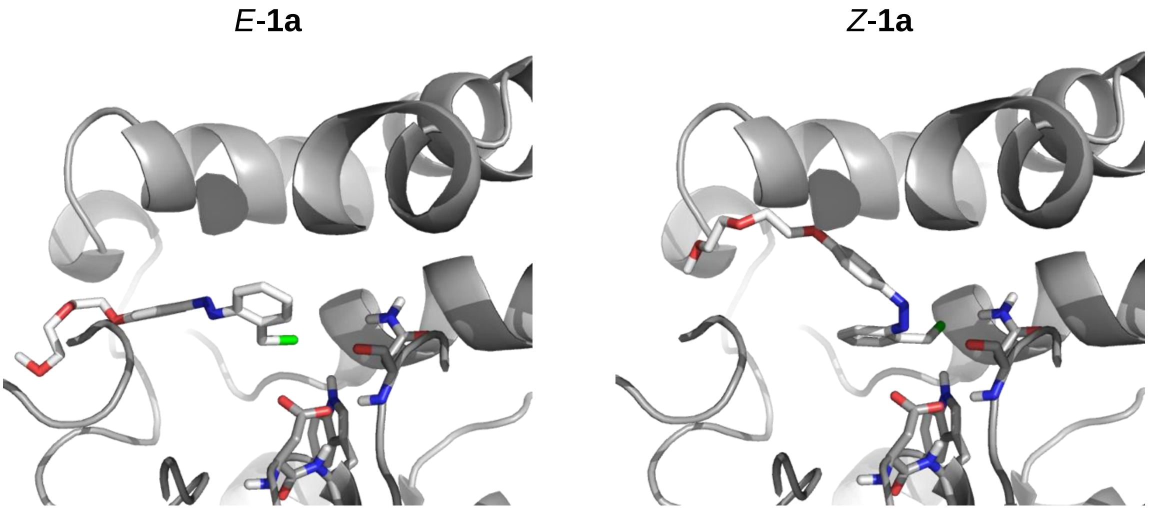

E-**1b**

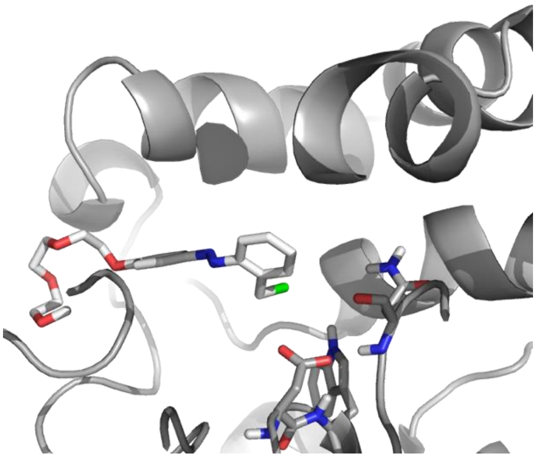

Z-**1b**

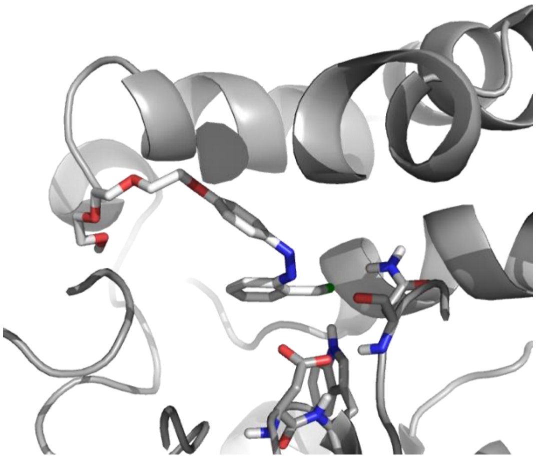

E-**1c**

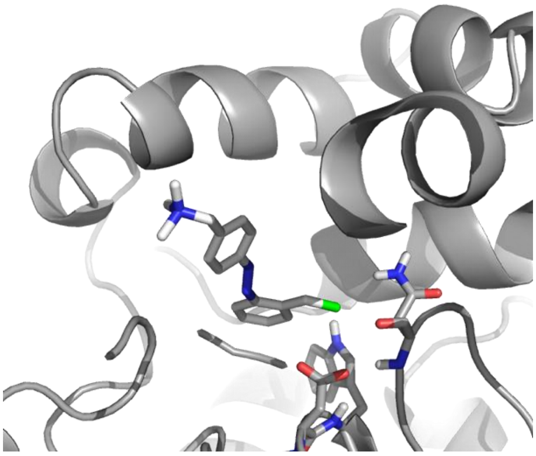

Z-**1c**

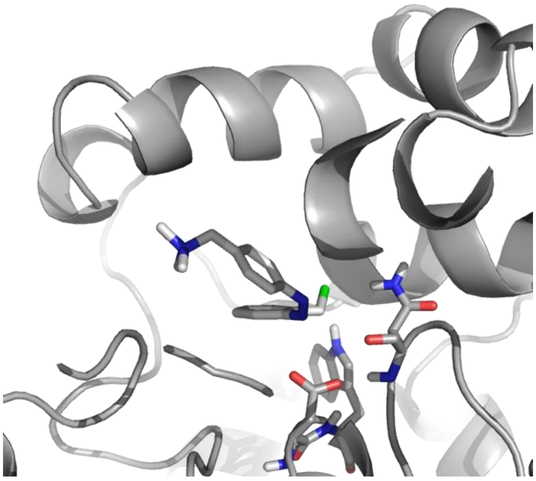

E-**2***

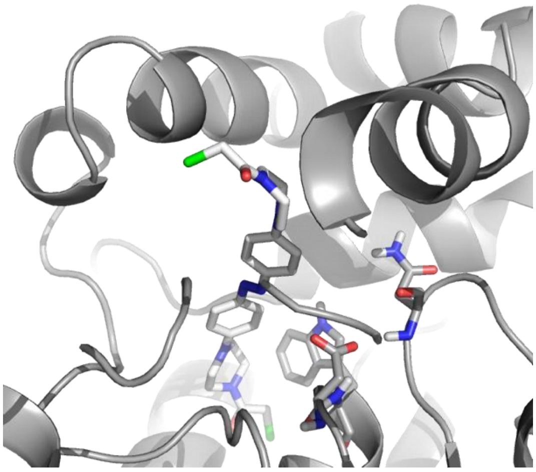

Z-**2**

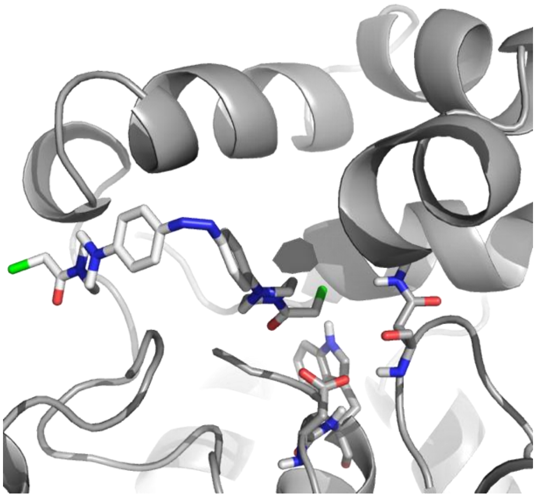

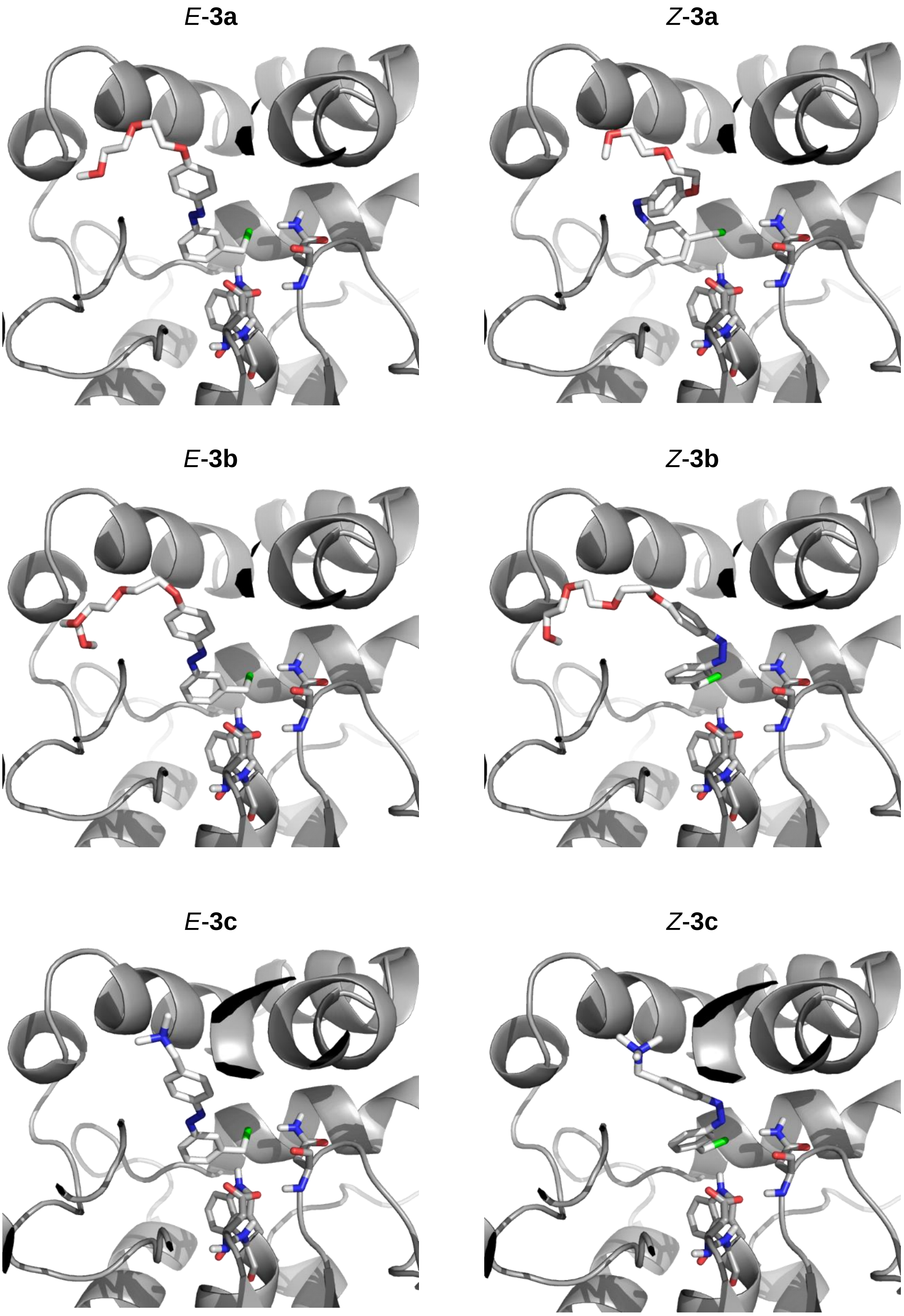

Figure S26: Binding modes of substrates *E*/*Z*-**1a**-**1c**, *E*/*Z*-**2** and *E*/*Z*-**3a**-**3c** docked into the active site of DmmA, in their best reactive conformations. The ligands and the catalytic residues (N78, D144, and W145) are represented as sticks. *No reactive binding mode was found for *E*-**2**, for which case the best binding conformation is shown.

E-**1a**

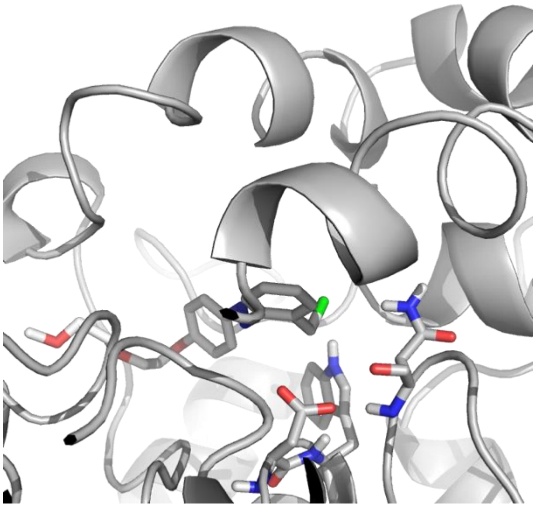

Z-**1a**

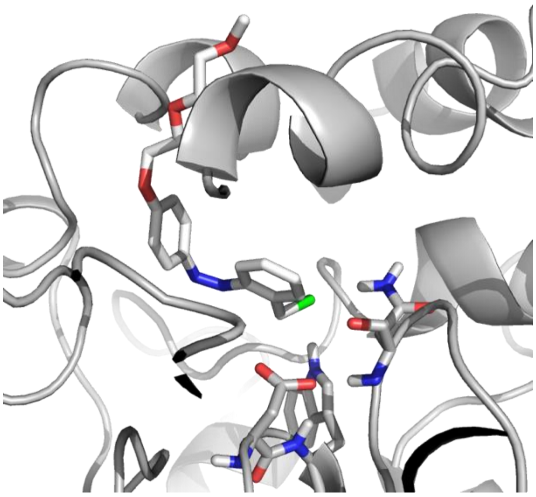

E-**1b**

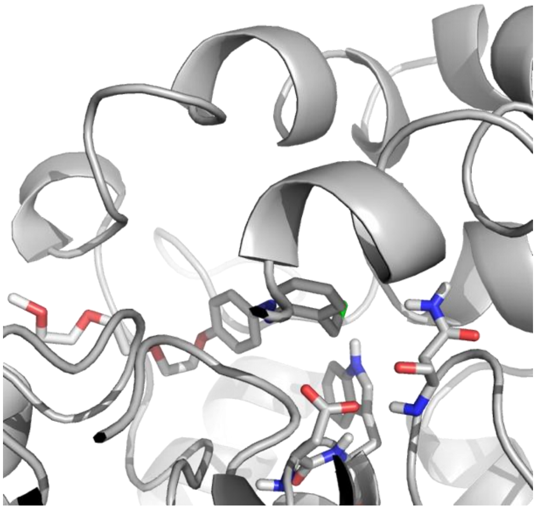

Z-**1b**

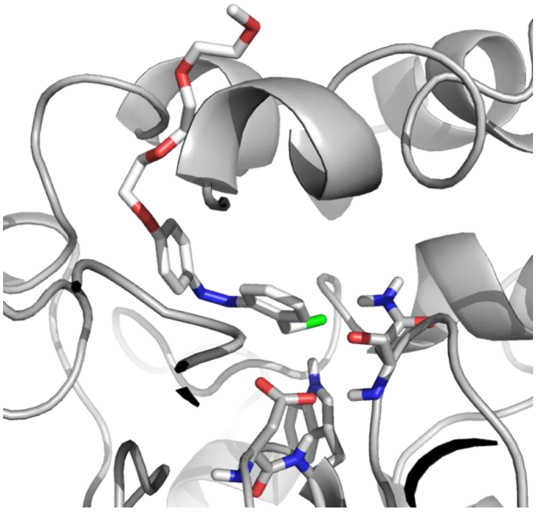

E-**1c**

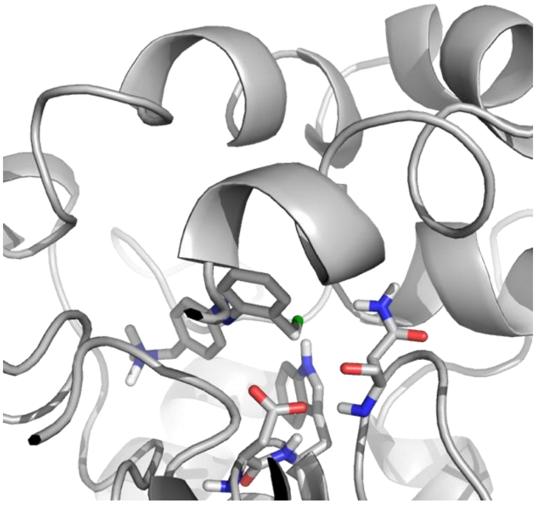

Z-**1c**

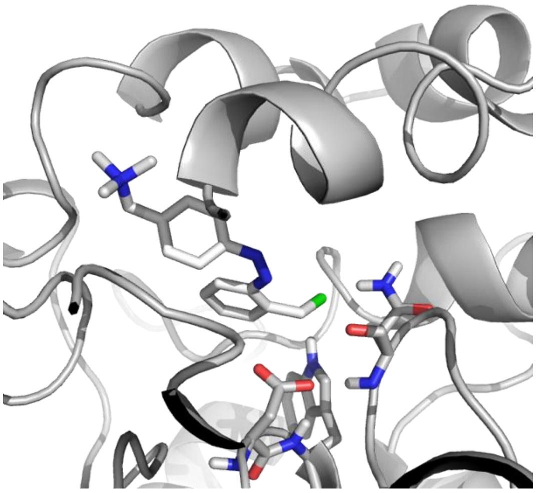

E-**2***

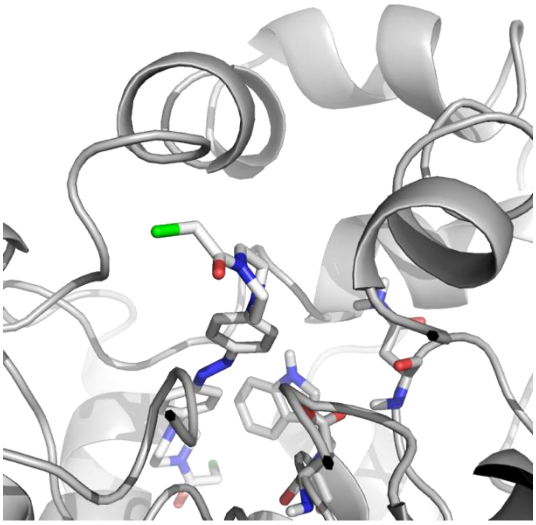

Z-**2***

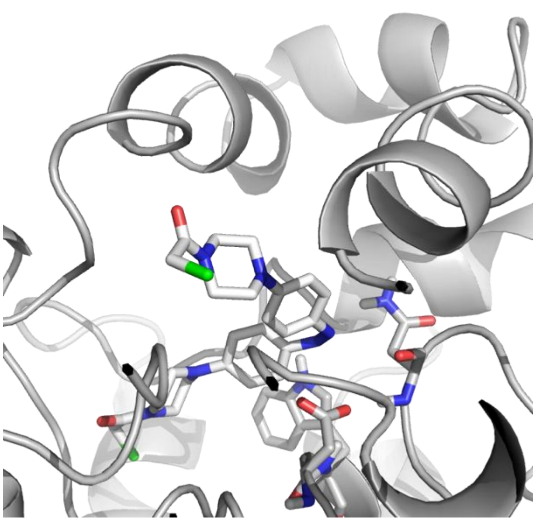

E-**3a**

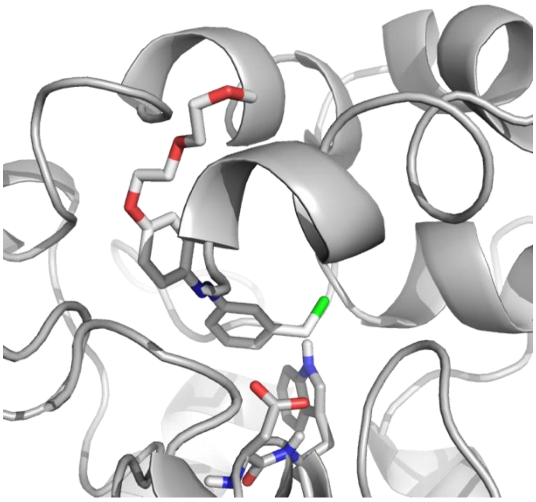

Z-**3a***

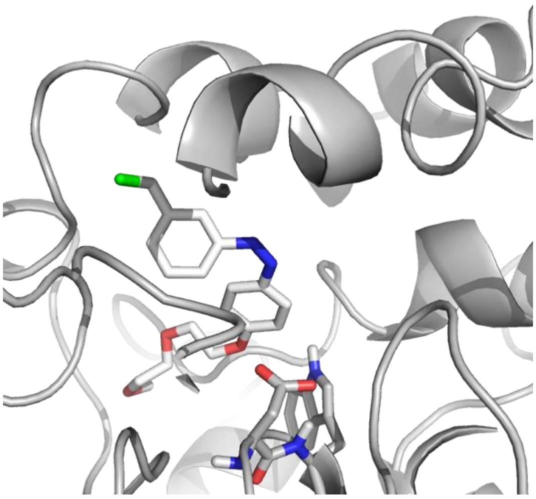

E-**3b**

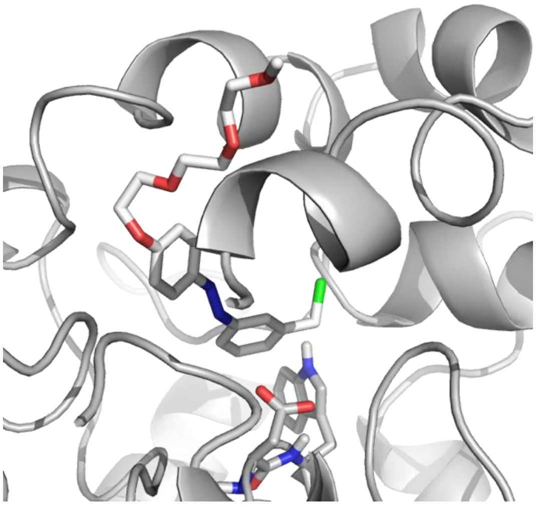

Z-**3b***

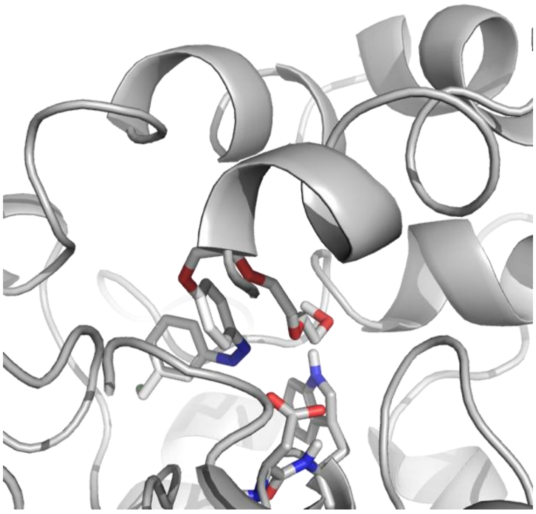

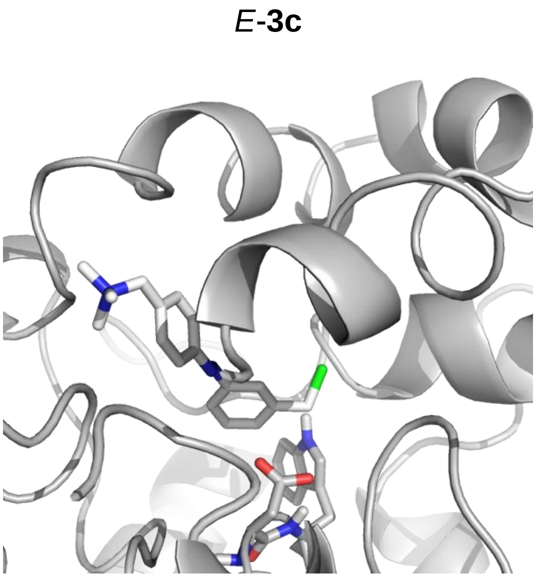

Figure S27: Binding modes of the *E*/*Z*-**1a-1c**, *E*/*Z*-**2** and *E*/*Z*-**3a-3c** substrates docked into the active site of LinB, in their best reactive conformations. The ligands and the catalytic residues (N38, D108, and W109) are represented as sticks. *No reactive binding modes were found for *E*/*Z*-**2**, and *E*-**3a-3c**, for which the best binding conformations are shown.

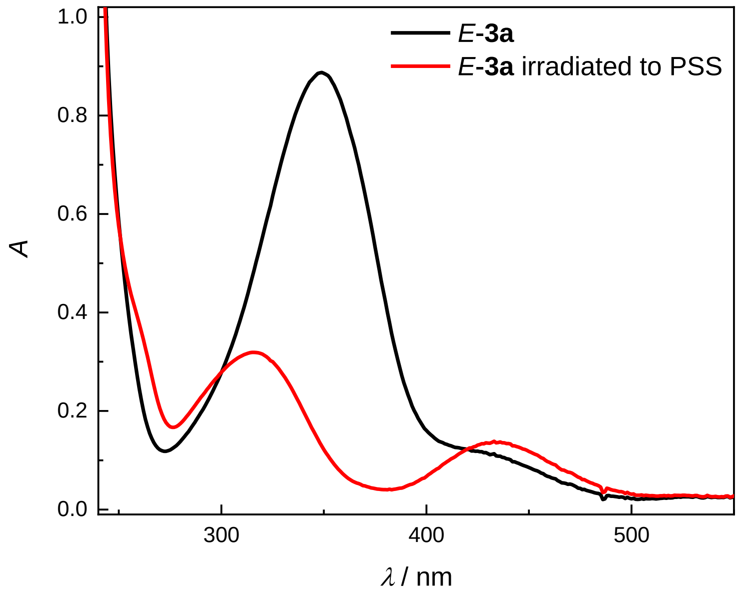

Figure S28: Absorption spectra recorded during irradiation of a solution of *E*-**3a** in PBS (100 mM, pH 7.4, black solid) to the photostationary state (red solid) with UV LED (375 nm) followed by UV/VIS spectroscopy.

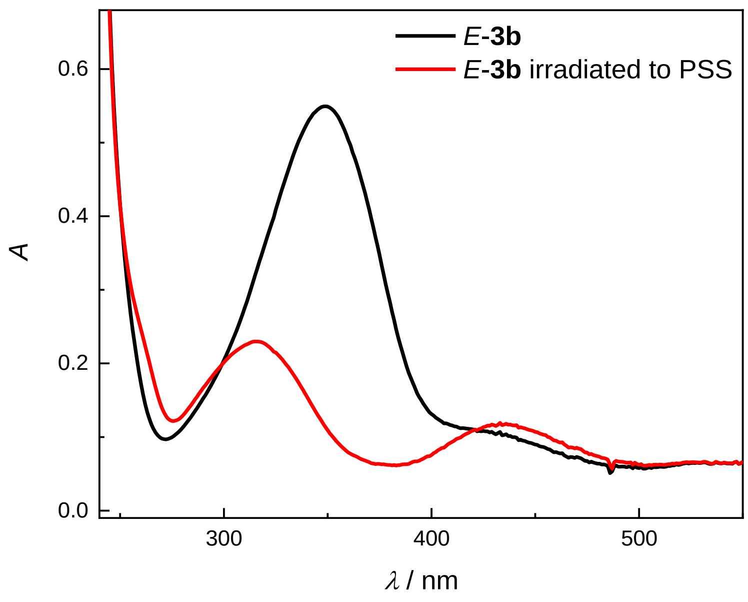

Figure S29: Absorption spectra recorded during irradiation of a solution *E*-**3b** in PBS (100 mM, pH 7.4, black solid) to the photostationary state (red solid) with UV LED (375 nm) followed by UV/VIS spectroscopy.

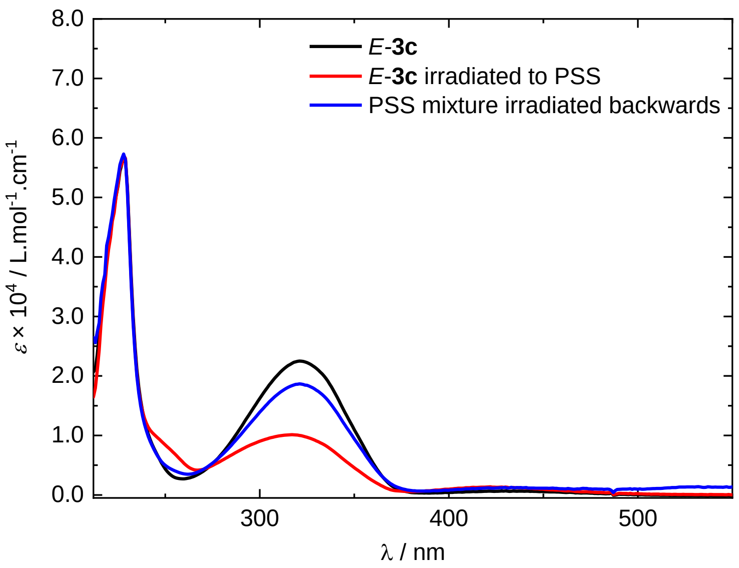

Figure S30: Absorption spectra recorded during irradiation of a solution *E*-**3c** in PBS (100 mM, pH 7.4, black solid) to the photostationary state (red solid) with UV LED (375 nm) and back with blue LED (400 nm) to give *E*-**3c** followed by UV/VIS spectroscopy.

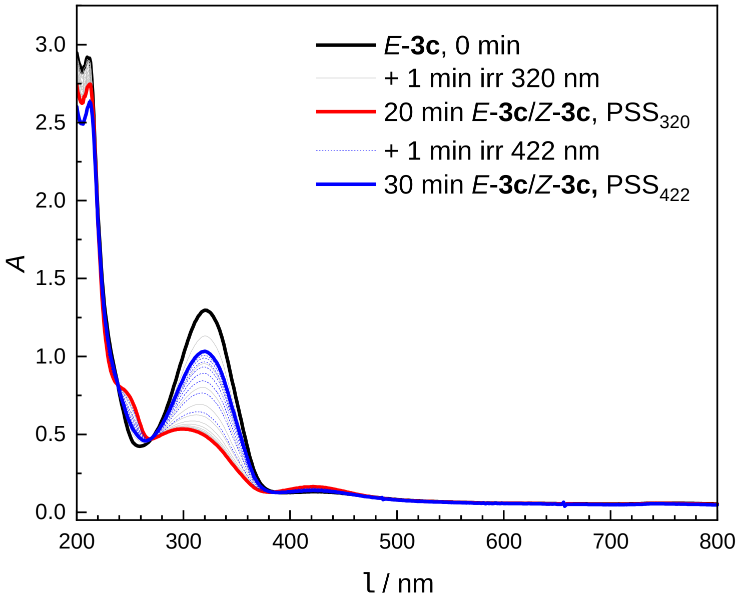

Figure S31: Irradiation of a solution of *E*-**3c** in glycine buffer (100 mM, pH 8.6, black solid) to give a photostationary state at 320 nm (red solid) with a Xe lamp (450 W) equipped with a monochromator (320 nm), and back to obtain a new photostationary state at 422 nm (blue solid) with a Xe lamp (450 W) equipped with a monochromator (422 nm), followed by UV/VIS spectroscopy.

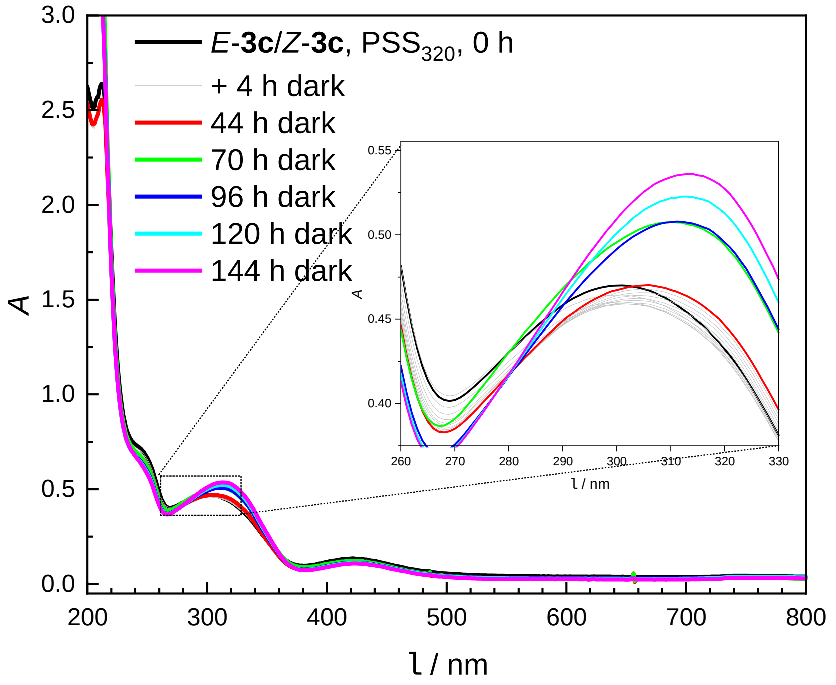

Figure S32: Stability of *Z*-**3c** in glycine buffer (100 mM, pH 8.6) in the dark at a photostationary state obtained at 320 nm irradiation, containing a mixture *E*-**3c**/*Z*-**3c.**

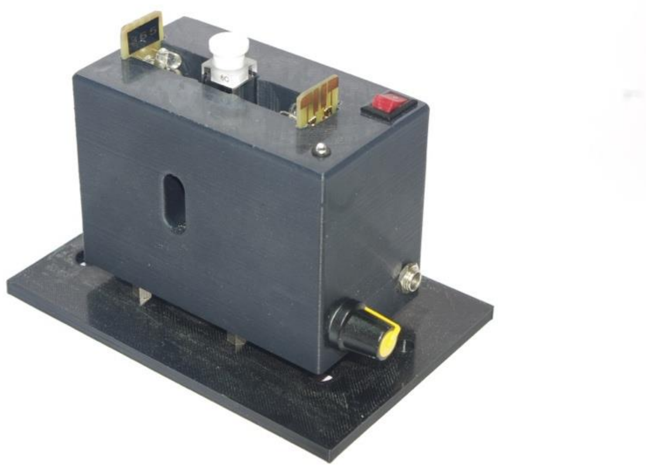

Figure S33: A custom-made 3D-printed LED reactor (two plug-in LED modules, 14 low-power LEDs, λ_{max} = 355 nm) per module, an integrated cooling fan, and a stirring pad with adjustable speed) used for simultaneous irradiation and UV/VIS spectroscopy measurements of the samples in 1.0 cm cuvettes.

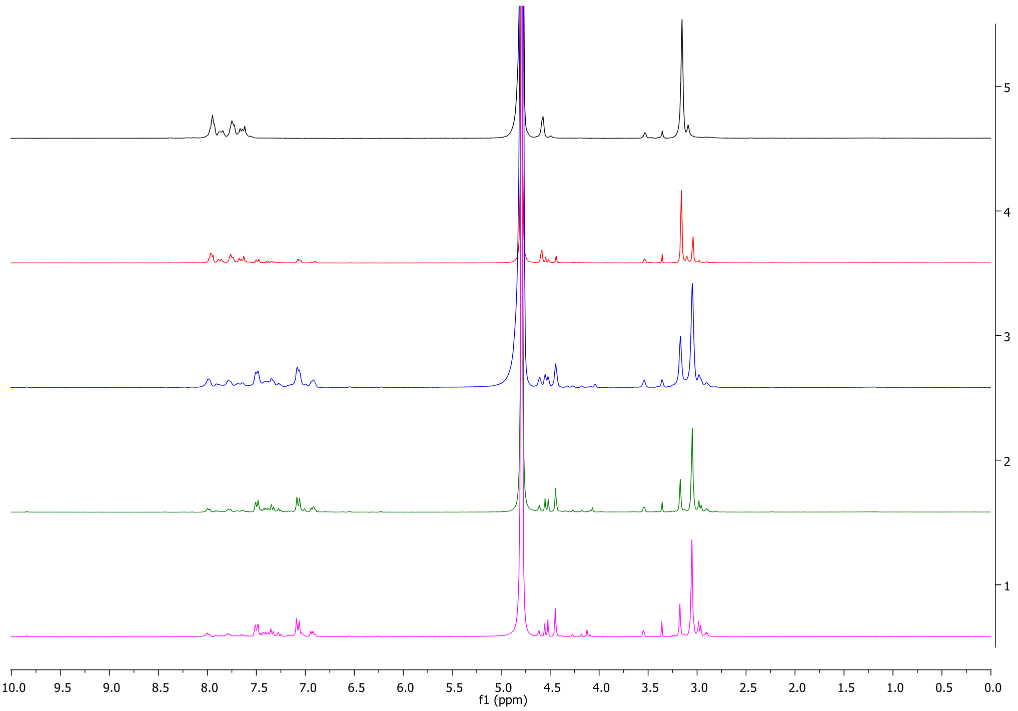

Figure S34: ^{1}H NMR (300 MHz, 0.1 M d_5-glycine in D_2O buffer, pH = 8.6) spectra of *E*-**3c** (0 h, 95:5 (*n*/*n*), black solid) during 320 nm irradiation (1 h, 74:26 (*n*/*n*), red solid; 20 h, 37:63 (*n*/*n*), blue solid; 27 h, 27:73 (*n*/*n*), green solid) to reach the photostationary state (44 h, magenta solid), containing a mixture *E*-**3c**/*Z*-**3c** in the ratio of 25:75 (*n*/*n*).

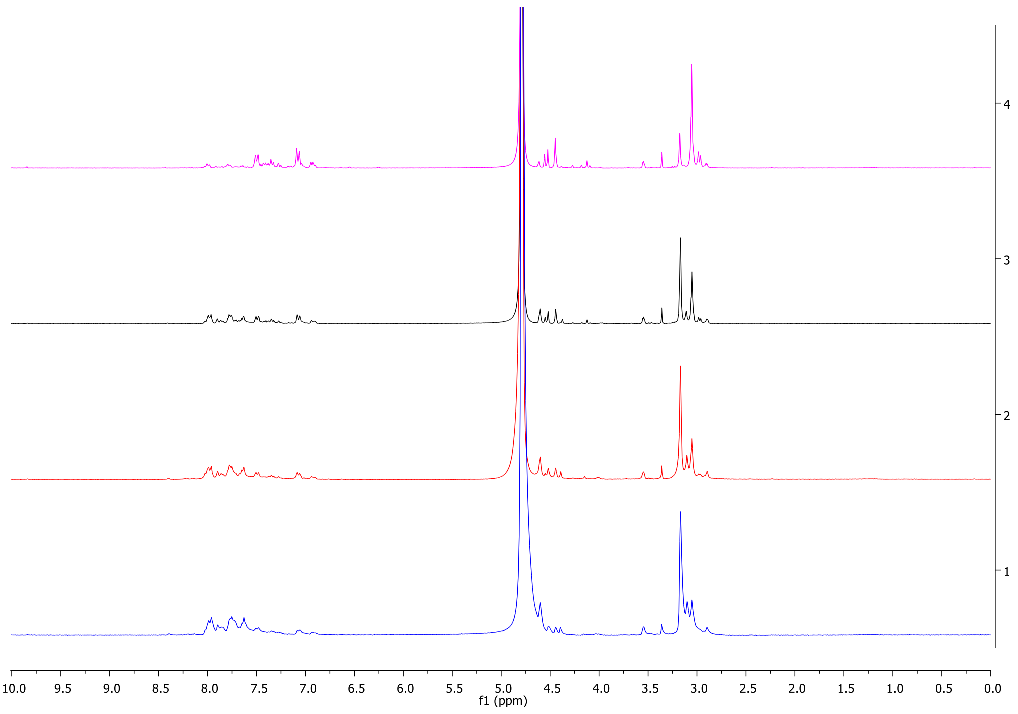

Figure S35: ^{1}H NMR (300 MHz, 0.1 M d_5-glycine in D_2O buffer, pH = 8.6) spectra of mixture in photostationary state at 320 nm (magenta solid, identical as in **Figure S34**) during irradiation (3.5 h, 62:38 (*n*/*n*), black solid; 4.5 h, 74:26 (*n*/*n*), red solid) at 422 nm to reach the corresponding photostationary state (27.5 h, blue solid), containing a mixture *E*-**3c**/*Z*-**3c** in the ratio of 75:25 (*n*/*n*).

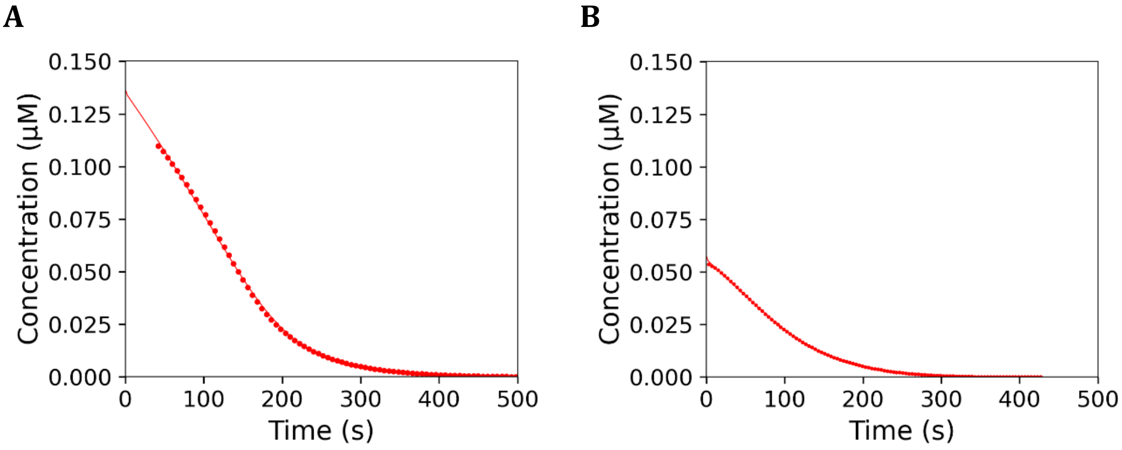

C

	parameter	unit	value	s.e.
E-**3a**	k_{cat}/K_m	$s^{-1}.\mu M^{-1}$	87	51
	K_{SI}	μM	0.004	0.003
Z-**3a** + E-**3a**	k_{cat}/K_m	$s^{-1}.\mu M^{-1}$	120	20
	K_{SI}	μM	0.004	0.001
	K_i	μM	0.035	0.001

Figure S36: Steady-state kinetics of DmmA with 3a. The progress curves for conversion of *E*-**3a** (A) and *Z*-**3c**+*E*-**3a** (B); solid lines represent the best fit. The data were analyzed by KinTek Explorer using steady-state kinetic models described in Figure 2, where K_m is Michaelis constant, k_{cat} is turnover number, K_i is competitive inhibition constant defining the formation of enzyme-*Z*-**3c** complex. The progress curves show a characteristic shape for substrate inhibition when the reaction rate (dS/dt, slope of the curve) is increased at the initial part of the substrate conversion (first 180 s) before the substrate concentration becomes limiting. Thus, the dissociation constant K_{SI} of the substrate inhibitory complex ESS, ES + S ⇆ ESS, was added to the kinetic model.

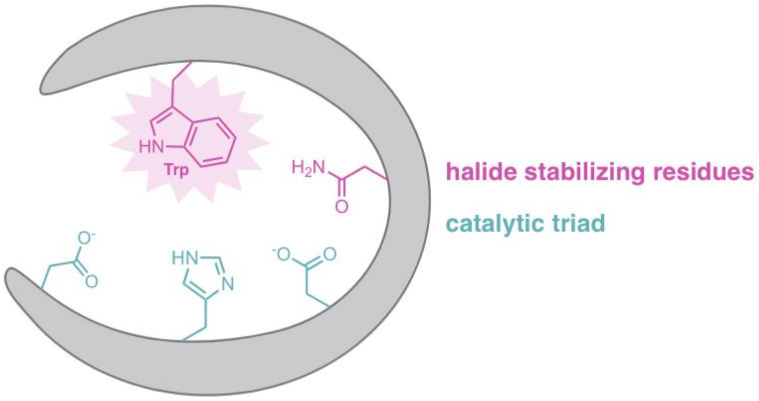

Figure S37: The active site of haloalkane dehalogenases. The residues of the catalytic triad are shown in teal, and the halide-stabilizing residues (including the fluorescent tryptophan) are shown in magenta.

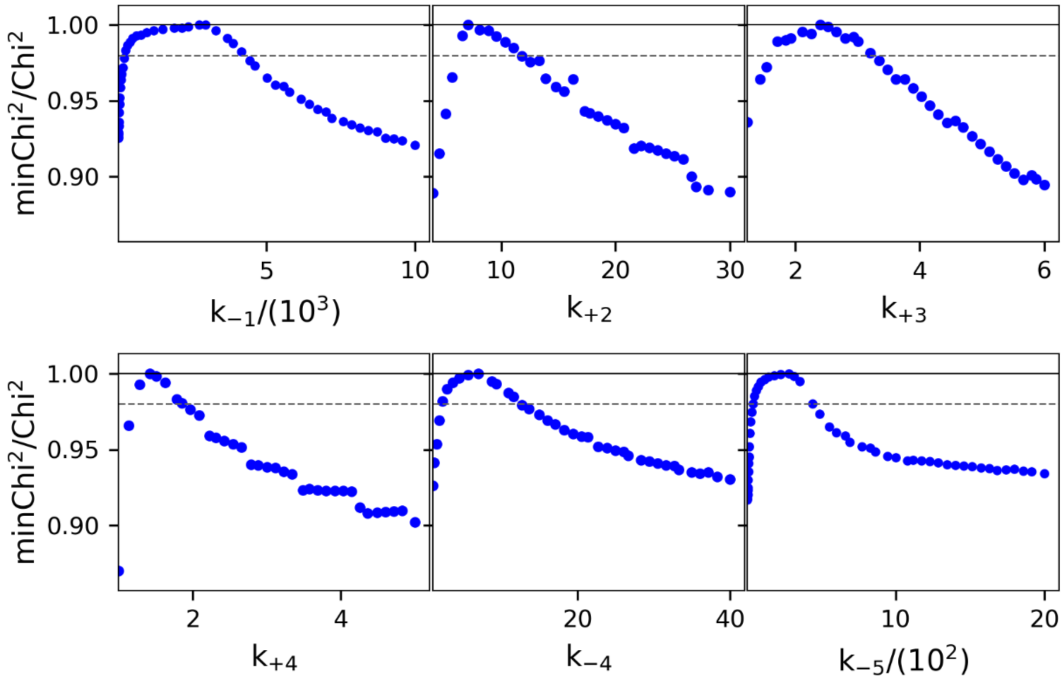

Figure S38: FitSpace 1D confidence contour analysis of the kinetic constants of DmmA catalysis. (K_1 = k_{-1}/1000, K_5 = 1000/k_{-5}).

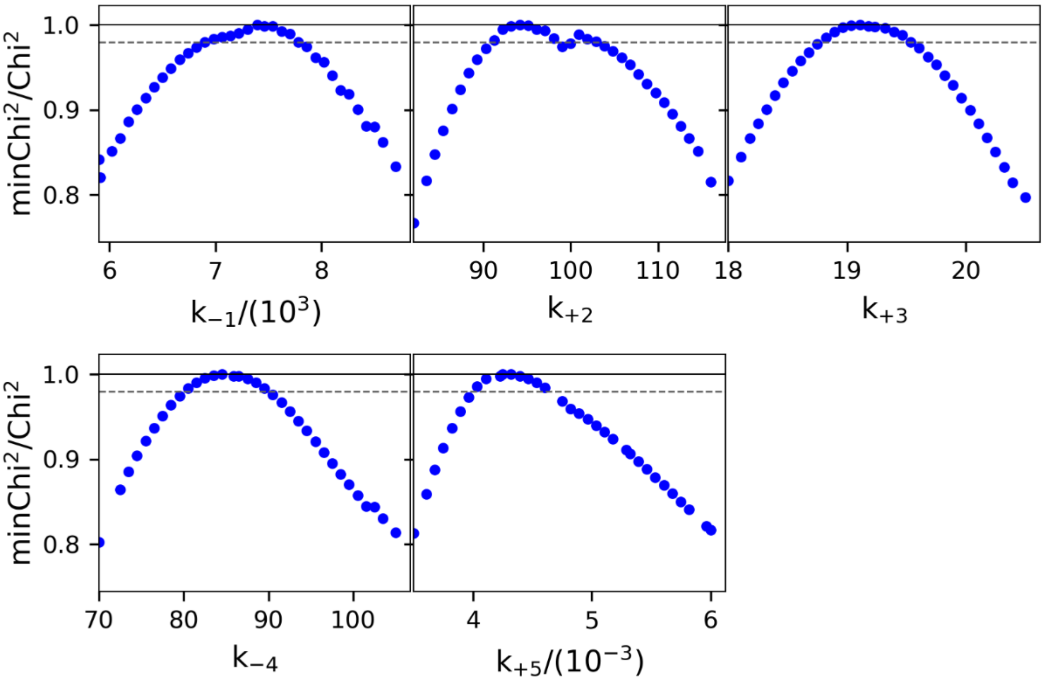

Figure S39: FitSpace 1D confidence contour analysis of the kinetic constants of LinB catalysis. (K_1 = k_{-1}/1000, K_4 = 1000/k_{-4}, k_{inact} = k_{+5}).

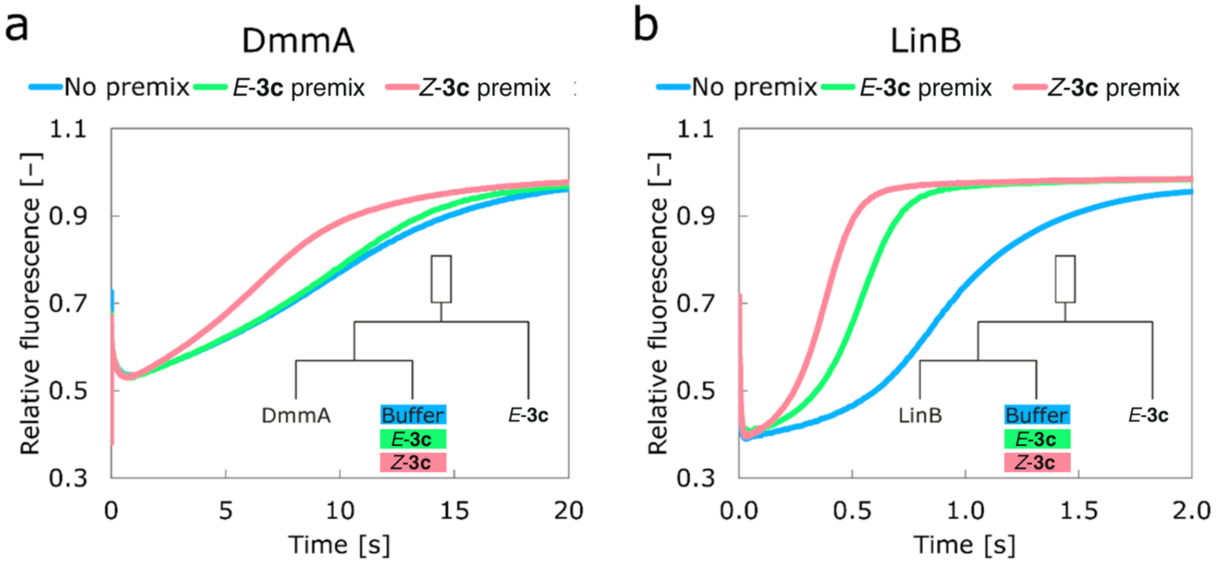

Figure S40: Detection of haloalkane dehalogenase inhibition by *Z*/*E*-**3c**. The experiment was performed in 100 mM glycine buffer pH 8.6 at 37 °C for DmmA (a) and LinB (b). The schemes of various premixing arrangements are provided in the graph inset. Clear significant deviation from the reference curve (blue), observed for premixing with *Z*-**3c**, shows an inhibition effect of the inactive form of the photoswitchable substrate. Minor deviation observed for *E*-**3c** likely corresponds to a small amount of *Z*-**3c** in the substrate sample. The inhibition was substantially more pronounced for LinB in accordance with the global kinetic analysis.

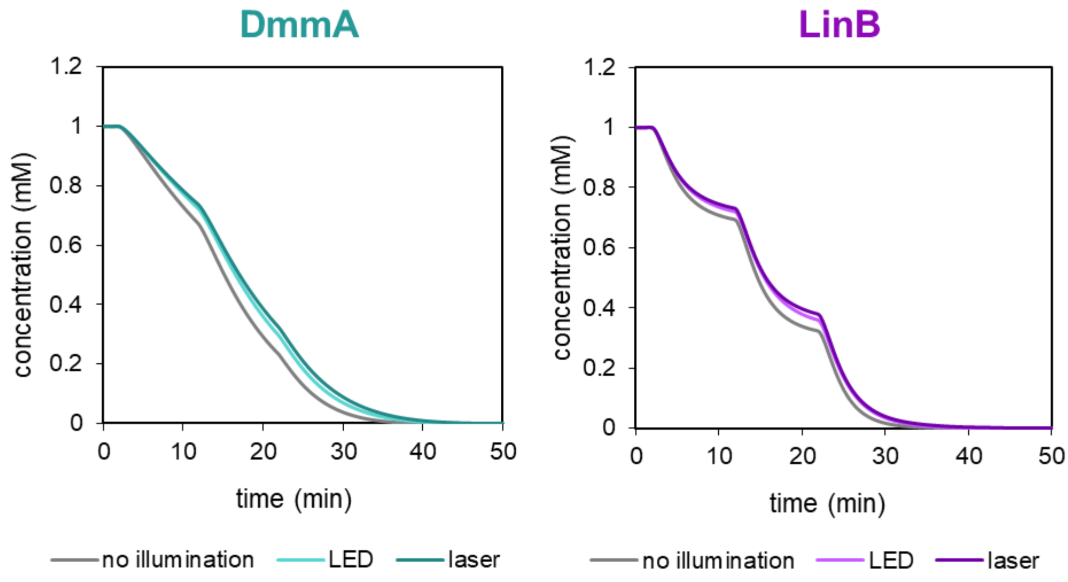

Figure S41: Progress curves of 1 mM *E*-**3c** conversion by DmmA (1 µM per injection) and LinB (0.1 µM per injection) before and after illumination with white laser or LED (420 nm). The experiments were carried out at 37 °C and pH 8.6.

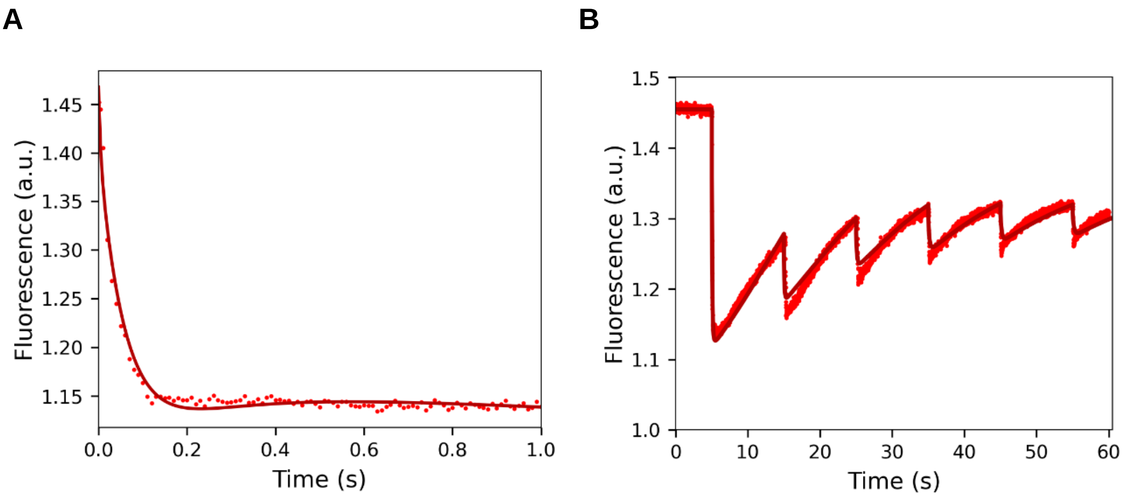

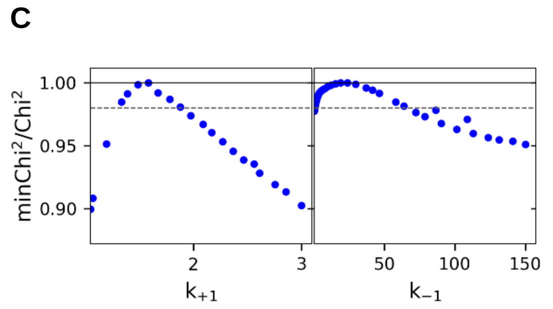

D

parameter	value	s.e.	confidence interval
k_{+1} (μM^{-1}.s^{-1})	1.67	0.06	1.34 - 5.11
k_{-1} (s^{-1})	3.18	1.2	0.43 – 63.8
K_1 (μM)	1.90		

Figure S42: The laser-induced data integrated into the global numerical analysis of the kinetic mechanism of DmmA, providing additional information on substrate association and dissociation kinetics. (A) The initial phase of the reaction was monitored through fluorescence intensity detection (excitation at 275 nm, emission exceeding 320 nm), following the first laser pulse. (B) The entire dataset showing fluorescence intensity recorded upon repeated laser pulses inducing *Z*-to-*E* isomerization in the reaction mixture. The solid lines represent global fitting to the kinetic data. (C) Confidence contour analysis: individual figures represent the dependence of the error on the respective fitted parameter while varying all other parameters to achieve the best fit. In every case, the dashed line shows the χ^2 threshold (0.98) used to establish confidence intervals. (D) The best-fit estimates of the rate of enzyme-substrate complex association (k_{+1}) and dissociation (k_{-1}). The equilibrium constant was calculated from the rate parameters $K_1=k_{-1}/k_{+1}$. The standard error (s.e.) was calculated from the covariance matrix during nonlinear regression. Confidence intervals (lower and upper limits) of the parameters were obtained by confidence contour analysis for χ^2 threshold of 0.98 (Figure S42C).

4. References

1. Gottlieb, H. E., Kotlyar, V. & Nudelman, A. NMR Chemical Shifts of Common Laboratory Solvents as Trace Impurities. *J. Org. Chem.* **62**, 7512–7515 (1997).
2. Ma, J. *et al.* Design, synthesis, and structure–activity relationships of novel benzothiazole derivatives bearing the ortho-hydroxy N-carbamoylhydrazone moiety as potent antitumor agents. *Eur. J. Med. Chem.* **86**, 257–269 (2014).
3. Burkhart, C. & Haberhauer, G. A Light- and Electricity-Driven Molecular Pushing Motor. *Eur. J. Org. Chem.* **2017**, 1308–1317 (2017).
4. Hanwell, M. D. *et al.* Avogadro: an advanced semantic chemical editor, visualization, and analysis platform. *J. Cheminformatics* **4**, 1–17 (2012).
5. Rappe, A. K., Casewit, C. J., Colwell, K. S., Goddard, W. A. & Skiff, W. M. UFF, a full periodic table force field for molecular mechanics and molecular dynamics simulations. *J. Am. Chem. Soc.* (1992) doi:10.1021/ja00051a040.
6. Berman, H. M. *et al.* The Protein Data Bank. *Nucleic Acids Res.* **28**, 235–242 (2000).
7. PyMOL. The PyMOL Molecular Graphics System, Version 2.0 Schrödinger, LLC. (2014).
8. Case, D. *et al. Amber 2014*. (2014).
9. Sanner, M. F. Python: a programming language for software integration and development. *J. Mol. Graph. Model.* **17**, 57–61 (1999).
10. Trott, O. & Olson, A. J. AutoDock Vina: improving the speed and accuracy of docking with a new scoring function, efficient optimization and multithreading. *J. Comput. Chem.* **31**, 455–461 (2010).
11. Hur, S., Kahn, K. & Bruice, T. C. Comparison of formation of reactive conformers for the SN2 displacements by CH3CO2- in water and by Asp104-CO2- in a haloalkane dehalogenase. *Proc. Natl. Acad. Sci.* **100**, 2215–2219 (2003).
12. Daniel, L., Buryska, T., Prokop, Z., Damborsky, J. & Brezovsky, J. Mechanism-Based Discovery of Novel Substrates of Haloalkane Dehalogenases Using in Silico Screening. *J. Chem. Inf. Model.* **55**, 54–62 (2015).

13. Chovancova, E. *et al.* CAVER 3.0: A Tool for the Analysis of Transport Pathways in Dynamic Protein Structures. *PLOS Comput. Biol.* **8**, e1002708 (2012).

14. Klan, P. & Wirz, J. *Photochemistry of Organic Compounds: From Concepts to Practice*. (John Wiley & Sons, Ltd, 2009).
